# Rhenium(I) Tricarbonyl Complexes of 1,10-Phenanthroline Derivatives with Unexpectedly High Cytotoxicity

**DOI:** 10.1021/acs.inorgchem.3c00730

**Published:** 2023-07-25

**Authors:** Lucy E. Enslin, Kallol Purkait, Maria Dalla Pozza, Bruno Saubamea, Pierre Mesdom, Hendrik G. Visser, Gilles Gasser, Marietjie Schutte-Smith

**Affiliations:** † Department of Chemistry, University of the Free State, Bloemfontein 9301, South Africa; ‡ Chimie ParisTech, PSL University, CNRS, Institute of Chemistry of Life and Health Sciences, Laboratory for Inorganic Chemistry, F-75005 Paris, France; § Plateforme Imagerie Cellulaire et Moléculaire, Faculté de Pharmacie, Université Paris Cité, F-75270 Paris, France

## Abstract

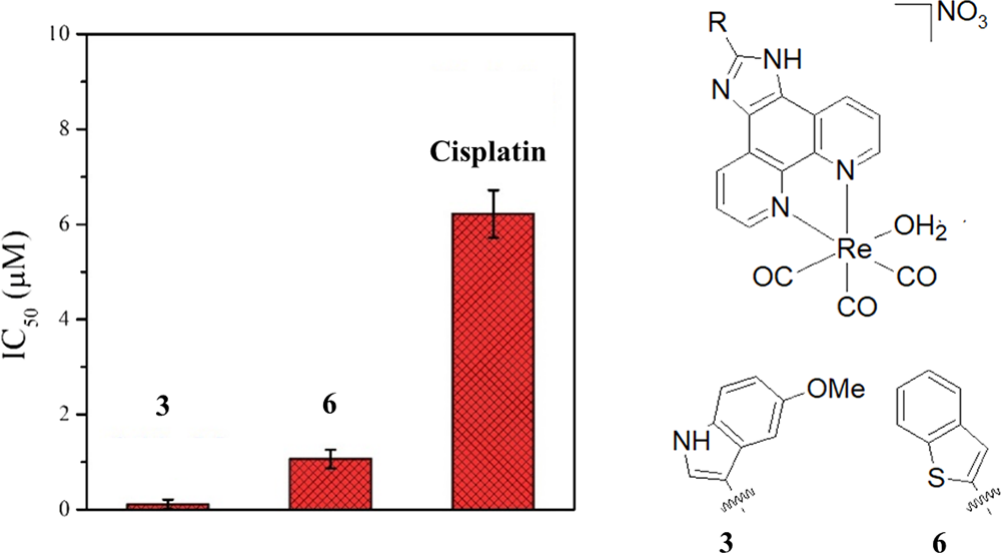

Eight rhenium(I) tricarbonyl aqua complexes with the general formula *fac*-[Re(CO)_3_(*N,N*′-bid)(H_2_O)][NO_3_] (**1–8**), where *N,N*′-bid is (2,6-dimethoxypyridyl)imidazo[4,5-*f*]1,10-phenanthroline (**L1**), (indole)imidazo[4,5-*f*]1,10-phenanthroline (**L2**), (5-methoxyindole)-imidazo[4,5-*f*]1,10-phenanthroline (**L3**), (biphenyl)imidazo[4,5-*f*]1,10-phenanthroline (**L4**), (fluorene)imidazo[4,5-*f*]1,10-phenanthroline (**L5**), (benzo[*b*]thiophene)imidazo[4,5-*f*]1,10-phenanthroline (**L6**), (5-bromothiazole)imidazo[4,5-*f*]1,10-phenanthroline (**L7**), and (4,5-dimethylthiophene)imidazo[4,5-*f*]1,10-phenanthroline (**L8**), were synthesized and characterized using ^1^H and ^13^C{^1^H} NMR, FT-IR, UV/Vis absorption spectroscopy, and ESI-mass spectrometry, and their purity was confirmed by elemental analysis. The stability of the complexes in aqueous buffer solution (pH 7.4) was confirmed by UV/Vis spectroscopy. The cytotoxicity of the complexes (**1–8**) was then evaluated on prostate cancer cells (PC3), showing a low nanomolar to low micromolar *in vitro* cytotoxicity. Worthy of note, three of the Re(I) tricarbonyl complexes showed very low (IC_50_ = 30–50 nM) cytotoxic activity against PC3 cells and up to 26-fold selectivity over normal human retinal pigment epithelial-1 (RPE-1) cells. The cytotoxicity of both complexes **3** and **6** was lowered under hypoxic conditions in PC3 cells. However, the compounds were still 10 times more active than cisplatin in these conditions. Additional biological experiments were then performed on the most selective complexes (complexes **3** and **6**). Cell fractioning experiments followed by ICP-MS studies revealed that **3** and **6** accumulate mostly in the mitochondria and nucleus, respectively. Despite the respective mitochondrial and nuclear localization of **3** and **6**, **3** did not trigger the apoptosis pathways for cell killing, whereas **6** can trigger apoptosis but not as a major pathway. Complex **3** induced a paraptosis pathway for cell killing while **6** did not induce any of our other tested pathways, namely, necrosis, paraptosis, and autophagy. Both complexes **3** and **6** were found to be involved in mitochondrial dysfunction and downregulated the ATP production of PC3 cells. To the best of our knowledge, this report presents some of the most cytotoxic Re(I) carbonyl complexes with exceptionally low nanomolar cytotoxic activity toward prostate cancer cells, demonstrating further the future viability of utilizing rhenium in the fight against cancer.

## Abstract 2

This report presents Re(I) carbonyl complexes with exceptionally low nanomolar cytotoxic activity toward prostate cancer cells, demonstrating further the future viability of utilizing rhenium in the fight against cancer.

## Introduction

Rhenium(I) tricarbonyl complexes are attractive due to a number of potential applications. These include but are not limited to photophysics,^1,2^ artificial photosynthetic materials,^3−5^ supramolecular systems,^6−8^ electrocatalysis,^9,10^ photoinduced catalysis,^9,10^ and nuclear medicine.^11,12^ For decades, organometallic compounds have proven to be auspicious anticancer drug candidates,^13−17^ and as such, complexes may afford diverse mechanisms of action. Rhenium organometallic compounds display several innate properties, such as large Stokes shifts, long-lived emission states, emission tunability through ligand variation, and high quantum yields. These characteristics allow simplified elucidation of the mechanism of action and the cellular distribution of the compounds through emission spectroscopy. Due to their ability to chelate with metal ions such as *N,N′*-donor bidentate ligands, imidazole-phenanthroline derivatives play a pivotal role in biochemistry, analytical chemistry, and electrochemistry.^18−23^ Several synthetic polycyclic compounds of the type [M(L,L′)_2_]^*n*+^, where M is a transition metal ion and L,L′ is an imidazo-phenanthroline-type ligand, have been reported to successfully interact with DNA.^24,25^

An essential requirement of cancer treatments is the ability to induce cancer cell death while preserving the life of healthy non-cancerous cells. In recent studies, the use of rhenium(I) tricarbonyl complexes as possible anticancer agents has shown promising results.^26−31^ Wang *et al*. reported rhenium(I) tricarbonyl complexes with the ability to exert irreversible oxidative stress as well as glutathione metabolism disorder by means of accumulating in the mitochondria.^32^ A rhenium isonitrile complex reported by Wilson *et al*. induces apoptosis by means of an unfolded protein response and additionally shows *in vitro* and *in vivo* anticancer activity toward ovarian cancer cells.^33,34^ Furthermore, they reported the scaffold protein NUBP2 that are involved in Fe–S cluster biogenesis, as a relevant target for a Re(I) complex (TRIP).^35^ Simpson *et al.* designed rhenium(I) complexes that have the ability to induce cell death by means of the inhibition of the phosphorylation of Aurora-A kinase.^36^ Delasoie *et al*. reported a Re(I) tricarbonyl complex that exhibited selectivity toward HCT-116 cells,^37^ while Ye *et al*. reported a rhenium(I)-based histone deacetylase inhibitor with the ability to target mitochondria as well as suppress histone deacetylases activity.^38^

**Figure 1:**
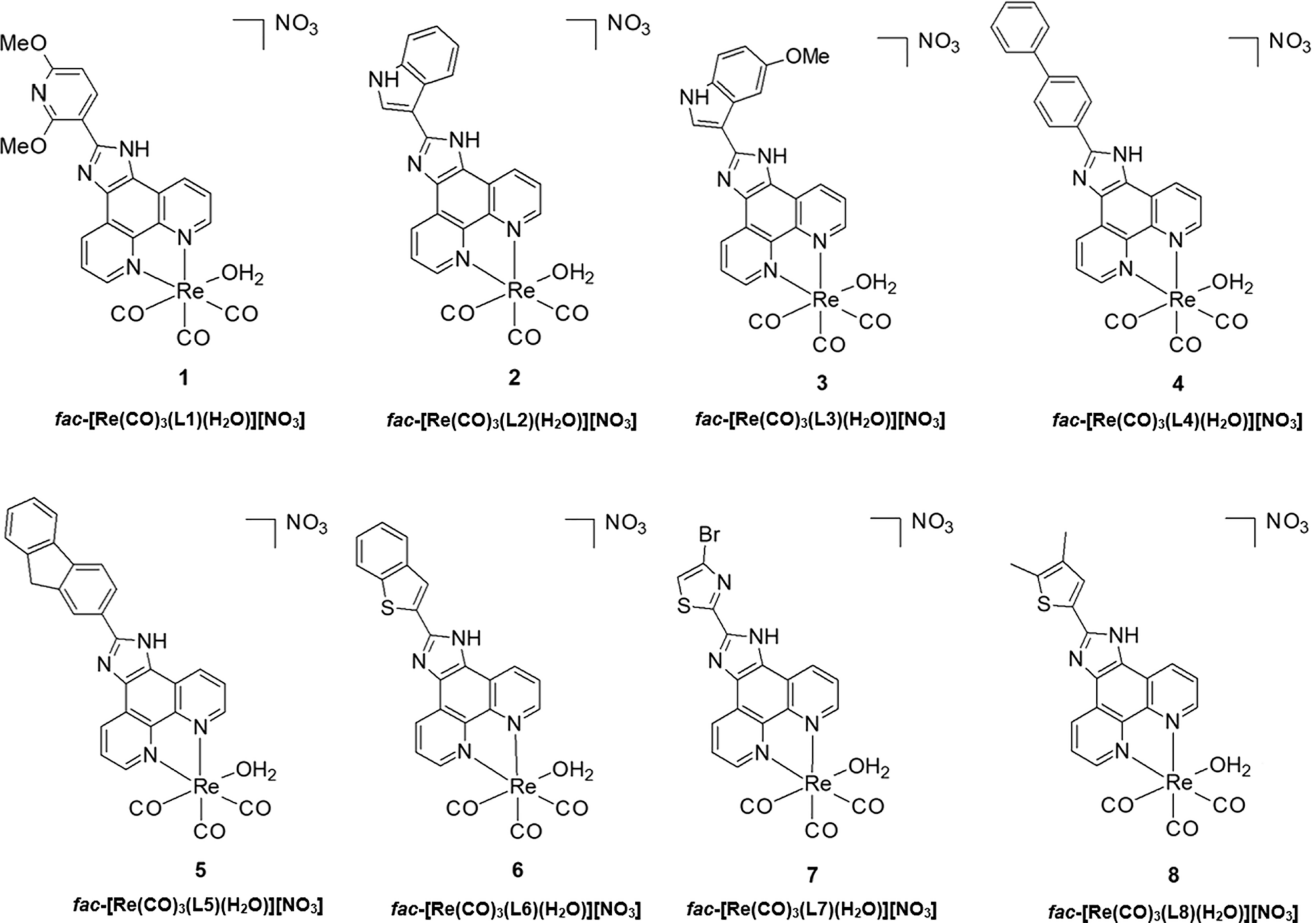
Chemical structures of rhenium(I) tricarbonyl complexes **1–8**.

The development of anticancer agents that exhibit numerous mechanisms of action is of great importance since such compounds may have the ability to overcome typical obstacles such as ineffectiveness toward tumors and drug resistance.^39,40^ A promising strategy in drug development to increase the potential of anticancer drugs in exhibiting multiple mechanisms of action is the coordination of organic molecules with anticancer potential to various metals. With this in mind and the recent success of imidazo[4,5-*f*]1,10-phenanthroline derivatives as prospective anticancer agents in colorectal and liver cancer,^41−43^ we designed a small library of rhenium(I) tricarbonyl complexes (Figure 1) with *N,N′*-bidentate imidazo[4,5-*f*]1,10-phenanthroline derivative ligand systems, with systematic changes with N,S and aromatic motifs on the backbone, having an appended aqua ligand to evaluate their potentials as anticancer agents. Rhenium(I) tricarbonyl complexes are usually kinetically inert, which will help increase the complex half-life to reach the target. The aqua ligand will further increase the possibility of target binding.^44^ The deprotonation of the metal bonded labile aqua ligand and DNA binding ability of the complexes were investigated. Furthermore, we carried out *in vitro* cytotoxicity studies against prostate cancer and normal cells to obtain a small structure–activity relationship (SAR). The cellular localization, cellular uptake pathways, and possible cell killing pathways of the complexes were also studied.

## Results and Discussion

### Synthesis and Characterization

**Scheme 1:**
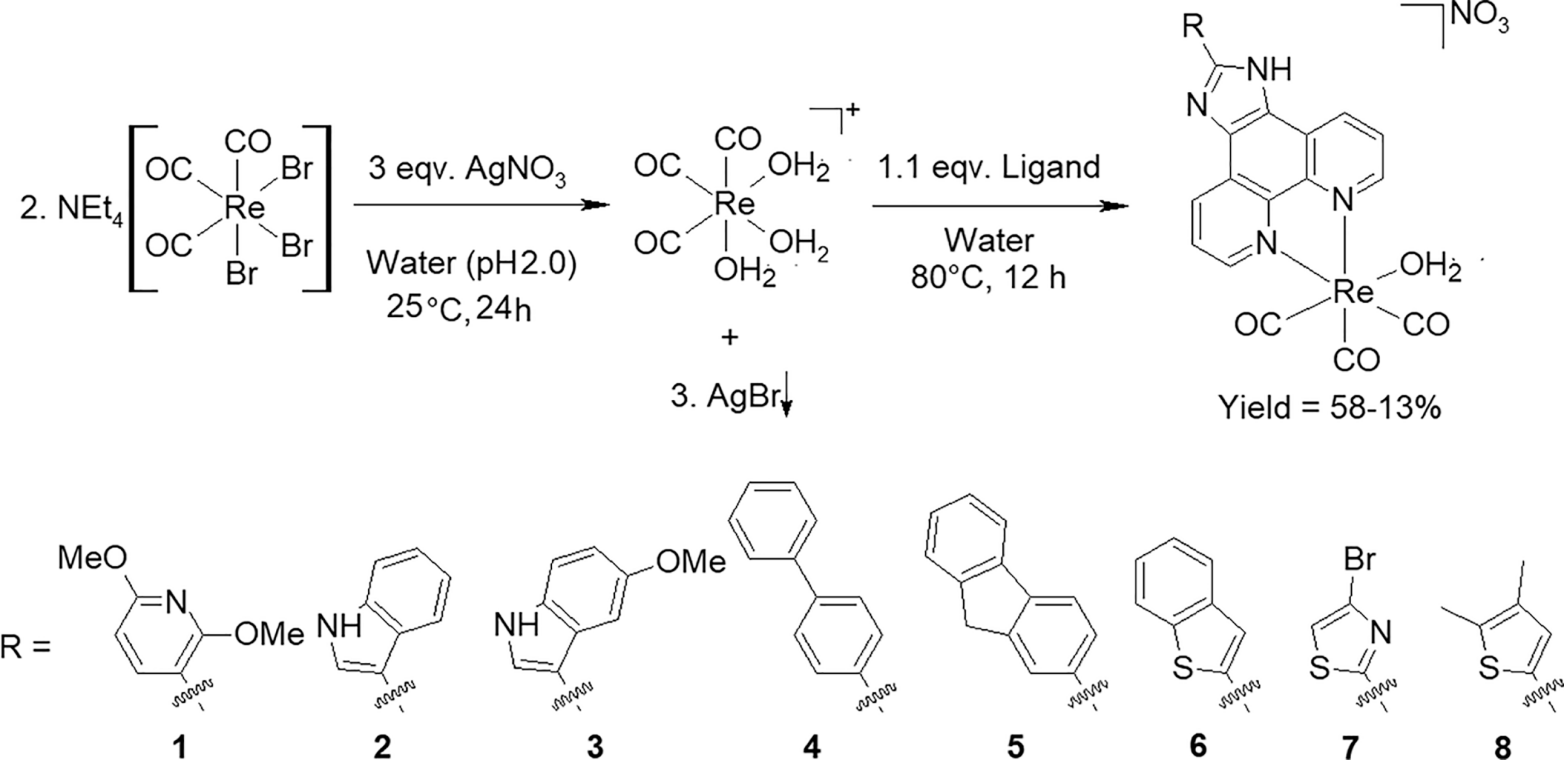
General Synthetic Procedure for Compounds **1**–**8**

The procedure reported by Alberto *et al*. was utilized for the synthesis of the rhenium(I) carbonyl complex-starting synthon *fac*-[NEt_4_]_2_[Re(CO)_3_(Br)_3_] (ReAA).^45^ The characteristic CO stretching frequencies of ReAA were observed at 1864 and 1998 cm^–1^, respectively. The three Br^–^ ligands were replaced by labile aqua ligation *via* the addition of AgNO_3_ at pH 2, resulting in the intermediate *fac*-[Re(CO)_3_(H_2_O)_3_][NO_3_] compound. Water is a good leaving group and can thus be effortlessly replaced by other ligands. Complexes **1**–**8** were obtained by the addition of the respective N,N′ bidentate ligands, viz., (2,6-dimethoxypyridyl)imidazo[4,5-*f*]1,10-phenanthroline (**L1**), (indole)imidazo-[4,5-*f*]1,10-phenanthroline (**L2**), (5-methoxyindole)imidazo[4,5-*f*]1,10-phenanthroline (**L3)** (biphenyl)imidazo[4,5-*f*]1,10-phenanthroline (**L4**), (fluorene)imidazo[4,5-*f*]1,10-phenanthroline (**L5**), (benzo[*b*]thiophene)imidazo[4,5-*f*]1,10-phenanthroline (**L6**), (5-bromothiazole)imidazo[4,5-*f*]1,10-phenanthroline (**L7**), and (4,5-dimethylthiophene)imidazo-[4,5-*f*]1,10-phenanthroline (**L8**), respectively, to the acidic *fac*-[Re(CO)_3_(H_2_O)_3_][NO_3_] solution, producing the *fac*-[Re(CO)_3_(*N,N′*-bid)(H_2_O)][NO_3_]-type compounds. The general synthetic procedure for **1**–**8** is illustrated in Scheme 1. All compounds were characterized using ^1^H and ^13^C{^1^H} NMR, FT-IR, UV/Vis absorption spectroscopy, and ESI-mass spectrometry, and their purity was confirmed by elemental analysis.

### X-ray Crystallography

**Figure 2:**
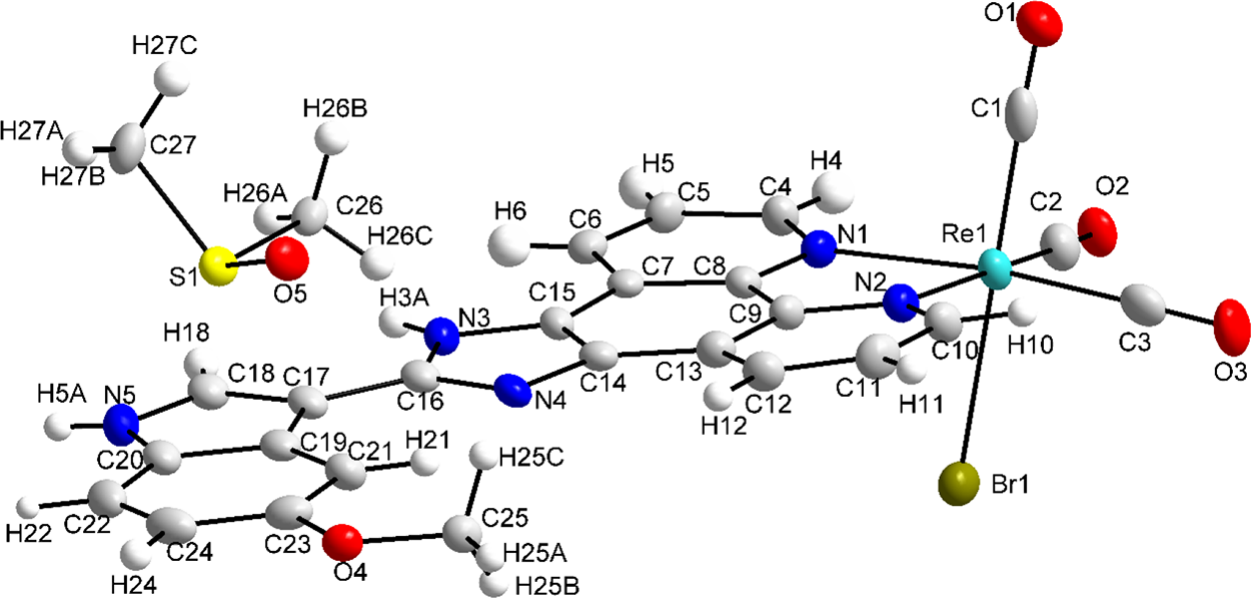
Molecular representation of the crystal structure of *fac*-[Re^I^(CO)_3_(**L3**)(Br)]·DMSO (**3a**). Hydrogen atoms (except two N–H) are omitted for clarity, and ellipsoids are drawn at the 50% probability level.

*fac*-[Re(CO)_3_(**L3**)(Br)] (**3a**) was grown from a solution of *fac*-[Re(CO)_3_(**L3**)(H_2_O)][NO_3_] and NEt_4_Br in DMSO. The aqua complex did not yield any suitable crystals; however, upon substitution of the axial aqua ligand with a bromido ligand, crystals suitable for single crystal crystallography were obtained. The crystallographic data for **3a** is summarized in Table S1, and the molecular diagram of the crystal structure is given in Figure 2. *fac*-[Re(CO)_3_(**L3**)(Br)] (**3a**) crystallized in the triclinic *P*1̅ space group, with one molecule and one solvent dimethyl sulfoxide molecule in the asymmetric unit. The bond distances and angles of **3a** are in the range of similar structures and are reported in Table 1.^46−53^

**Table 1:** Selected Bond Distances and Angles (Å, °) of **3a**

selected bond distances (Å)		selected bond angles (°)	
Re1-C3	1.918(6)	N1-Re1-N2	75.32(16)
Re1-C1	1.905(6)	C3-Re1-C2	87.7(2)
Re1-C2	1.919(6)	C1-Re1-N1	96.03(18)
Re1-N2	2.173(4)	C2-Re1-Br1	91.79(18)
Re1-N1	2.182(4)	C1-Re1-Br1	178.37(17)
Re1-Br1	2.629(10)	C3-Re1-Br1	89.90(16)
		N1-Re1-Br1	84.32(11)
		N2-Re1-Br1	83.98(12)

A dihedral angle of 14.2(2)°, between the equatorial plane (O2–C2–Re1–C3–O3) and the plane through the imidazo[4,5-*f*]1,10-phenanthroline ligand (N1, N2, C1–C12), is observed and expresses the significant bend of the (methoxyindole)imidazo[4,5-*f*]1,10-phenanthroline ligand out of the equatorial plane toward the bromido ligand (Figure S1).

The solid-state structure of **3a** is stabilized by one intramolecular weak interaction (C21–H21···N4), two intermolecular hydrogen bonding interactions (N3–H3A···O5, N5–H5A···Br1), three weak interactions (C11–H11···Br1, C24–H24···O4, and C25–H25C···O5), two Y–X···Cg π-interactions (Re1–Br1···Cg1 and S1–O5···Cg2) with X···Cg distances of 2.947(2) Å and 3.728(5) Å respectively, and an extensive network (forty nine) of π–π-interactions with Cg···Cg distances smaller than 3.8 Å (Tables S2 and S3 and Figure S2). An infinite one-dimensional O–H···O–H chain along the *c* axis is observed that is formed by the two hydrogen bonding interactions (N3–H3A···O5 and N5–H5A···Br1) (Figure S3). The intra- and intermolecular interactions in the crystal structure of **3a** were quantified and confirmed using Hirshfeld surface analysis (Figures S4 and S5). From the fingerprint plots of **3a**, it is clear that the H···H and O···H/H···O interactions contribute significantly to the packing and overall stabilization of the crystal structure (Figure S6).

### Stability Studies of the Complexes

The stability of the complexes in phosphate buffer (pH 7.4) and FBS solution was monitored by UV/Vis absorption measurements over 6 h. No isosbestic point was observed during the measurement of UV/Vis spectra of **1–8** in the buffer solution; however, a time-dependent decrease in absorbance was noticed (Figures S7–S10). This was due to the poor water solubility, which leads to precipitation of the complexes in aqueous solution. It is very important to note that the solubility of the complexes increases significantly in the presence of FBS, as recently observed with Ru(II) polypyridyl complexes (Figure S11).^54^ The stability of complexes **1–8** was also tested in DMSO solution (Figures S7–S10). Multiple isosbestic points were observed in the DMSO solution for complexes **6–8**, suggesting a binding with DMSO, which was not observed for complexes **1–5**. The binding of complexes **6** and **8** with DMSO was also confirmed by ESI-MS measurements (Figure S12). In fact, observing the ESI-MS spectra for both complexes **6** and **8**, we noticed a DMSO–complex adduct ([Re^I^(**L6**/**L8**)(CO)_3_(DMSO)]^+^) as well as a methanolic adduct ([Re^I^(**L6**/**L8**)(CO)_3_(CH_3_OH)]^+^). Moreover, a significant amount of the parent complex without the water ([Re^I^(**L6**/**L8**)(CO)_3_]^+^) was detected (Figure S12). We suggest that the formation of the methanolic adduct is due to the presence of a high amount of methanol in the ESI-MS sample solution, replacing either the water molecule or the DMSO. Unfortunately, due to the poor solubility of complex **7** in methanol, we could not analyze the DMSO binding using ESI-MS.

The stability of the complexes in DMSO was further confirmed by ^1^H NMR; complexes **3** and **6** were dissolved in DMSO for 24 h after which it was dried. The ^1^H NMR spectrum of **3** was the same before and after 24 h in DMSO (Figure S13), confirming the results of the UV/Vis experiments. On the other hand, only 50% of **6** was still intact after it was dissolved in DMSO for 24 h while 50% was the DMSO adduct. We confirmed that ∼70% of the aqua complex **6** is still intact after it was dissolved in DMSO for 6 h (Figure S14).

### Influence of H^+^ Ions – Acid Dissociation Constant Determination

Preliminary cytotoxicity studies indicated that **3** and **6** showed the best cytotoxicity and selectivity. For this reason, it was decided to proceed with **3** and **6** with a more detailed investigation, which also included intracellular accumulation, cellular uptake, mitochondrial respiration, cell death pathways, and ROS generation.

The pH dependence of **3** and **6** was studied at 25.0 °C from pH 5.5 to 11 in DMF:H_2_O 7:3 (*v/v* %) solution. The stability of **3** and **6** in DMF:H_2_O 7:3 (*v/v* %) at pH 5.5 for 24 h was confirmed; the UV/Vis spectra is provided in the Supporting Information (Figures S15 and S16). Equation 1 describes the general acid/base behavior of the monoprotic species HA, while eq 2 was utilized for the determination of the acid dissociation constants of **3** and **6**, where A is the absorbance at the specific pH, A_h_ is the absorbance of the protonated species, A_0_ is the absorbance of the deprotonated species, and *K*_a1_ is the acid dissociation constant.
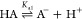
1
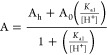
2

**Figure 3:**
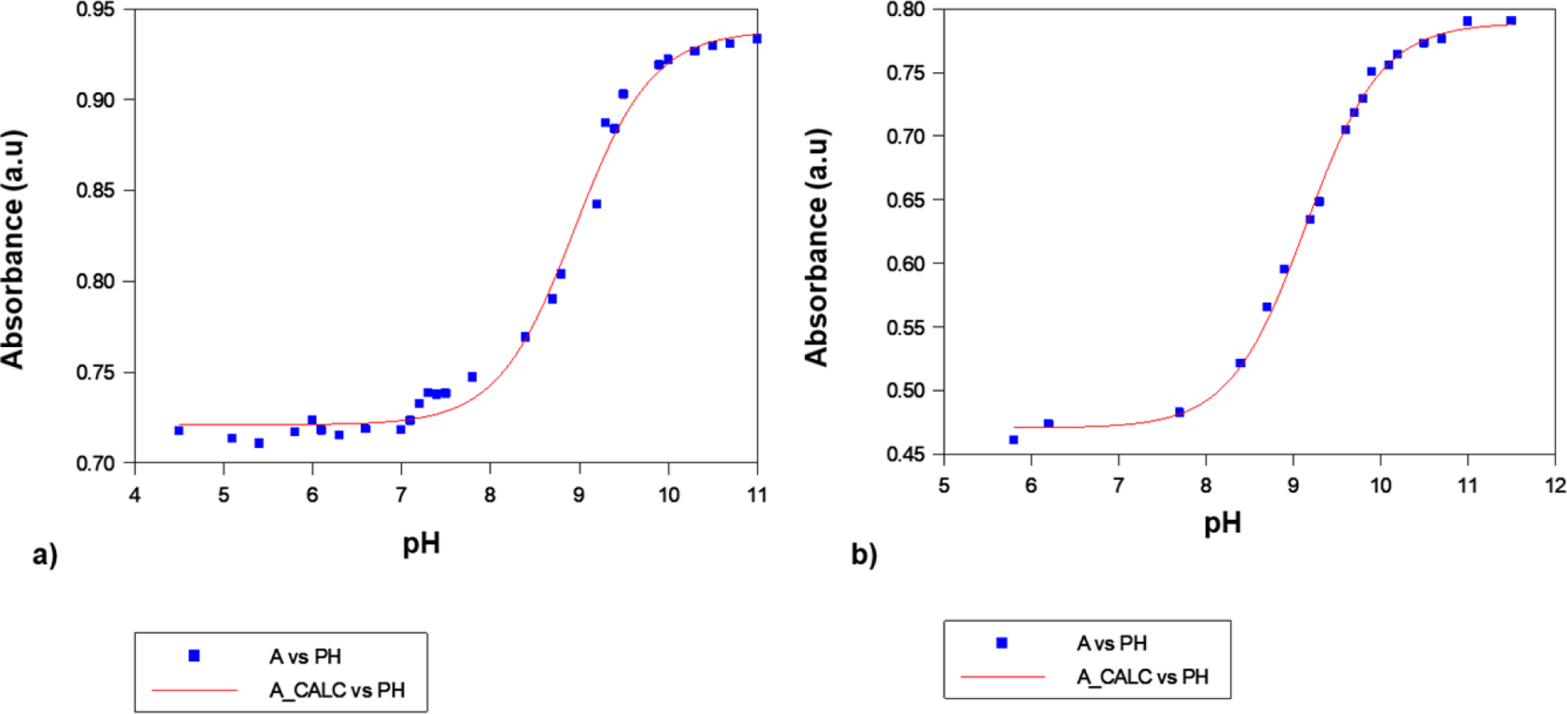
(a) Plot of absorbance *vs* pH for **3**. [Re] = 2 × 10^–5^ M, 25 °C, 367 nm, μ = 1 M (NaClO_4_). (b) Plot of absorbance *vs* pH for **6**. [Re] = 2 × 10^–5^ M, 25 °C, 332 nm, μ = 1 M (NaClO_4_).

The acid dissociation constants of **3** and **6** were determined spectrophotometrically by adjusting the pH of a 2 × 10^–5^ M solution of **3** and **6** at wavelengths 433 and 283 nm, respectively, while measuring the absorbance each time (Figures S17 and S18). The nonlinear fit of this data to eq 2 allowed for determining the p*K*_a1_ values of **3** and **6** as 8.95 (±4) and 9.14 (±2), respectively. This is illustrated in Figure 3. These values correspond with those of similar mononuclear deprotonation products and shows that the complexes are all in the aqua form under physiological conditions.^55−57^ Egli *et a*l.^55^ determined the p*K*_a_ values of *fac*-[Re(CO)_3_(H_2_O)_3_]^+^, yielding the deprotonation products *fac*-[Re(CO)_3_(H_2_O)_2_(OH)] and *fac*-[Re(CO)_3_(H_2_O)(OH)_2_]^−^ to be p*K*_a1_ = 7.5 ± 0.2 and p*K*_a2_ = 9.3 ± 0.3, respectively, while Schutte *et al*.^56^ calculated the p*K*_a_ of *fac*-[Re(CO)_3_(Trop)(H_2_O)] (where Trop = tropolonato) to be 8.96 ± 0.02. The p*K*_a_ values of four rhenium dicarbonyl complexes ([Re(CO)_2_(N,N)(P)(H_2_O)]^+^ with N,N = 1,10′-phenanthroline and 2,9-dimethyl 1,10-phenanthroline, P = PTA and DAPTA) were determined and were in the range 9.09 ± 0.03 to 9.28 ± 0.03. Therefore, no considerable difference is observed in the p*K*_a_ values in these rhenium(I) complexes, whether the ligand *trans* to the aqua ligand is a carbonyl or phosphine ligand or whether the bidentate ligand has N,N or O,O atom donors.

### DNA Binding Study

A preliminary UV/Vis DNA-complex titration study indicated that **6** has the highest binding constant in the series, and later on, we found out that **6** is the most cytotoxic complex in hand. Hence, to check the possible ways of DNA binding by **6**, we have performed (a) a binding study with a model nucleobase (guanosine) by ^1^HNMR and ESI-MS for a possibility of covalent binding, (b) an ethidium bromide displacement assay for intercalation, and (c) a DNA melting assay to confirm the binding ability.

Surprisingly, in the^1^H NMR spectra of guanosine binding kinetics, we only observed a secondary interaction of **6** with guanosine, despite **6** having a labile neutral water molecule in the Re center (Figures S19–S22). The binding study between the Re center of complex **6** and N7 of guanosine was monitored by ^1^H NMR in DMF at 37 °C (Figures S19–S22). From the initial time (1 h), we observed a 0.015 ppm downfield shift of the H_8_ peak along with the shift of H_b_ and H_c_. Considering H_a_, we did not find any shift during the course of the study. This would suggest that the shift of the mentioned protons (H_b_, H_c_, and H_8_) is due to some secondary interaction but not to the binding with N7 of guanosine with the Re center. Moreover, the amine of guanosine is not taking part in the secondary noncovalent interaction (Figure S20). When we analyzed the same solution by ESI-MS, we found a little peak of **6** and a guanosine adduct along with an important amount of free ligand (Figure S23). However, we could not find any alteration of ^1^H NMR peaks of **6** with time (Figure S19) and the peaks were not shifted in the ^1^H NMR spectra studying the stability of **6** (Figure S23). Hence, the formation of the adduct and the degradation of **6** have occurred only in the ESI-MS measurement conditions. Therefore, we deduced that the complex cannot form any covalent bond with DNA *via* the Re center.

**Figure 4:**
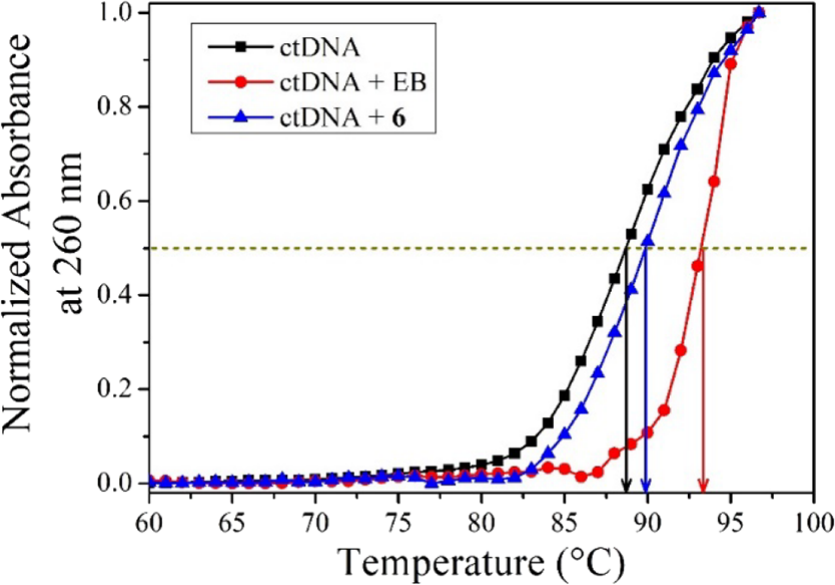
ctDNA melting curve. Conc. of ctDNA was 40 μM, conc. of EB and **6** was 30 μM. Temperature: 60–97 °C; holding time: 1 min, 1 °C/min and the abs was recorded with every 1 °C change in temperature.

The presence of a planar ring system increases the possibility of DNA intercalation. Hence, to measure the intercalation ability of **6**, we performed an ethidium bromide (EB) displacement assay by fluorescence quenching of the EB-DNA adduct (Figure S24). The apparent binding constant for intercalation of **6** with ctDNA is in the order of 3.9 (1) × 10^6^ M^–1^, suggesting the possible targeting of DNA via intercalation. Finally, the DNA binding properties of complex **6** was confirmed by measuring DNA melting temperature (Figure 4). We observed a shift of ctDNA melting temperature toward higher temperature of **6**-bound ctDNA (Δ*T*_m_; 1 ± 0.4 °C and 4 ± 0.1 °C for **6** and EB, respectively, (Figure 4)) in comparison to unbound/normal ctDNA melting temperature.

### Distribution Coefficient (log *P* Values)

**Figure 5:**
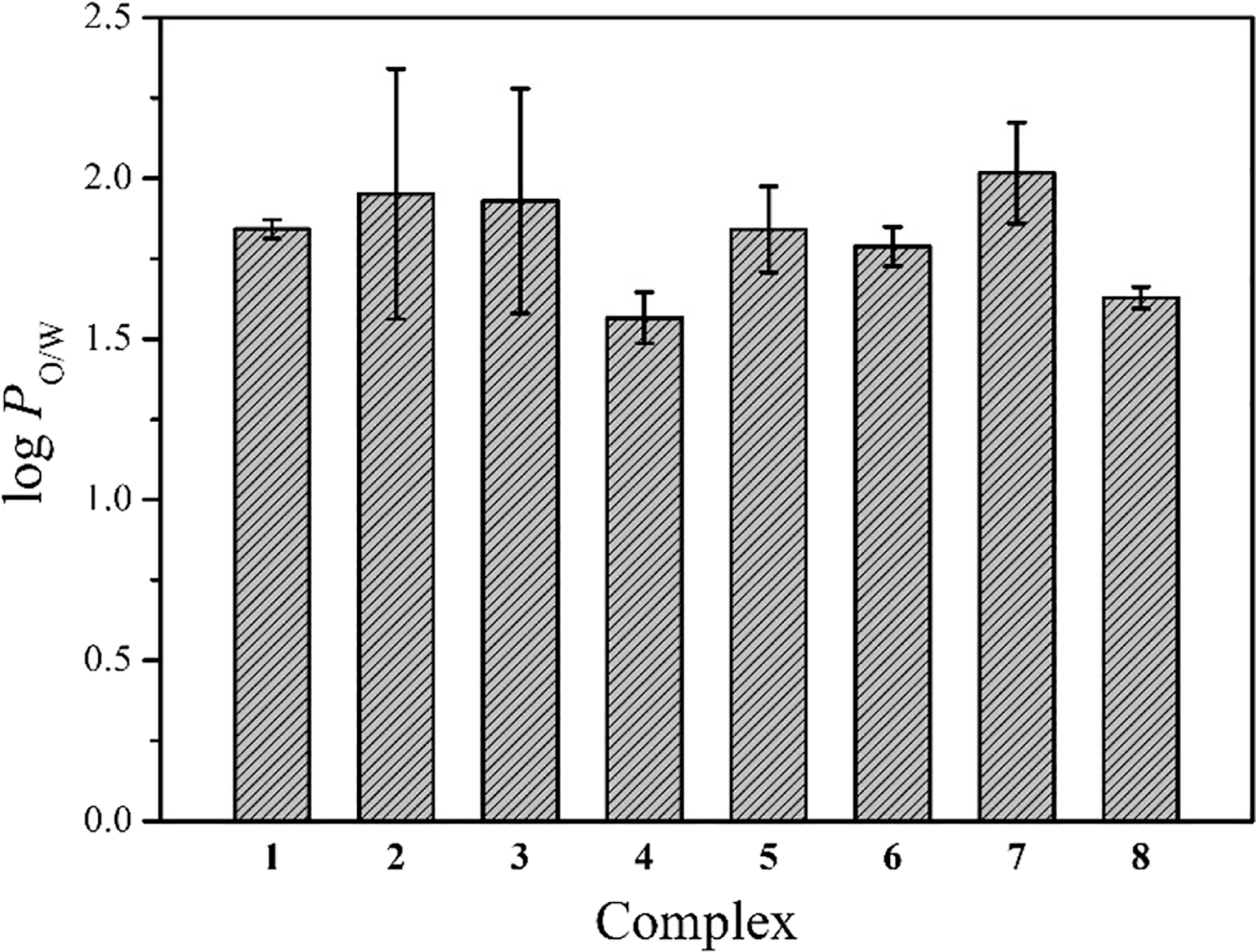
Bar plot representing the log *P* values of complexes **1–8**.

The distribution coefficient (log *P* value) of the complexes was determined using the standard shake flask method.^58^ All the complexes showed almost equal distribution coefficients within 1.5–2.0 (Figure 5), suggesting hydrophobicity. This suggests that the derivatization on the N–N bidentate ligand of Re complexes might not be very useful to change the solubility and lipophilicity of the compounds. Overall, the log *P* values of our complexes compared well with other Re tricarbonyl compounds reported in the literature.^30,59^

### BSA Binding

Serum albumin is the most abundant protein present in the blood and plays an important role in the delivery of hydrophobic compounds to the cell. Importantly, serum albumin contains a free thiol from 34-cysteine^60−63^ and Re complexes have very high binding affinity for sulfur donors.^64,65^ BSA has an intrinsic fluorescence emission maximum at 341 nm upon excitation at 280 nm.^66,67^ The internal fluorescence quenching was observed upon binding with our compound. Hence, fluorescence titrations between BSA and different concentrations of the complex were performed to measure the binding constant. The bovine serum albumin (BSA) binding study suggests that all complexes can bind BSA with a moderately high binding constant in the order of 10^5^ M^–1^ except for complex **7** (Table 2, Figures S25–S28). A significantly low binding constant was observed for **7** (0.4 × 10^5^ M^–1^). The Stern–Volmer quenching constant signifies the distance between an inhibitor and the fluorescent molecules.^68−70^ The decrease of *K*_sv_ indicates a long distance between the inhibitor and fluorescence protein. We observed the *K*_sv_ in the order of 10^4^ M^–1^, suggesting a moderate fitting in the hydrophobic pocket of BSA.

**Table 2:** Affinity of Complexes **1–8** for BSA^a^

complex	Stern–Volmer quenching constant (*K*_sv_ × 10^4^, M^–1^)	binding constant (*K*_a_ × 10^5^, M^–1^)	number of binding sites (*n*)
**1**	2.41 ± 0.05	2.71 ± 2.91	1.23 ± 0.13
**2**	1.06 ± 0.03	1.10 ± 0.79	1.24 ± 0.08
**3**	2.98 ± 0.15	1.81 ± 1.16	1.18 ± 0.07
**4**	2.95 ± 0.09	1.46 ± 0.06	1.18 ± 0.01
**5**	2.57 ± 0.07	1.04 ± 0.30	1.15 ± 0.03
**6**	2.82 ± 0.07	2.29 ± 0.98	1.23 ± 0.05
**7**	0.83 ± 0.03	0.40 ± 1.16	1.16 ± 0.03
**8**	1.60 ± 0.10	0.94 ± 1.11	1.10 ± 0.20


a All plots are available in Figures S25–S28.

### Cytotoxicity

The *in vitro* anticancer activity of the complexes was determined against human prostate cancer (PC3) and compared with the normal retinal pigment epithelial-1 (RPE-1) cells. The results are summarized in Table 3. Among the eight complexes, **4**–**6** showed cytotoxicity in the low nanomolar range (0.04, 0.03, and 0.05 μM, respectively). Such low values are scarce in the literature for similar compounds. However, the three complexes are highly cytotoxic against normal RPE-1 cells as well (0.12, 0.27, and 0.65 μM, respectively). Despite this, it is very exciting to notice that almost all complexes are more toxic against cancer cells than normal cells. Complex **3** is the most promising by showing a 26-fold selectivity with an IC_50_ value of 0.32 μM against PC3 cells. Additionally, it is almost 21 times more cytotoxic than cisplatin against PC3 cells. Interestingly, it was observed by ICP-MS that the most selective complexes **3** and **6** were localizing in the mitochondria and nuclei, respectively, suggesting that they have a different mode of action. In general, we observed that a fused ring or similar derivatization in the N–N bidentate ligand could be useful to increase cytotoxicity. Very interestingly, comparing complexes **2** and **3**, the introduction of a methoxy moiety in the indole ring of **2** showed a positive impact on the IC_50_ values with an increase of almost 15-fold in cytotoxicity.

**Table 3:** Cytotoxicity of Complexes **1**–**8** under the Normoxic Conditions in Comparison to Cisplatin

	IC_50_ (μM) ± S.D.^a^		
complex	PC3	RPE-1	selectivity^b^
**1**	4.6 ± 0.5	0.36 ± 0.02	
**2**	5 ± 3	23 ± 2	5
**3**	0.32 ± 0.03	8.2 ± 0.4	26
**4**	0.04 ± 0.01	0.12 ± 0.01	3
**5**	0.03 ± 0.01	0.27 ± 0.02	9
**6**	0.05 ± 0.01	0.65 ± 0.03	13
**7**	1.4 ± 0.40	4.2 ± 0.5	3
**8**	0.41 ± 0.04	1.79 ± 0.04	4
cisplatin	6.9 ± 0.5	36 ± 4	5

a S.D. is the standard deviation. The IC_50_ value was determined using the standard Resazurin assay after 48 h of treatment under normoxic conditions (∼15% O_2_ level). The representative plots are provided in Figures S29–S33.

b Selectivity represents the ratio between IC_50_ against RPE-1 and IC_50_ against PC3 cells.

**Figure 6:**
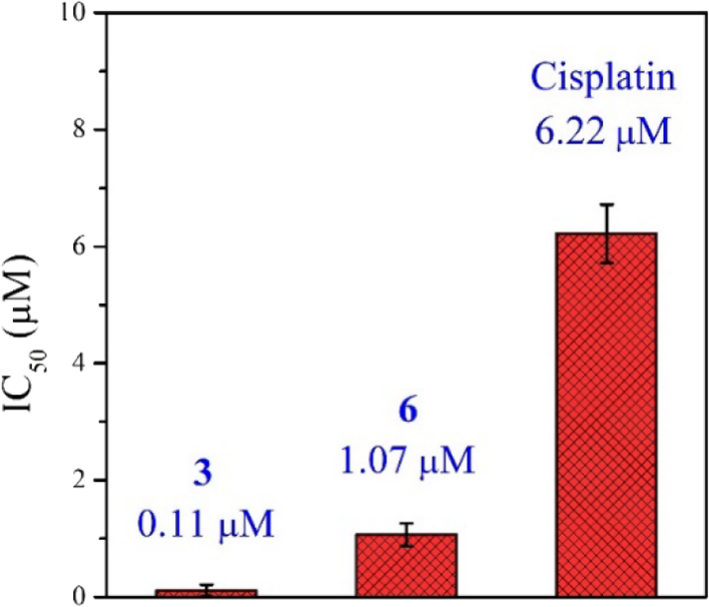
IC_50_ values represent the cytotoxicity of complexes against 3D multicellular PC3 cells spheroid (average diameter of 435 μm) after 72 h of incubation with the respective complexes in normoxic conditions, determined using the AlamarBlue assay.

Despite the high cytotoxic activity of complexes against PC3 cells in normoxia, a decrease in cytotoxicity (IC_50_, μM: 5.5 ± 1.3 for **3** and 7.0 ± 2.2 for **6**) was observed for complexes **3** and **6** when they were evaluated in hypoxic conditions (Table S4). However, a decrease in cytotoxicity of cisplatin (IC_50_, >50 μM) in hypoxic conditions was also observed. Hence, in hypoxic conditions, despite the decreased cytotoxicity of **3** and **6** against PC3 cells, they were still around 10 times more active than cisplatin. Very importantly, when the compounds were tested against 3D multicellular PC3 cell spheroids, we observed a small decrease in IC_50_ concentration (Figure 6), indicating that the compounds are efficient in penetrating the more tumor-like environment of cell spheroids.

### Intracellular Accumulation Study

**Figure 7:**
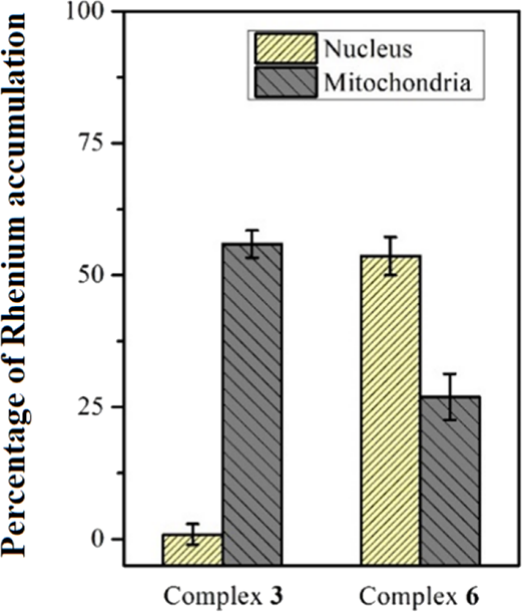
Percentage of Re(I) accumulation in the nucleus and mitochondria compared to total uptake by PC3 cells. The amount of rhenium in the isolated nucleus and mitochondria from complex-treated PC3 cells was quantified using ICP-MS.

Most of the rhenium(I) carbonyl complexes reported so far preferentially target mitochondria.^14,32,59,71^ In order to investigate the possible cellular target of the complexes, we measured the accumulation of complexes **3** and **6** in the nuclei and mitochondria using cell fractionation and ICP-MS and not using the intrinsic luminescent properties of the Re(I) tricarbonyl complex. In fact, it was reported that different cellular accumulation results were observed between these methods since the luminescence properties of this type of metal complex are environment-dependent and can alter the confocal results.^72−74^ We found that **3** targets the mitochondria, whereas **6** mostly targets the nuclei and, to a lesser extent, the mitochondria (Figure 7). This observation is interesting since there are very few examples of Re(I) tricarbonyl complexes that target the nuclei.

### Cellular Uptake and Uptake Pathways

**Figure 8:**
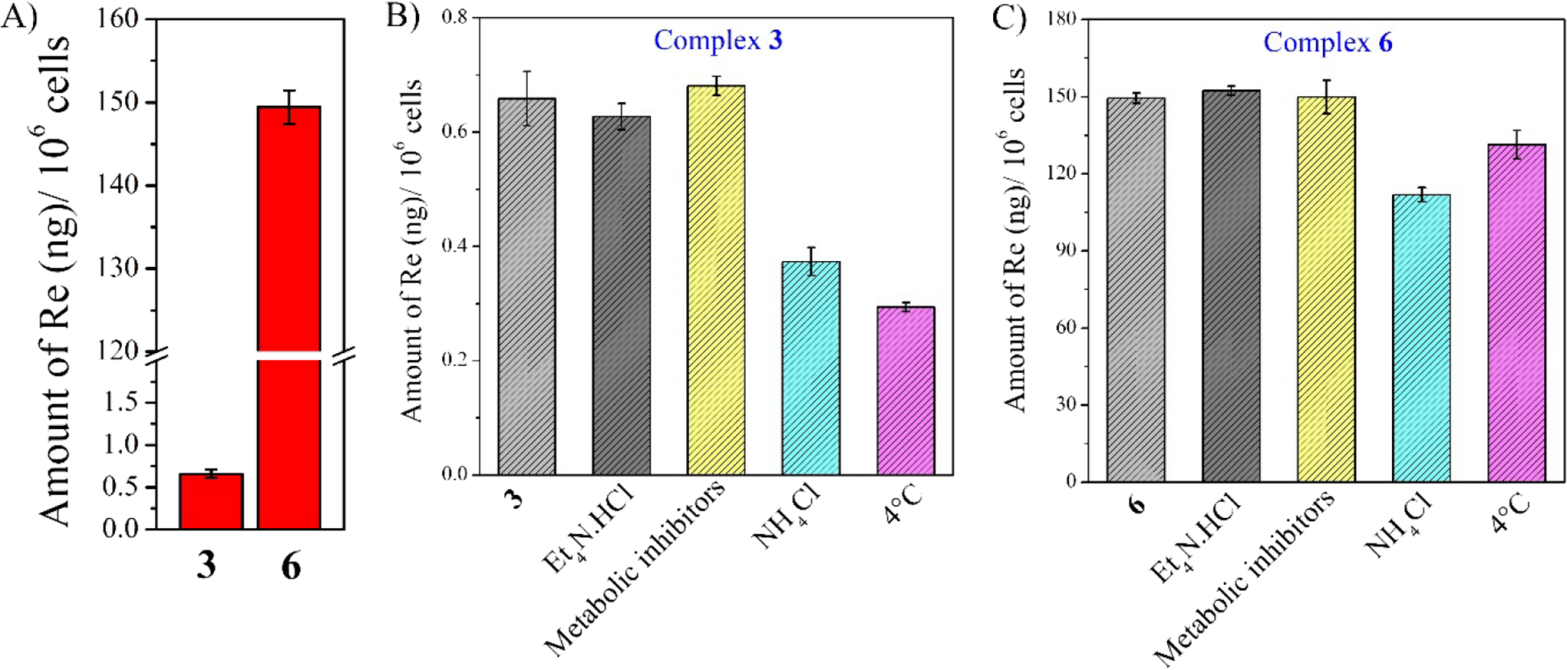
(A–C) Amount (ng) of rhenium in PC3 cells upon 2 h of incubation with either complex **3** or **6** in the presence and absence of cellular uptake pathways inhibitor. The amount of Re was quantified by ICP-MS. 1 mM tetraethylammonium chloride (cationic transporter inhibitor), 5 μM oligomycin and 50 mM 2-deoxy-d-glucose (metabolic inhibitors), and 50 mM ammonium chloride (endocytotic inhibitor) were used.

The cellular uptake pathways of complexes **3** and **6** have been investigated using known uptake pathways inhibitors, viz., 1 mM tetraethylammonium chloride (cationic transporter inhibitor),^75^ 5 μM oligomycin and 50 mM 2-deoxy-d-glucose (metabolic inhibitors),^76,77^ and 50 mM ammonium chloride (endocytotic inhibitor).^78−80^ The cells were pre-treated with the respective inhibitors to block the transporter channel. Hence, the uptake of complexes might get affected if the complex utilizes those transporters. For both complexes **3** and **6**, a significantly less amount of complex uptake was observed in the presence of an endocytotic inhibitor (i.e., ammonium chloride)^78−80^ and at 4 °C in comparison to the control (Figure 8B,C). Hence, the result suggests that endocytotic and passive diffusions were majorly involved in complex uptake. Surprisingly, a significantly high uptake of complex **6** was observed compared to **3**, which could explain the low nanomolar cytotoxicity of **6** (Figure 8A).

### Mitochondrial Respiration Test

**Figure 9:**
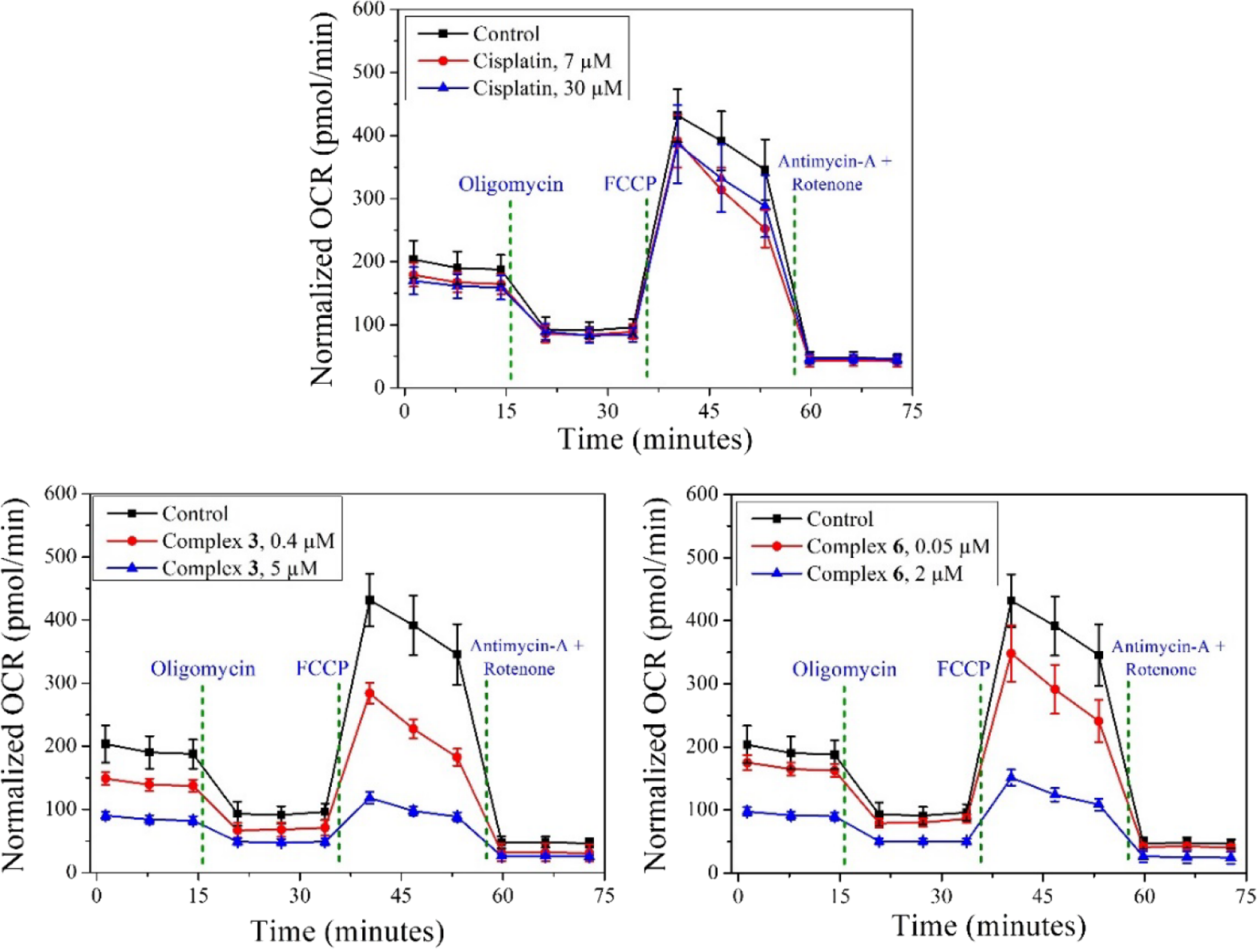
Mito stress test profile in PC3 cells measured by a Seahorse XeF4 after 24 h treatment with complexes **3** and **6** and cisplatin in normoxic conditions. OCR stands for oxygen consumption rate. The experiment was performed using specific electron transport chain inhibitors, *viz.*, oligomycin (inhibitor of ATP synthase (complex V)), FCCP (uncoupling agent), antimycin-A (complex III inhibitors), and rotenone (complex I inhibitor).

Since a significant amount of the complexes **3** and **6** accumulated in the mitochondria, the possible influence of the compounds in the PC3 cells’ oxygen consumption was measured by the Mito stress test using a Seahorse XF Analyzer. In both cases, a significant amount of concentration-dependent mitochondrial dysfunction was identified. Initially, a concentration-dependent lowering of basal respiration was observed for both complexes **3** and **6** (Figure 9 and Figure S34). Furthermore, a significant drop in ATP production was identified (Figure S35).

### Cell Death Pathways and ROS Generation

**Figure 10:**
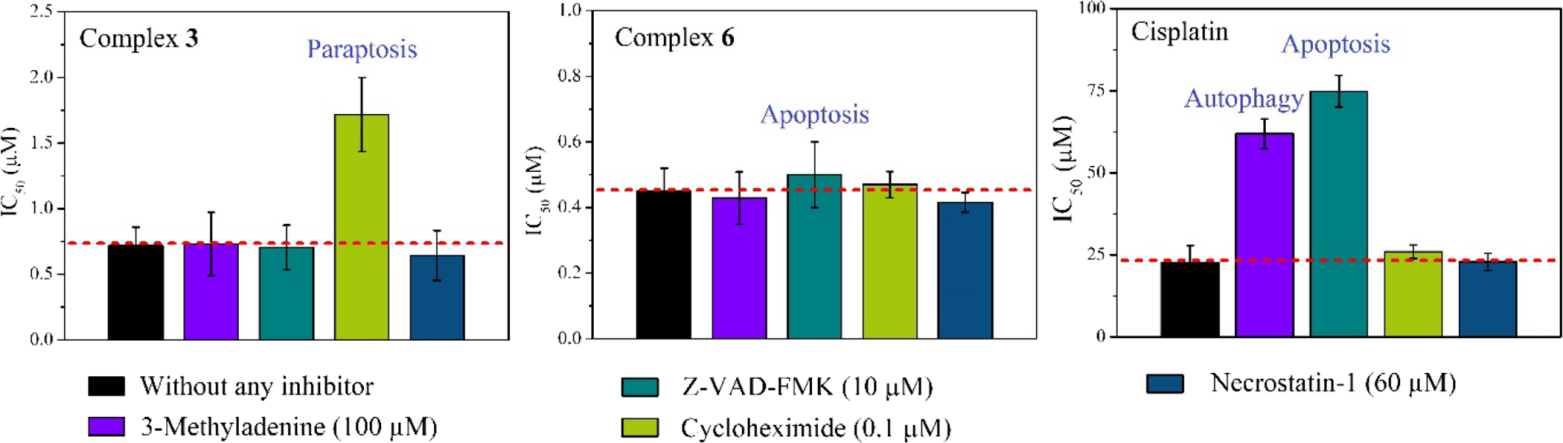
IC_50_ values of respective complexes against PC3 cells in the presence of inhibitors in comparison to the cytotoxicity without any inhibitor. The inhibitors were pre-incubated for 1 h. The respective metal complexes were then loaded and incubated for 36 h in normoxic conditions (details in the Experimental section).

**Figure 11:**
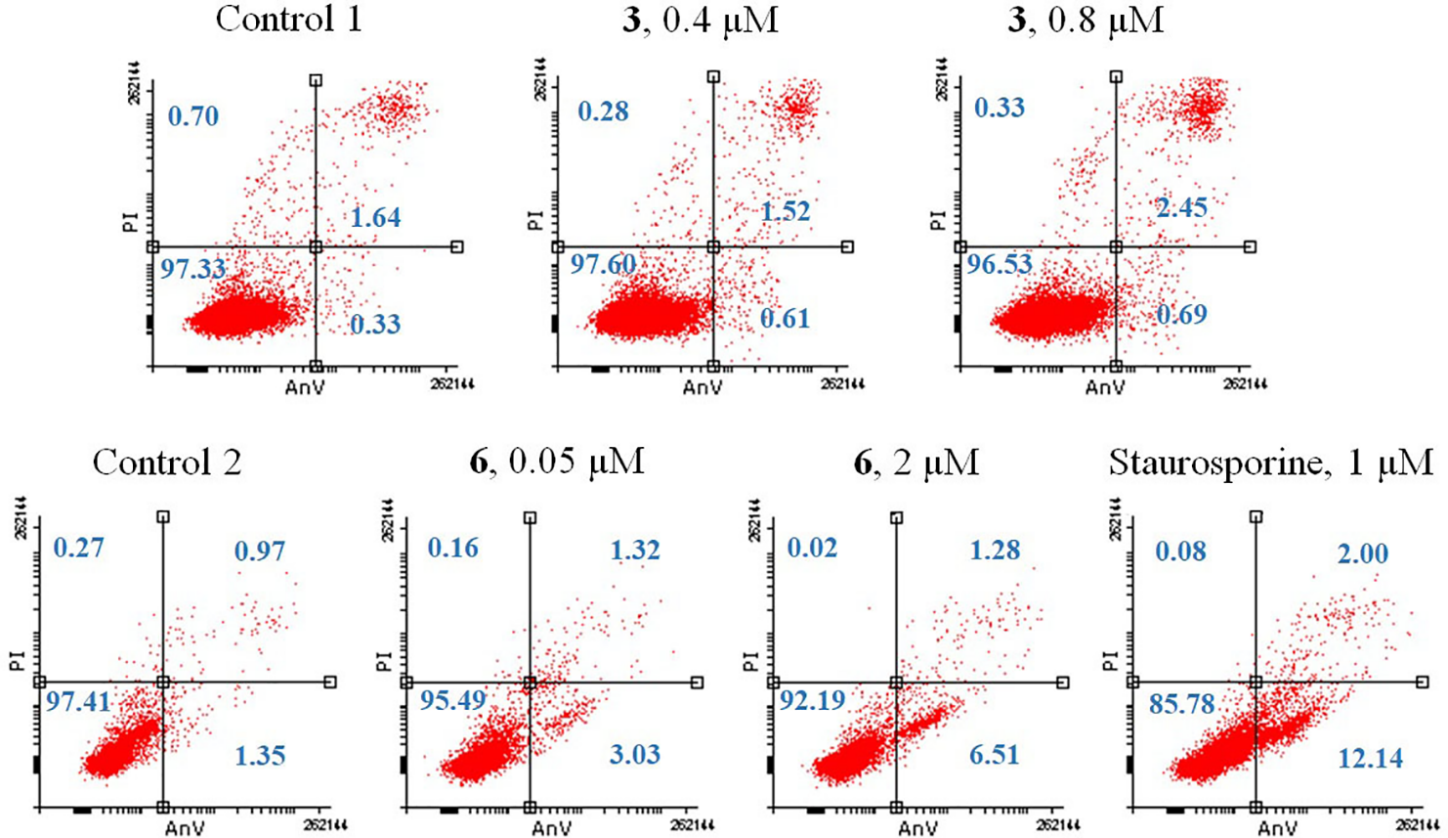
Induction of apoptosis of compound-treated PC3 cells were measured by flow cytometry after 24 h compound exposure. The compound-treated cells were stained dually with Annexin V-PE and PI prior to analysis. Lower left quadrant (both Annexine V and PI-negative fraction): intact cells. Lower right quadrant (Annexine V-positive/PI-negative fraction): early apoptosis. Upper right quadrant (Annexine V-positive/PI-positive fraction): late apoptosis. Upper left quadrant (Annexine V-negative/PI-positive fraction): necrosis. Staurosporine, an apoptosis inducer, was used as positive control.

The cell death pathways induced by **3** and **6** in PC3 cells were analyzed by measuring cytotoxicity in the presence of specific cell death pathway inhibitors, viz., 3-methyladenine (autophagy), Z-VAD-FMK (apoptosis), cycloheximide (paraptosis), and necrostatin-1 (necrosis). A significant amount of increase in the IC_50_ value (around 2 times) of complex **3** against paraptosis inhibitor-treated PC3 cells was observed (Figure 10). Finally, the paraptosis induction in PC3 cells by complex **3** was confirmed by observing vacuole formations in the bright field image of PC3 cells treated with compound **3** (SI, Figure S36). Of note, similar kinds of Re complexes are known to induce cell death by paraptosis.^38,59^ In the case of complex **6**, a very little decrease in IC_50_ dose was noticed when the cells were pre-treated with an apoptosis inhibitor (Figure 10). Hence, we performed the well-known annexin-V assay to confirm the apoptosis pathway. As we found a non-apoptosis pathway for cell killing during IC_50_ dose determination of **3** in the presence of an apoptosis inhibitor (Figure 10), complex **3** did not induce apoptosis, whereas for complex **6**, a concentration-dependent induction of apoptosis was observed (Figure 11). However, the amount of apoptosis induction by **6** was very low (Figure 11), suggesting that apoptosis may not be a major pathway for cell killing. In the case of cisplatin, we observed an important increase of the IC_50_ value in the presence of both apoptosis and autophagy inhibitors (Figure 10). The observed apoptosis and autophagy induction by cisplatin agrees with literature reports.^81^

Metal complexes are very well-known to generate ROS due to the presence of easily accessible multiple oxidation states. The generation of ROS inside the PC3 cells treated with the two Re derivatives was analyzed using a standard ROS detection kit. The result suggested that the compounds do not play a role in altering the ROS homeostasis of the cells. In fact, after treatment with complexes **3** and **6**, the ROS level (Figure S37) inside the cells was similar to the control.

## Conclusions

This study reports the synthesis, characterization, DNA binding mode, and *in vitro* cytotoxicity of eight rhenium(I) tricarbonyl complexes. All complexes showed stability in aqueous solution and can bind to BSA with binding constants ranging from 10^4^ to 10^5^ M^–1^. The p*K*_a_ values of **3** and **6** indicate that these complexes will be in the aqua form under biological conditions. The Re(I) carbonyl complexes showed exceptionally low nanomolar cytotoxicity against prostate adenocarcinoma (PC3) and a degree of selectivity toward cancer cells over normal human retinal pigment epithelial-1 (RPE-1) cells. The most selective complexes **3** and **6** were found to localize in mitochondria and nuclei, respectively. Moreover, both complexes **3** and **6** downregulated the mitochondrial ATP production in PC3 cells. The most selective complex **3** followed a paraptosis pathway for cell killing. On the other hand, despite the intercalation of complex **6** with DNA and the induction of mitochondrial dysfunction after treatment, only a very little percentage of cells induced apoptosis. This suggests that apoptosis is a minor pathway for cell killing. Surprisingly, **6** did not follow any of the other tested cell death pathways, i.e., necrosis, autophagy, or paraptosis. Very interestingly, this suggests that the presence of different functional groups on the same phenanthroline ligand core could induce different cell death mechanisms, while maintaining a very promising IC_50_ range.

## Experimental Section

### General Procedures and Instrumentation

The IR spectra were recorded at room temperature on a PerkinElmer BX II IR spectrometer in the range 4000–370 cm. The liquid-state ^1^H and ^13^C{^1^H} NMR spectra were recorded at 25.0 °C on a 600 MHz Avance II Bruker spectrometer operating at 600 and 151 MHz for ^1^H and ^13^C, respectively, and dimethyl sulfoxide-*d*_6_ (DMSO-*d*_6_) was used as solvent. The chemical shifts (δ) are reported in parts per million (ppm); for DMSO-*d*_6_, the spectra were referenced relative to the solvent peak (2.50 ppm for ^1^H and 39.52 ppm for ^13^C). Coupling constants (*J*) are reported in Hz. Electrospray ionization mass spectrometry (ESI-MS) of **1**–**8** was recorded on a SCIEX 4000 QTRAP hybrid triple quadrupole ion trap mass spectrometer either in positive or in negative ESI mode with samples injected as methanol solutions. The ionization voltage was set at 5500 V in positive mode with a 10 psi curtain gas setting and 20 psi ionization gas (GS1) setting. The UV/Vis spectra were recorded in the range of 100–1100 nm at room temperature on a LAMBDA 265 UV/Visible spectrophotometer. The spectrometer utilizes a Xenon Flash interface. The intensity data was collected on a Bruker X8 ApexII 4 K Kappa CCD diffractometer, equipped with graphite monochromated Mo K(α) radiation with a wavelength of 0.71073 Å, using an exposure time of 20 s/frame. A total of 1568 frames were collected with a frame width of 0.5° with 99.7% completeness accomplished. Phi and omega scans are reported at 100 K. All cell refinements as well as data reductions were performed using SAINT-Plus software.^82^ Absorption corrections were made using the multiscan technique and the SADABS software package. All structures were solved with the SIR-97 software package,^83^ and structure refinement was made using SHELXL-2013^84^ and WinGX.^85^ The molecular graphics were drawn using DIAMOND.^86^ All non-hydrogen atoms were refined anisotropically. Methyl and aromatic hydrogen atoms were placed in geometrically idealized positions and constrained to ride on their parent atoms, with C–H = 0.98 and 0.95 A° and U_iso_(H) = 1.5Ueq(C) and 1.2Ueq(C), respectively. The NH hydrogen atoms were located in the difference Fourier map and freely refined with U_iso_(H) = 1.5Ueq(N). The Fc versus Fo plot proved five reflections to be outliers, and they were removed from the refinement as systematic errors.

### Synthesis

The starting synthon *fac*-[NEt_4_]_2_[Re(CO)_3_(Br)_3_] (ReAA) and the ligands (2,6-dimethoxypyridyl)imidazo[4,5-*f*]1,10-phenanthroline (**L1**), (indole)imidazo[4,5-*f*]1,10-phenanthroline (**L2**), (5-methoxyindole)imidazo[4,5-*f*]1,10-phenanthroline (**L3**), (biphenyl)imidazo[4,5-*f*]1,10-phenanthroline (**L4**), (fluorene)imidazo[4,5-*f*]1,10-phenanthroline (**L5**), (benzo[*b*]thiophene)imidazo[4,5-*f*]1,10-phenanthroline (**L6**), (5-bromothiazole)imidazo[4,5-*f*]1,10-phenanthroline (**L7**), and (4,5-dimethylthiophene)imidazo[4,5-*f*]1,10-phenanthroline (**L8**) have been synthesized and characterized as reported previously in the literature.^45,50^^13^C{^1^H} NMR was attempted for **1**–**8**; however, after 18,000 scans on highly concentrated NMR samples, poor or no peaks were obtained. Due to economic implications, other characterization methods were relied upon.

### General Synthetic Procedure for Re(I) Tricarbonyl Complexes

ReAA was added to 3 equiv of AgNO_3_ solution in 10 mL of water (at pH 2) and stirred at room temperature for 24 h, which resulted in a gray/white precipitate of AgBr. The precipitate was filtered, and 1.1 equiv of the ligand was added to the filtrate. The solution was stirred at 80 °C for 12 h, and a solid was separated from the solution. The reaction mixture was filtered and washed three times with distilled water to yield a solid product.

### *fac*-[Re(CO)_3_(**L1**)(H_2_O)][NO_3_] (**1**)

0.206 g (0.267 mmol) ReAA, 0.136 g (0.801 mmol) AgNO_3_, 0.099 g (0.294 mmol) **L1**. Yield: 0.0902 g (0.131 mmol, 49%); elemental analysis for C_23_H_17_N_6_O_9_Re: calculated: C = 39.04%, N = 11.88%, H = 2.42%; found: C = 38.91%, N = 11.90%, H = 2.44%.; IR (ATR, cm^–1^): ν_N-H_ = 3346, ν_O-H_ = 3064, 2958, ν_C=O_ = 2028, 1896, ν_C=N_ = 1605, ν_C=N_ = 1582, 1452, ν_C-O_ = 1323, 1274.; ^1^H NMR (400 MHz, DMSO*-d*_6_): δ 9.5 (4H, m), 8.6 (1H, d, *J* = 8.2 Hz), 8.2 (2H, bs), 6.7 (1H, d, *J* = 8.2 Hz), 4.2 (3H, s), 4.0 (3H, s) ppm; ^13^C{^1^H} NMR (151 MHz, DMSO*-d*_6_): δ 196.9, 164.2, 163.9, 159.8, 159.6, 150.0, 142.5, 142.3, 124.6, 105.1, 104.6, 103.2, 102.9, 54.6, 54.5, 54.4, 54.3 ppm. UV/Vis (DMSO): λ_max_ = 350 nm, ε = 12,223 M^–1^ cm^–1^. ESI-MS (CH_3_OH, +ve ion mode): *m*/*z* 627.9 ([M - NO_3_^–^ - H_2_O]^+^); calcd, 627.6).

### *fac*-[Re(CO)_3_(**L2**)(H_2_O)][NO_3_] (**2**)

0.201 g (0.260 mmol) ReAA, 0.132 g (0.777 mmol) AgNO_3_, 0.096 g (0.286 mmol) **L2**. Yield: 0.098 g (0.143 mmol, 55%); elemental analysis for C_24_H_15_N_6_O_7_Re: calculated: C = 42.04%, N = 12.26%, H = 2.21%; found: C = 41.94%, N = 12.31%, H = 2.19%; IR (ATR, cm^–1^): ν_N-H_ = 3387, ν_O-H_ = 3074, ν_C=O_ = 2019, 1912, 1886, ν_C=N_ = 1576, ν_C=N_ = 1318; ^1^H NMR (400 MHz, DMSO*-d*_6_): δ 9.4 (4H, overlying multiplet, singlet, and doublet), 8.7 (1H, s), 8.3 (2H, overlying singlets), 8.1 (1H, s), 7.6 (1H, s), 7.3 (2H, s) ppm; ^13^C{^1^H} NMR (151 MHz, DMSO*-d*_6_): δ 197.0, 143.6, 137.0, 134.3, 127.2, 127.1, 125.5, 123.1, 121.8, 121.1, 112.7 ppm; UV/Vis (DMSO): λ_max_ = 345 nm, ε = 23,989 M^–1^ cm^–1^. ESI-MS (CH_3_OH, +ve ion mode): *m*/*z* 606.2 ([M - NO_3_^–^ - H_2_O]^+^); calcd, 605.6).

### *fac*-[Re(CO)_3_(**L3**)(H_2_O)][NO_3_] (**3**)

0.20 g (0.260 mmol) ReAA, 0.132 g (0.780 mmol) AgNO_3_, 0.104 g (0.286 mmol) **L3**. Yield: 0.0614 g (0.0858 mmol, 33%); elemental analysis for C_25_H_17_N_6_O_8_Re: calculated: C = 41.96%, N = 11.74%, H = 2.39%; found: C = 41.86%, N = 11.72%, H = 2.38%; IR (ATR, cm^–1^): ν_N-H_ = 3121, ν_O-H_ = 2921, 2852, ν_C=O_ = 2028, 1936, 1910, ν_C=N_ = 1603, ν_C=N_ = 1319, ν_C-O_ = 1217. ^1^H NMR (600 MHz, DMSO*-d*_6_): δ 11.7 (1H, s), 9.3 (2H, broad singlet), 9.2 (2H, singlet), 8.2 (2H, broad singlet), 8.1 (2H, s), 7.5 (1H, d, *J* = 8.7 Hz), 6.9 (1H, d, *J* = 8.6 Hz), 3.9 (3H, s) ppm. ^13^C{^1^H} NMR (151 MHz, DMSO*-d*_6_): δ 155.1, 132.1, 126.1, 125.7, 113.3, 113.0, 103.6, 55.9 ppm. UV/Vis (DMSO): λ_max_ = 390 nm, ε = 19,145 M^–1^ cm^–1^; ESI-MS (CH_3_OH, +ve ion mode): *m*/*z* 636.2 ([M - NO_3_^–^ - H_2_O]^+^); calcd, 635.6).

### *fac*-[Re(CO)_3_(**L4**)(H_2_O)][NO_3_] (**4**)

0.102 g (0.132 mmol) ReAA, 0.067 g (0.397 mmol) AgNO_3_, 0.054 g (0.145 mmol) **L4**. Yield: 0.0219 g (0.0303 mmol, 23%); elemental analysis for C_28_H_18_N_5_O_7_Re: calculated: C = 46.53%, N = 9.69%, H = 2.51%; found: C = 46.23%, N = 9.71%, H = 2.48%. IR (ATR, cm^–1^): ν_O-H_ = 3091, 3027, ν_C=O_ = 2038, 1993, 1956, ν_C=C_ = 1808, 1775, 1610, ν_C=N_ = 1554, ν_C=N_ = 1339. ^1^H NMR (600 MHz, DMSO*-d*_6_): δ 13.9 (1H, s), 9.1 (4H, s), 8.3 (2H, d, *J* = 7.3 Hz), 8.0 (2H, broad singlet), 7.9 (2H, d, *J* = 7.4 Hz), 7.8 (2H, d, *J* = 7.0 Hz), 7.5 (2H, t, *J* = 13.8, 6.9 Hz), 7.4 (1H, t, *J* = 13.8, 6.9 Hz) ppm. ^13^C{^1^H} NMR (151 MHz, DMSO*-d*_6_): δ 151.6, 141.9, 139.6, 129.5, 129.0, 128.5, 127.7, 127.4, 127.2, 125.1 ppm. UV/Vis (DMSO): λ_max_ = 337 nm, ε = 25,793 M^–1^ cm^–1^. ESI-MS (CH_3_OH, +ve ion mode): *m*/*z* 643.2 ([M - NO_3_^–^ - H_2_O]^+^); calcd, 642.6).

### *fac*-[Re(CO)_3_(**L5**)(H_2_O)][NO_3_] (**5**)

0.10 g (0.130 mmol) ReAA, 0.066 g (0.39 mmol) AgNO_3_, 0.055 g (0.143 mmol) **L5**. Yield: 0.0343 g (0.0469 mmol, 36%); elemental analysis for C_29_H_18_N_5_O_7_Re: calculated: C = 47.41%, N = 9.53%, H = 2.47%; found: C = 47.44%, N = 9.50%, H = 2.43%. IR (ATR, cm^–1^): ν_N-H_ = 3471, ν_O-H_ = 3057, 2902, ν_C=O_ = 2028, 1926, 1905, ν_C=N_ = 1610, ν_C=C_ = 1557, 1476, ν_C-H_ = 1446, ν_C=N_ = 1317. ^1^H NMR (600 MHz, DMSO*-d*_6_): δ 9.1 (4H, merged singlet), 8.3 (1H, s), 8.2 (1H, d, *J* = 7.6 Hz), 8.0 (3H, merged singlet), 7.9 (1H, d, *J* = 4.6 Hz), 7.6 (1H, d, *J* = 7.3 Hz), 7.3 (2H, dt, *J* = 29.2, 7.3 Hz), 4.0 (2H, s) ppm. ^13^C{^1^H} NMR (151 MHz, DMSO*-d*_6_): δ 152.2, 146.7, 144.1, 144.0, 143.1, 140.7, 139.2, 128.2, 127.8, 127.4, 125.7, 125.6, 125.1, 123.2, 120.9, 120.8, 36.8 ppm. UV/Vis (DMSO): λ_max_ = 342 nm, ε = 29,853 M^–1^ cm^–1^. ESI-MS (CH_3_OH, +ve ion mode): *m*/*z* 655.2 ([M - NO_3_^–^ - H_2_O]^+^); calcd, 654.7).

### *fac*-[Re(CO)_3_(**L6**)(H_2_O)][NO_3_] (**6**)

0.199 g (0.258 mmol) ReAA, 0.131 g (0.774 mmol) AgNO_3_, 0.10 g (0.283 mmol) **L6**. Yield: 0.105 g (0.149 mmol, 58%); elemental analysis for C_24_H_14_N_5_O_7_SRe: calculated: C = 41.02%, N = 9.97%, S = 4.56%, H = 2.01%; found: C = 41.20%, N = 9.96%, S = 4.60%, H = 2.02%. IR (ATR, cm^–1^): ν_N-H_ = 3397, ν_O-H_ = 3075, ν_C=O_ = 2028, 1920, 1894, ν_C=N_ = 1660, ν_C=C_ = 1581, 1562, ν_C=N_ = 1370, 1304. ^1^H NMR (400 MHz, DMSO*-d*_6_): δ 9.5 (1H, dd, *J* = 15.3, 4.7 Hz), 9.3 (1H, broad singlet), 9.1 (2H, overlapping singlets), 8.3 (2H, overlapping singlets), 8.0 (3H, m), 7.5 (2H, overlapping signals) ppm. ^13^C{^1^H} NMR (101 MHz, DMSO*-d*_6_): δ 196.9, 152.8, 148.6, 140.2, 140.0, 139.9, 133.2, 132.8, 127.6, 126.6, 126.2, 125.7, 125.5, 125.3, 125.2, 124.9, 124.2, 123.4, 123.2, 123.0 ppm. UV/Vis (DMSO): λ_max_ = 369 nm, ε = 28,040 M^–1^ cm^–1^. ESI-MS (CH_3_OH, +ve ion mode): *m*/*z* 623.1 ([M - NO_3_^–^ - H_2_O]^+^); calcd, 622.6).

### *fac*-[Re(CO)_3_(**L7**)(H_2_O)][NO_3_] (**7**)

0.206 g (0.267 mmol) ReAA, 0.136 g (0.801 mmol) AgNO_3_, 0.112 g (0.294 mmol) **L7**. Yield: 0.0254 g (0.0347 mmol, 13%); elemental analysis for C_19_H_10_N_6_O_7_SBrRe: calculated: C = 31.15%, N = 11.47%, S = 4.37%, Br = 10.91%, H = 1.37%; found: C = 31.25%, N = 11.51%, S = 4.41%, Br = 10.89%, H = 1.35%. IR (ATR, cm^–1^): ν_N-H_ = 3398, ν_O-H_ = 3059, ν_C=O_ = 2025, 1918, 1893, ν_C=N_ = 1608, ν_C=N_ = 1312, 1254, ν_C-Br_ = 805, ν_C-S_ = 805. ^1^H NMR (400 MHz, DMSO*-d*_6_): δ 13.9 (1H, s), 11.7 (1H, s), 9.5 (2H, s), 8.2 (3H, overlying singlets), 7.5 (1H, triplet, *J* = 16.9, 8.72), 6.9 (1H, doublet, *J* = 8.7 Hz) ppm. ^13^C{^1^H} NMR (101 MHz, DMSO*-d*_6_): δ 197.0, 159.0, 152.4, 145.3, 140.0, 128.1, 124.5 ppm. UV/Vis (DMSO): λ_max_ = 261 nm, ε = 1494 M^–1^ cm^–1^. ESI-MS (CH_3_OH, +ve ion mode): *m*/*z* 671.1 ([M - NO_3_^–^]^+^); calcd, 670.5).

### *fac*-[Re(CO)_3_(**L8**)(H_2_O)][NO_3_] (**8**)

0.203 g (0.264 mmol) ReAA, 0.135 g (0.792 mmol) AgNO_3_, 0.112 g (0.294 mmol) **L8**. Yield: 0.0609 g (0.0896 mmol, 34%); elemental analysis for C_22_H_16_N_5_O_7_SRe: calculated: C = 38.82%, N = 10.29%, S = 4.71%, H = 2.37%; found: C = 38.85%, N = 10.27%, S = 4.69%, H = 2.38%. IR (ATR, cm^–1^): ν_N-H_ = 3066, ν_O-H_ = 2923, 2865, ν_C=O_ = 2025, 1904, 1894, ν_C=N_ = 1617, ν_C=C_ = 1586, 1545, ν_C-S_ = 806. ^1^H NMR (400 MHz, DMSO*-d*_6_): δ 14.1 (1H, s), 9.4 (2H, d, *J* = 4.9 Hz), 9.1 (2H, broad multiplet), 8.1 (2H, broad doublet, *J* = 19.8 Hz), 7.6 (1H, s), 2.4 (3H, s), 2.2 (3H, s) ppm. ^13^C{^1^H} NMR (101 MHz, DMSO*-d*_6_): δ 197.9, 190.0, 151.7, 148.7, 137.0, 134.8, 133.2, 132.9, 130.3, 127.8, 126.9, 13.8, 13.5 ppm. UV/Vis (DMSO): λ_max_ = 341 nm, ε = 6340 M^–1^ cm^–1^. ESI-MS (CH_3_OH, +ve ion mode): *m*/*z* 601.1 ([M - NO_3_^–^ - H_2_O]^+^); calcd, 600.6).

### Stability Study

The stability of the complexes in DMSO, aqueous buffer, and FBS solutions was monitored using time-dependent UV/Vis absorption measurements. Briefly, the concentration of each complex was maintained at 10 μM except for complex **7**. In fact, due to poor solubility, we used 5 μM concentration for compound **7**. A 1:20 *v/v* DMSO:10 mM phosphate buffer pH 7.4 in the presence of 4 mM NaCl solution of the complexes was used to evaluate the aqueous solution stability. In the case of the stability in FBS solution, we prepared the complex solution in 1:99 v/v DMSO:10 mM phosphate buffer pH 7.4 in the presence of 10% FBS. The stability of the complexes in solution was monitored up to 6 h.

### Determination of Acid Dissociation Constant (p*K*_a_)

pH measurements were performed using a Hanna pH211 Microprocessor pH meter, equipped with a HI 1131 probe, which was calibrated using standard buffer solutions, with pH = 4.01, 7.00, and 10.00. A Varian Cary 50 Conc UV/Vis spectrophotometer coupled to a personal computer capable of performing least squares analyses on the absorption values *vs* pH data was utilized for the kinetic measurements. Microsoft Office Excel (2019) and Scientist Micromath, version 2.01 were used to fit the collected data to specific functions.^87,88^ A circulating water bath system was used to control and maintain the temperature at 25 °C. The ionic strength (μ) of all solutions was maintained at 1 M NaClO_4_.

The stability of complexes in DMSO solution was also monitored by ESI-MS measurement. Briefly, 10 μM DMSO solution of the complexes was incubated for 48 h at 37 °C. Finally, 10 μL of DMSO solution was diluted in 1 mL of methanol and analyzed by ESI-MS.

### Distribution Coefficient

The distribution coefficient or lipophilicity (log *D*) was determined using a standard shake flask technique.^58^ Briefly, 40 μL of 1 mM DMSO solution of complex was added to 4 mL of pre-equilibrated 10 mM phosphate buffer (pH 7.4) octanol mixture (1:1 *v/v*) and agitated continuously in a vertical shaker for 6 h. Then, each layer of the mixture was separated by centrifugation and transferred to new vials. Further, the complex concentration in each layer was determined by measuring their absorption spectra. Finally, the lipophilicity was determined using eq 3 below.
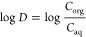
3

### BSA Binding Study

The BSA binding study was performed by measuring the internal fluorescence quenching of BSA. BSA has an intrinsic fluorescence emission maxima at 341 nm upon excitation at 280 nm.^66,67^ Briefly, 20 μM BSA solution in 1× Tris buffer (pH 7.4) was incubated with different concentrations (0–10 equiv) of complex solution for 15 min at 37 °C. Further, their fluorescence intensities were recorded at 341 nm upon excitation at 280 nm. Finally, the fluorescence quenching constant and the binding constant were calculated using the Stern–Volmer equation (eq 4) and the Scatchard equation (eq 5), respectively.
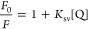
4
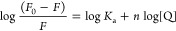
5

In the equations, the fluorescence emission intensity of native protein is denoted as *F*_0_, the fluorescence emission intensity in the presence of quencher is denoted as *F*, [Q] corresponds to the concentration of quencher for *F*, *K*_sv_ is the Stern–Volmer quenching constant, *K*_a_ is denoted as the binding constant of quencher and protein, and *n* is the number of binding sites per protein molecule. Complexes showed no emission at 341 nm when excited at 280 nm.

### DNA Binding Study

#### With Model Nucleobase

The DNA binding ability of complex **6** through covalent bond formation was studied in the presence of a model nucleobase, guanosine, using ^1^H NMR and ESI-MS. Precisely, 1.5 mM DMF-*d*_7_ solution of complex **6** was allowed to react with 3 mM guanosine solution in DMF-*d*_7_ at 37 °C and the binding was monitored at different intervals of time by recording ^1^H NMR spectra up to 24 h. Finally, after 24 h, 5 μL of DMF solution was diluted in 1 mL of methanol and analyzed by ESI-MS.

#### Preparation of ctDNA Solution

Calf thymus deoxyribonucleic acid (ctDNA) and tris–HCl buffer solution (pH = 7.4, 1 M) were purchased from Sigma Aldrich, South Africa, and used without further purification. The ctDNA stock solution was prepared by stirring a solution of ctDNA in Tris–HCl buffer for 12 h at room temperature. The purity of the stock solution was confirmed by the ratio of *A*_260nm_/*A*_280nm_ (1.843), indicating that the solution was free of protein.^45,81,89,91^ This solution was stored at 4 °C between scans and discarded 2 days after preparation. The molar extinction coefficient value of ctDNA at 260 nm (6600 L mol^–1^ cm^–1^) was employed in the determination of the stock solution concentration.

#### Ethidium Bromide Displacement Assay

To check the possibility of DNA intercalation due to the presence of a planer fused ring ligand system, we performed the standard ethidium bromide (EB) displacement assay. Precisely, 10 μM ctDNA solution in 1× Tris·NaCl buffer was incubated at 37 °C with 3 equiv of EB for 30 min. After this, 0 to 10 equiv of complex **6** were added to the solution and incubated for an additional 30 min at 37 °C. Each solution was excited at 540 nm, and emission was measured from 550 to 800 nm. Finally, the apparent binding constant (*K*_app_) was measured using eq 6.
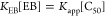
6where [EB] is the concentration of EB (30.0 μM) and [C_50_] is the concentration of complex when the reduction of fluorescence reach 50%. *K*_EB_ is the binding constant of EB (*K*_EB_ = 1 × 10^7^ M^–1^ for ctDNA).^90^ The IC_50_ values were calculated from plots of normalized fluorescence intensity versus complex concentration (Figure S24B).

#### DNA Melting

The DNA binding ability of complex **6** was confirmed by measuring the DNA melting temperature prior to complex binding. Briefly, 40 μM ctDNA was incubated with 30 μM of the complex at 37 °C for 15 min. Afterward, the absorbance of the solution was measured at 260 nm in each °C increment in temperature within the range from 60–97 °C. A Cary UV 300 (Agilent Technologies) was used to measure all absorbances. The experiment was programmed with a 1 °C/min temperature ramp rate from the temperature range 60–97 °C, and the holding time was 1 min. Finally, the absorbance of each experiment was normalized to 0 and 1 and further compared with the melting temperature of native ctDNA.

### Cell Lines and Cell Culture

Human prostate adenocarcinoma (PC3) and normal human retinal pigment epithelial-1 (RPE-1) cells were originally procured from the American Type Culture Collection (ATCC). The PC3 and RPE-1 cells were maintained in Ham’s F-12K (Kaighn’s) Medium and DMEM/F-12, GlutaMAX Supplement medium containing 10% fetal bovine serum (GIBCO) and 1% penicillin–streptomycin (100 units/mL, GIBCO), respectively.

### Cytotoxicity

The standard Resazurin assay was performed to determine the cytotoxicity of the synthesized complexes. In particular, 4 × 10^3^ cells were seeded in each well of a 96 well microplate and incubated at 37 °C under normoxic conditions (∼15% O_2_ level). After 24 h of the initial incubation period, the medium was replenished by a fresh metal complex-containing medium (100 μL/well) and incubated for an additional 48 h. Initially, the metal complex was dissolved in DMSO and the target concentration was achieved by further dilution with the medium. A total of seven or nine different concentrations of the metal complex solutions from 10 nM to 100 μM were used to observe the dose-dependent cytotoxicity. The ultimate concentration of DMSO in each well was ≤1%. Finally, the metal complex-containing medium was carefully aspirated and an equal volume of Resazurin (0.2 mg/mL)-containing medium was added. After 4 h of incubation at 37 °C, the fluorescence of the produced resorufin product (λ_ex_ = 540 nm, λ_em_ = 590 nm) was measured in either a Tecan infinite F200PRO Microplate Reader or a Cytation 5, Cell Imaging Multi-Mode Reader, BioTek. Finally, the half-maximal inhibitory concentration (IC_50_) value was calculated using the GraphPad Prism 5 software from the plot of % of cell viability *vs* log[complex concentration] after applying nonlinear fitting. Each of the reported IC_50_ values is the mean of three individual experiments with standard deviation, and each concentration was assayed in sextuplicate in each successive experiment.

The cytotoxic inactivation of most selective complexes **3** and **6** in hypoxic conditions (2.0% O_2_) was also measured against PC3 cells. A similar protocol as stated in normoxia cytotoxicity was followed to determine their IC_50_ in hypoxic conditions, keeping everything unaltered except the incubation in the presence of 2.0% O_2_. Initially, at least three consecutive cell passages were maintained in hypoxic conditions before starting any experiments.

### Cytotoxicity against 3D Cell Spheroid

1 × 10^4^ PC3 cells were seeded in each well of a Corning 96 Well Ultra-Low Attachment Treated Spheroid Microplate. After 96 h of incubation at 37 °C in normoxic conditions, the average spheroid size observed was 435 μm. Half of the medium was replaced by an equal volume of complex-containing (two times of target concentrations) medium. After 72 h, the cell viability was measured using the AlamarBlue assay. Finally, the IC_50_ values were determined by using GraphPad Prism 5 software after applying four parameter fitting.

### Intracellular Accumulation Study

The fluorescence property is highly dependent on pH and the cellular microenvironment.^74,91^ Hence, we quantified Re in different cellular organelles using ICP-MS to determine the complex localization in cells.

An adequate number of PC3 cells were seeded in a 150 mm diameter Petri dish using F12K medium and incubated in normoxic conditions at 37 °C. The medium from each Petri dish was replenished every 2 days. When the cell confluency reached around 80–90%, a fresh medium containing 5 μM complexes **3** or **6** was added and incubated for an additional 1.5 h in normoxic conditions at 37 °C. Cells were harvested by trypsinization. The medium was removed by centrifugation, and the cell pellet was washed twice with cold DPBS. The nucleus and mitochondria of treated cells were then isolated using the standard kit protocols mentioned below. To determine the total rhenium uptake, 5 × 10^5^ cells were collected and digested using 100 μL of 70% nitric acid at 60 °C for 12 h. The acid solution was further diluted using 4900 μL of MilliQ water. Finally, the rhenium concentration was measured using ICP-MS. Each experiment was performed in triplicate.

Nuclei: The nuclei isolation kit, Nuclei EZ Prep (NUC101) (Sigma-Aldrich) was used to isolate the nuclei from the complex-treated cells, and the standard kit protocol was employed for the isolation process. The complex-treated cells were exposed to 4 mL of ice-cold Nuclei EZ lysis buffer and vortexed briefly to lyse the cells. The solution was settled at 4 °C for another 5 min and centrifuged at 500*g* for 5 min at 4 °C. The supernatant was removed carefully. The cell and nucleus pellets were redissolved using ice-cold Nuclei EZ lysis buffer, and the same process was repeated to maximize the nuclei yield. During this isolation step, both the nucleus and cells were stained with the trypan blue dye prior to counting using a hemocytometer to obtain the exact number of the nuclei and intact cells.

Mitochondria: The mitochondria isolation kit (MITOISO2) (Sigma-Aldrich) was used to isolate the mitochondria from the complex-treated cells, and the standard kit protocol was employed for the isolation procedures. By following the kit protocol, the complex-treated cells were lysed using 1× extraction buffer at 4 °C using a Dounce homogenizer. The exact number of lysed cells was counted using a hemocytometer after trypan blue staining. The intact cells and other heavier cell fragments were removed from the solution by centrifugation at 600*g* for 10 min at 4 °C. The supernatant was transferred carefully and centrifuged at 11000*g* for 10 min at 4 °C to isolate the mitochondria. Hence, the total amount of isolated mitochondria corresponds to the total lysed cells. The percentage of rhenium accumulation in the nucleus and mitochondria was calculated using the total accumulated rhenium corresponding to 5 × 10^5^ cells.

### Sample Preparation for ICP-MS

The samples were digested in either 100 μL (cell pellet) or 500 μL (nucleus and mitochondria pellet) of 70% HNO_3_ at 60 °C for 12 h. The acid solution was further diluted (1:50 *v/v*) with MilliQ water. Finally, the rhenium concentration in the solution was analyzed using a high-resolution ICP-MS instrument of ThermoScientific (Element II, ThermoScientific) at the Institut de Physique du Globe de Paris, France.

### Cellular Uptake Pathways

1.5 × 10^6^ PC3 cells were seeded in a 6 cm Petri dish and incubated in normoxic conditions at 37 °C. After 24 h of seeding, the cells were treated with different cellular uptake pathways inhibitors for 2 h in normoxic conditions. The inhibitor used here were as follows: 1 mM tetraethylammonium chloride (cationic transporter inhibitor), 5 μM oligomycin and 50 mM 2-deoxy-d-glucose (metabolic inhibitors), and 50 mM ammonium chloride (endocytotic inhibitor) as different uptake pathway inhibitors. Additionally, we pre-incubated the cells at 4 °C for 2 h to determine the involvement of passive transport on the cellular uptake process. After 2 h, cells were washed twice with DPBS and 5 μM of complex (**3** and **6**) solution was added. After 2 h of incubation in normoxic conditions, cells were harvested using trypsinization and counted. 1 × 10^6^ cells were separated and digested in 100 μL of conc. HNO_3_. Finally, the acid solution was diluted by addition of 4900 μL of MQ water to keep the acid % within 2. The rhenium concentration in solution was measured by ICP-MS. Each successive experiment and control experiments were performed three times to determine the average value with standard deviation.

### Mito Stress Test Using Seahorse Apparatus

3 × 10^4^ PC3 cells/well were seeded of a Seahorse XF Cell Culture Microplate using 80 μL of FBS-supplemented F12K medium and incubated in normoxic conditions for 24 h at 37 °C. The medium was replaced by an equal volume of compound (**3**, 0.4 and 5 μM; **6**, 0.05 and 2 μM; and cisplatin, 7 and 30 μM)-containing medium. After 24 h of treatment, media were removed, and the treated cells were washed very carefully with the Seahorse XF medium three times. Finally, the cells were incubated at 37 °C for 1 h in a non-CO_2_ incubator. The Mito Stress assay was run in a Seahorse Xfe96 instrument (Agilent) at 37 °C using multiple inhibitors, viz., ATP synthase inhibitor (oligomycin, 1 μM), proton gradient and mitochondrial membrane potential collapsing agent (FCCP, 1 μM), and mitochondrial respiratory complex I and III inhibitors (rotenone, 1 μM and antimycin A, 1 μM, respectively). After the experiment, the cells were fixed using 4% *p*-formaldehyde solution and the nucleus was stained with DAPI. Each well was imaged in a Cytation 5 Cell Imaging Multimode Reader (BioTek) using a 10× objective lens. Finally, the cells from each image were calculated by using Gen5 software (Figure S35) and by utilizing the cell count and the data was normalized against the same cell number.

### Cell Death Mechanism

To evaluate the possible cell death pathways, we measured the cell viability in the presence of different cell death pathway inhibitors.^44,75^ Briefly, 1 × 10^3^ PC3 cells/well were seeded in 96-well plates and incubated for 24 h in normoxic conditions at 37 °C. The medium was removed and treated with inhibitors for 1 h. Autophagy inhibitor (3-methyladenine (100 μM)), apoptosis inhibitor (Z-VAD-FMK (20 μM)), paraptosis inhibitor (cycloheximide (0.1 μM)), and necrosis inhibitor (necrostatin-1 (60 μM)) were the different inhibitors used. After 1 h, the inhibitor-containing medium was removed and washed with 1× DPBS. Then, different concentrations of complex solution were added and incubated for 36 h in normoxic conditions. Finally, the cell viability was determined by Resazurin assay (check the Cytotoxicity section for details) to evaluate the IC_50_ values.

### Annexin-V/PI Assay for Apoptosis Detection

1.5 × 10^6^ PC3 cells were seeded in a 6 cm Petri dish using a F12K medium and incubated at 37 °C in normoxic conditions. After 24 h of incubation, the medium was replenished with a complex-containing medium and incubated additionally for 24 h. Two different concentrations of complexes **3** and **6** were used to determine the possible apoptosis pathways for cell killing. The cells were then harvested by trypsinization and washed twice with DPBS. The cells were further stained with both FITC-tagged Annexin-V (cat. no. 556419, BD Biosciences) and propidium iodide (cat. no. 51-66211E, BD Biosciences). Finally, the stained cells were analyzed using a BD LSR II flow cytometry instrument (BD Biosciences) at Institut Curie, Paris, France.

### ROS Analysis

1.5 × 10^4^ PC3 cells were seeded in the dark in 96-well plates for 24 h. Four different concentrations of complexes **3** and **6** were used to treat the cells for 8 h, and then two different concentrations of H_2_O_2_ were used to treat the cells for 30 min (10 and 15 μM). The medium was removed, and 40 μM DCFH-DiOxy-Q (Kit No. ab238535, Abcam) in 10% FBS containing HEPES was added in each well followed by incubation at 37 °C for 30 min. The cells were washed twice with HEPES, and finally fluorescence intensity was measured (λ_ex_/λ_em_; 485/530 nm) using a Cytation 5 Cell Imaging Multimode Reader (BioTek). Then, the cells were fixed using *p*-formaldehyde and stained using DAPI. The cells were finally counted in the same way as stated before and utilized to normalize the data.

## Supporting Information Available

Crystallographic data (Tables S1–S3) and interactions (Figures S1–S6), stability studies (Figures S7–S14), p*K*_a_ determination (Figures S15–S18), DNA binding studies (Figures S19–S24), BSA binding studies (Figures S25–S28), cytotoxicity (Table S4 and Figures S29–S33), mitochondrial respiration test (Figures S34–S37), ^1^H NMR spectra (Figures S38–S45), and FT-IR spectra of complexes (Figures S46–S53) (PDF)
