# Supplementary material for: Rhenium(I) Tricarbonyl Complexes of 1,10-Phenanthroline Derivatives with Unexpectedly High Cytotoxicity
Source: Inorg Chem. 2023 Jul 25;62(31):12237–51. doi: 10.1021/acs.inorgchem.3c00730 (PMC10410611; doi:10.1021/acs.inorgchem.3c00730)
Supplement: Supplementary file 1 — ic3c00730_si_001.pdf [file ic3c00730_si_001.pdf]

Supporting Information

Rhenium(I) Tricarbonyl Complexes of 1,10 Phenanthroline Derivatives with Unexpectedly High Cytotoxicity

Lucy E. Enslin,[a,#] *Kallol Purkait,*[b,#] *Maria Dalla Pozza,*[b] *Bruno Saubamea,*[c] *Pierre Mesdom,*[b] *Hendrik G. Visser,*[a*] *Gilles Gasser*[b*] *and Marietjie Schutte-Smith*[a*]

[a] University of the Free State, Department of Chemistry, Bloemfontein, 9301, South Africa.

[b] Chimie ParisTech, PSL University, CNRS, Institute of Chemistry for Life and Health Sciences, Laboratory for Inorganic Chemical Biology, F-75005 Paris, France.

[c] Université de Paris, Plateforme Imagerie Cellulaire et Moléculaire, F-75270 Paris, France

[#] these authors have contributed equally to the work.

* schuttem@ufs.ac.za

KEYWORDS: Bioorganometallic Chemistry; Cancer; Medicinal Inorganic Chemistry; Metals in Medicine; Rhenium.

Table of content

Crystallography……..……………………………………………………………………. S3

Stability study……………………………………………………………………….S11

Influence of H^+ ions – Acid dissociation constant determination ……………………...S20

DNA binding study ……………..………………………………………………………S22

BSA binding………………………………………………...……………………… S27

Cytotoxicity……………………………………………………….………………… S30

Mitochondrial respiration test…...…………………………………………………… S33

^{1}H NMR spectra of complexes…………………………………………………… S36

^{13}C NMR spectra of complexes……………………………………………………..S44

FT-IR spectra of complexes……………………………………………………………S52

ESI-MS spectra of complexes…………………………………………………………S56

Crystallography

Table S1 below presents a summary of the crystallographic data of *fac*-[Re(CO)$_3$(L3)(Br)].DMSO (**3a**).

Table S1. Crystallographic data of *fac*-[Re(CO)$_3$(L3)(Br)].DMSO (**3a**).

**Crystallographic data**			
Empirical formula	$C_{27}H_{21}BrN_5O_5ReS$	Formula weight	793.66
Crystal system	Triclinic	Space group	$P\overline{1}$
a (Å)	7.698(3)	α (°)	78.617(13)
b (Å)	13.414(5)	β (°)	79.509(13)
c (Å)	14.025(5)	γ (°)	75.493(13)
Volume (Å^3)	1360.9(9)	F (000)	768
Z	2	θ (°)	1.496 to 27.998
ρ_{calc} (mg/m^3)	1.937	Index ranges	-10<=h<=10, -17<=k<=17, -18<=l<=18
Crystal colour	Orange	Reflections collected	24068
Crystal morphology	Cuboid	Reflections with I>2θ(I)	6770
Crystal size (mm^3)	0.089 x 0.032 x 0.024	R_{int}	0.0671
μ (mm^{-1})	6.057	Completeness to 2θ (°, %)	25.242, 99.6
GooF	1.033	Data/restraints/ parameters	6544 / 1 / 369
R[I>2θ(I)]	R_1 = 0.0385, wR_2 = 0.0826	$\Delta\rho_{max}$, $\Delta\rho_{min}$ (e Å^{-3})	2.673 and -1.361
R all data	R_1 = 0.0508, wR_2 = 0.0882		

The dihedral angle, of 16.598(6) ° between the equatorial plane (O1-C23-Re1-C25-O3) and the plane through the imidazo[4,5-*f*]1,10-phenanthroline ligand (N1, N2, C1-C12) expresses the significant bend of the (methoxyindole)imidazo[4,5-*f*]1,10-phenanthroline ligand out of the equatorial plane towards the bromido ligand (Figure S1). Figure S2 illustrates the hydrogen bonding interactions, weak interactions, and the π-interactions observed while Figure S3 illustrates the one dimensional infinite chain observed in the structure of *fac*-[Re(CO)$_3$(L3)(Br)].DMSO (**3a**). The hydrogen bonding and π-interactions are summarised in Table S2 and Table S3 respectively.

Figure S1. Illustration of the dihedral angle between the plane through 5-(methoxyindole)imidazo[4,5-*f*]1,10-phenanthroline ((N1, N2, C1- C12) - blue plane) and the equatorial plane ((O1-C23-Re1-C25-O3) - green plane) in the structure of **3a**.

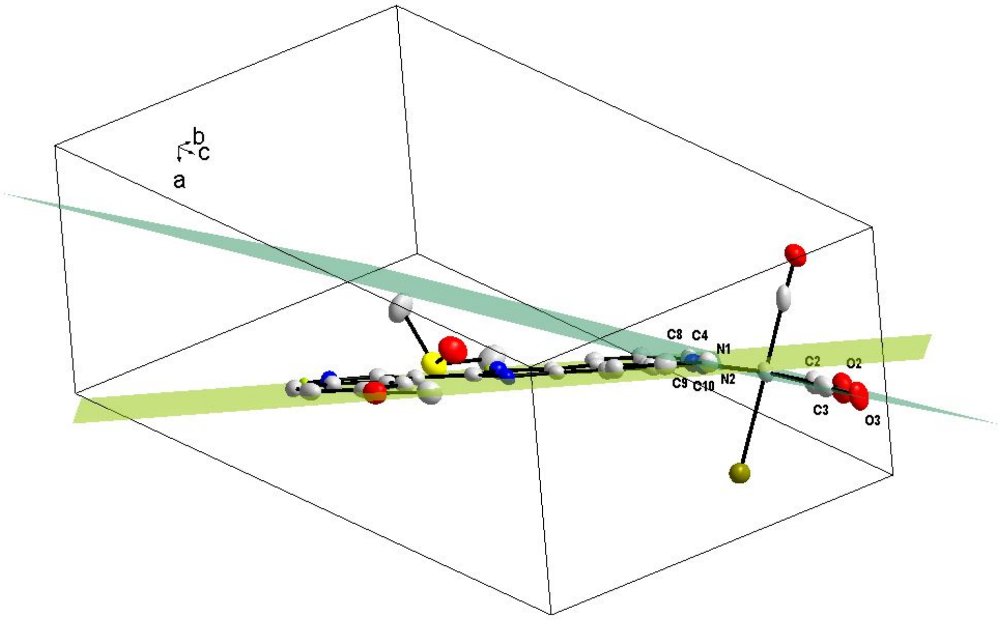

Figure S2. a) Hydrogen bonding and weak interactions (indicated in pink dashed lines) and b) π-interactions observed in the structure of **3a**.

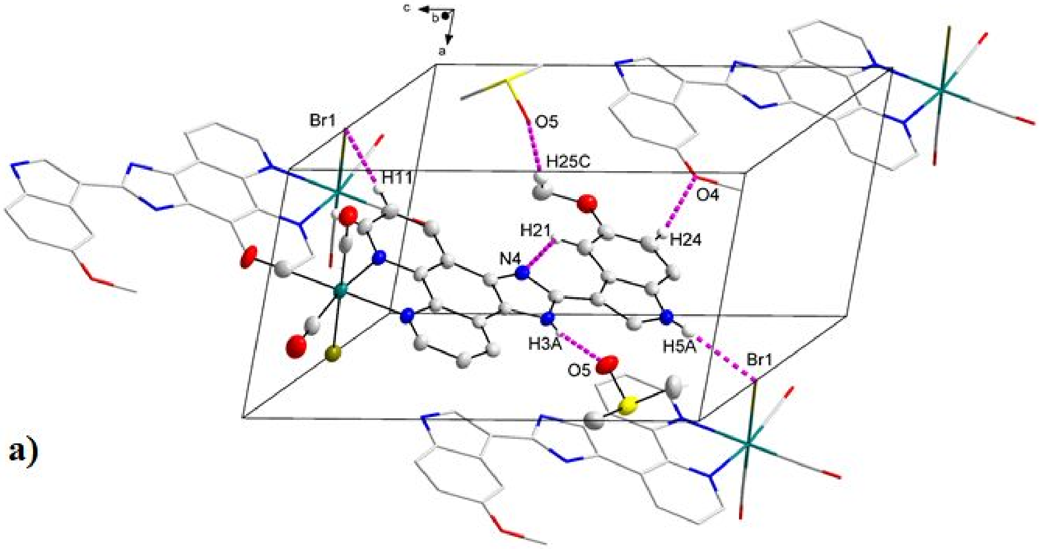

a)

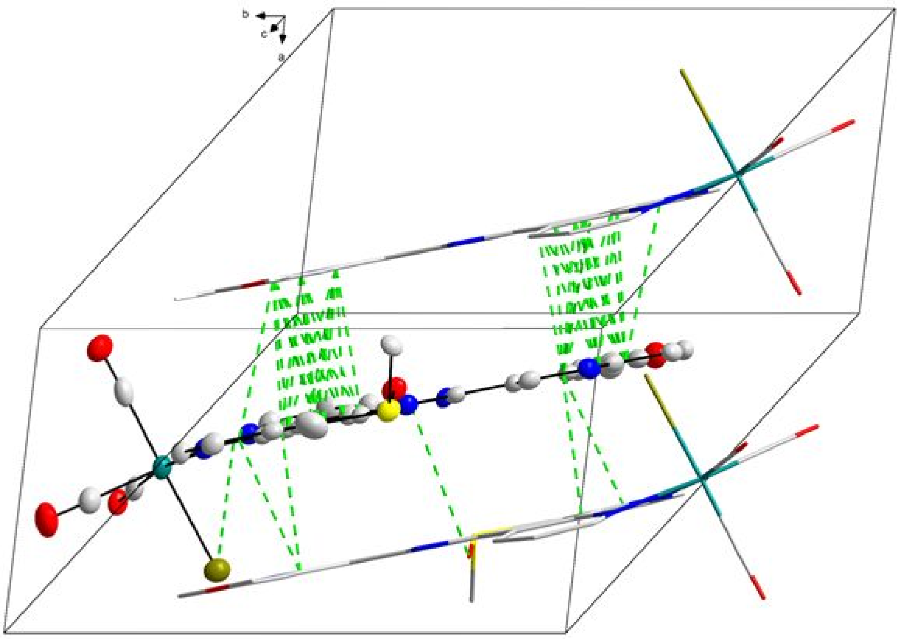

b)

Figure S3. Illustration of the one dimensional infinite chain along the c-axis observed in the structure of **3a**.

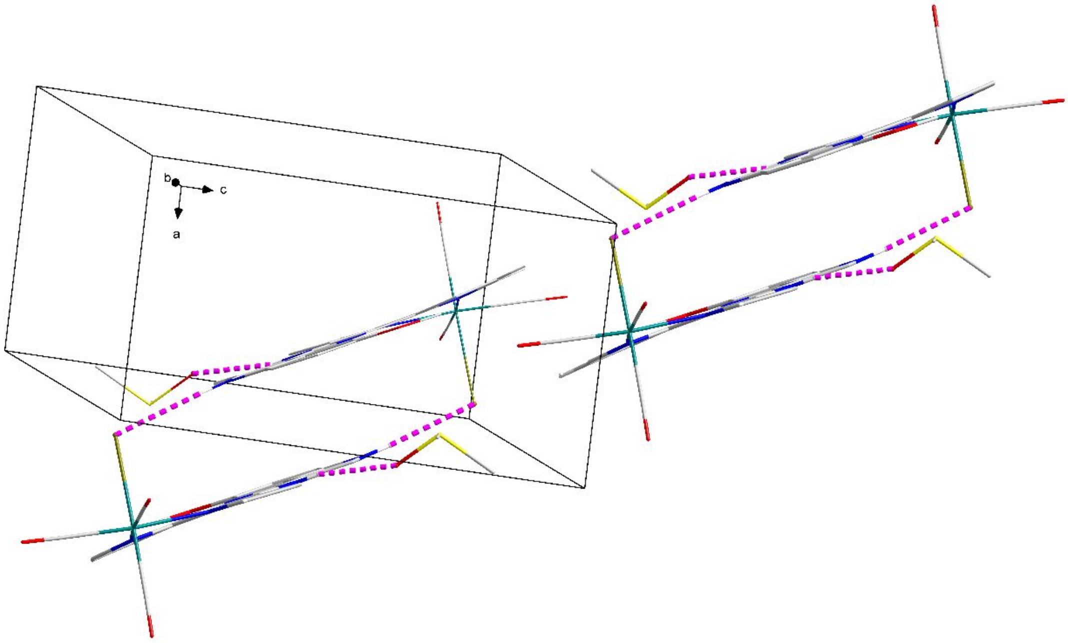

Table S2. Summary of the hydrogen bonding interactions, weak interactions, and π-interactions observed in the structure of **3a**.

D-H···A	d(D-H)	d(H···A)	d(D···A)	D-H···A (°)
N3-H3A···O5	0.93(5)	1.89(4)	2.805(6)	164(5)
N5-H5A···Br1[1]	0.81(7)	2.54(7)	3.342(4)	174(9)
C11-H11···Br1[2]	0.95	2.87	3.819(6)	173
C21-H21···N4	0.95	2.57	3.102(7)	116
C24-H24···O4[3]	0.95	2.48	3.379(7)	157
C25-H25C···O5[4]	0.98	2.56	3.506(8)	163
Y-X(I)	Res(I) → Cg(J)	X···Cg	Y-X···Cg	Y···Cg
Re1-Br1	[1] → Cg1	2.947(2)	38.44(4)	1.860(2)
S1-O5[1]	[1] → Cg2	3.728(5)	85.6(2)	3.910(3)

Symmetry codes and transformations used to generate equivalent atoms: [1] 2-x,1-y,1-z, [2] 1-x,1-y,2-z, [3] 1-x,-y,1-z, [4] 1-x,1-y,1-z. Cg1 = centroid of Re1, N1, C8, C9, N2, Cg2 = centroid of N3, C15, C14, N4, C16.

Table S3. Summary of the π-π-interactions observed in the structure of **3a.**

Cg···Cg	Cg···Cg distance (Å)	Cg···Cg	Cg···Cg distance (Å)
Cg3···Cg4[1]	3.502(3)	Cg9···Cg6[2]	3.329(3)
Cg3···Cg5[2]	3.782(3)	Cg9···Cg8[2]	3.416(3)

Cg3···Cg6[2]	3.553(3)	Cg9···Cg10[2]	3.567(3)
Cg3···Cg8[2]	3.528(3)	Cg9···Cg11[2]	3.509(3)
Cg3···Cg10[1]	3.748(3)	Cg9···Cg12[2]	3.419(3)
Cg3···Cg11[2]	3.461(3)	Cg9···Cg13[2]	3.377(3)
Cg3···Cg13[2]	3.312(3)	Cg9···Cg14[2]	3.445(3)
Cg3···Cg14[2]	3.757(3)	Cg9···Cg15[2]	3.317(3)
Cg4···Cg3[1]	3.502(3)	Cg10···Cg3[1]	3.748(3)
Cg4···Cg7[2]	3.711(3)	Cg10···Cg7[2]	3.373(3)
Cg5···Cg3[2]	3.782(4)	Cg10···Cg9[2]	3.567(3)
Cg6···Cg3[2]	3.553(3)	Cg11···Cg3[2]	3.461(3)
Cg6···Cg7[2]	3.451(3)	Cg11···Cg9[2]	3.508(3)
Cg6···Cg9[2]	3.329 (3)	Cg12···Cg7[2]	3.352(3)
Cg7···Cg4[2]	3.711(3)	Cg12···Cg9[2]	3.418(3)
Cg7···Cg6[2]	3.451(3)	Cg13···Cg3[2]	3.312(3)
Cg7···Cg8[2]	3.616(3)	Cg13···Cg7[2]	3.706(3)
Cg7···Cg10[2]	3.373(3)	Cg13···Cg9[2]	3.377(3)
Cg7···Cg12[2]	3.352(3)	Cg14···Cg3[2]	3.757(3)
Cg7···Cg13[2]	3.706(3)	Cg14···Cg7[2]	3.488(3)
Cg7···Cg14[2]	3.488(3)	Cg14···Cg9[2]	3.445(3)
Cg7···Cg15[2]	3.420(3)	Cg15···Cg3[2]	3.566(3)
Cg8···Cg3[2]	3.528(3)	Cg15···Cg7[2]	3.420(3)
Cg8···Cg7[2]	3.616(3)	Cg15···Cg9[2]	3.316(3)
Cg8···Cg9[2]	3.416(3)		

Symmetry codes and transformations used to generate equivalent atoms: [1] 2-x,1-y,1-z, [2] 1-x,1-y,1-z. Cg3 = centroid of N1,C17,C18,C19,C20, Cg4 = centroid of N1,C4,C5,C6,C7,C8, Cg5 = centroid of N2,C9,C10,C11,C12,C13, Cg6 = centroid of C7,C8,C9,C13,C14,C15, Cg7 = centroid of C19,C20,C21, C22, C23,C24, Cg8 = centroid of N3,C7,C8,C9,C13,C14, C15,C16,N4, Cg9 = centroid of N5,C17,C18,C19,C20,C21,C22,C23,C24, Cg10 = centroid of N1,C4,C5,C6,C7,C8,C9,C13,C14,C15, Cg11 = centroid of N2,C7,C8,C9,C10,C11,C12,C13, C14,C15, Cg12 = centroid of N1,C4,C9,C13,C14,C15,C16,N3,N4, Cg13 = centroid of N2,C7, C8,C9,C10,C11,C12,C13,C14,C15,C16,N3,N4, Cg14 = centroid of N1,N3,C4,C5, C6,C7,C8,C9,C10,C11,C12,C13,C14,C15, Cg15 = centroid of N1,N2,C4,C5,C6,C7,C8,C9, C10,C11,C12,C13,C14,C15,C16,N3,N4.

The intermolecular interactions in the crystal structure of **3a** were quantified using Hirshfeld surface analysis and the extensive π-interactions observed in this structure is confirmed and well-illustrated in the shape index (a) and curvedness plot (b) in Figure S4. The blue and red triangles in Figure S4a are characteristic of π-π-interactions and represent the convex regions (due to ring carbon atoms for the molecule inside the surface) and concave regions (due to carbon atoms of the π-stacked molecule above it) respectively. The π-π-interactions is also confirmed in the curvedness surface as the large green area in Figure S4b which is indicative of a ‘relatively flat’ region.

Figure S4. Hirshfeld surface of **3a** mapped with a) shape index and b) curvedness, illustrating the extensive π-π-interactions observed in the structure of **3a**.

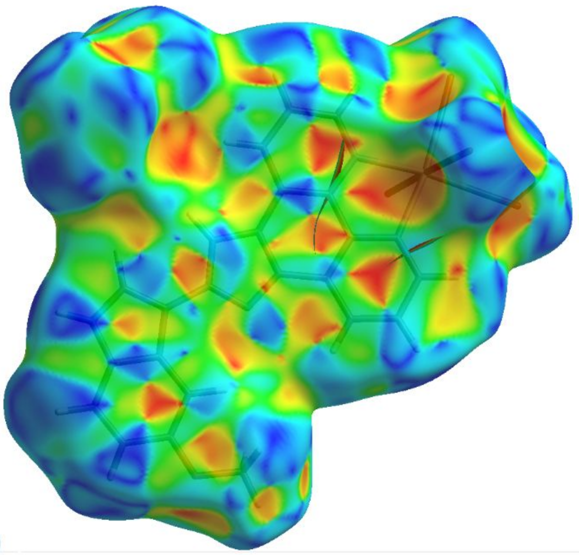

a)

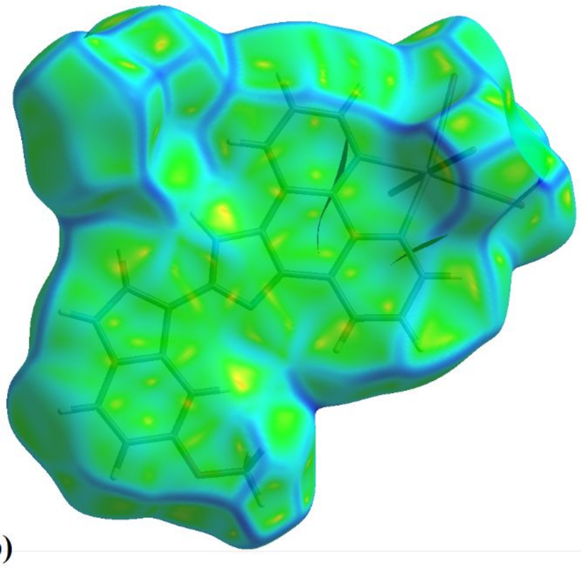

b)

The one dimensional infinite chain formed by N3-H3A···O5 and N5-H5A···Br1 is illustrated in Figure S5. In the shape index plot (Figure S5a), the red concave region around the acceptor atom O5 and the blue convex region around the donor at N5 is illustrated; this is confirmed in the d_{norm} plot (Figure S5b) with the red electronegative region around the acceptor O5 and the blue electropositive region around the donor atom N5.

Figure S5. a) Shape index plot and b) d_{norm} plot (-0.4243-1.4335) of the Hirshfeld surface analysis of **3a**.

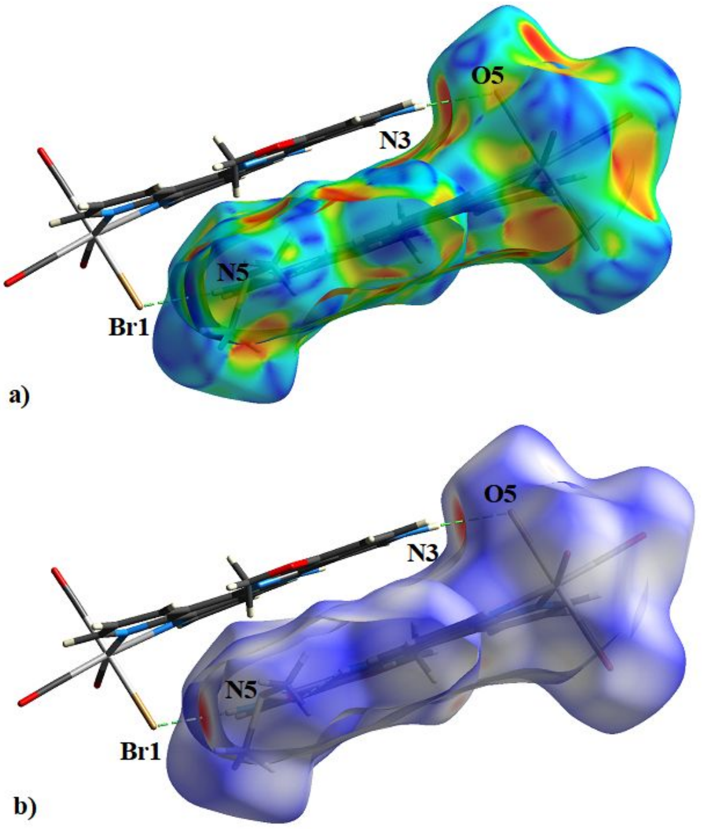

The Br…H/H…Br, C…H/H…C, H…H, and O…H/H…O interactions comprise 10.9 %, 17.3 %, 25.3 %, and 23.2 % respectively, to the total Hirshfeld surface of **3a** The fingerprint plots of **3a** are illustrated in Figure S6; it is clear that the H…H and O…H/H…O interactions contribute significantly to the packing and overall stabilization of the crystal structure.

Figure S6. Fingerprint plots of **3a** resolved into a) Br…H/H…Br contacts (10.9 %), b) C…H/H…C contacts (17.3 %), c) H…H contacts (25.3 %), and d) O…H/H…O contacts (23.2 %).

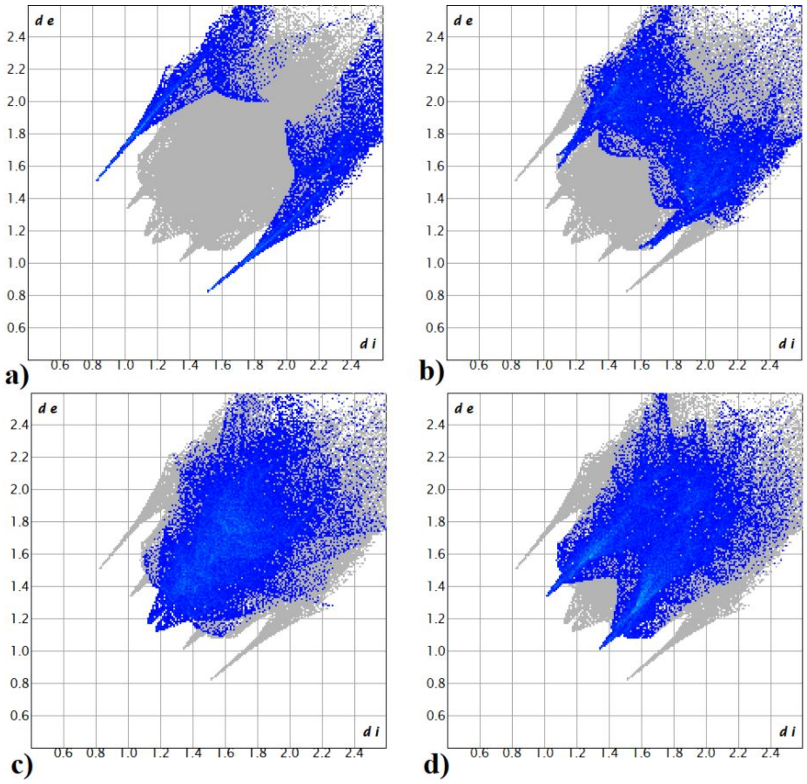

Stability study

The stability study of the complexes (**1 - 8**) was performed in DMSO, phosphate buffer pH 7.4, both in the presence and absence of NaCl (Figure S7 – S10), and in the presence of FBS (Figure S11) by UV/Vis measurements for 6 hours.

In the DMSO solution, all complexes showed stability during measurements, except complexes **6** and **8**. For complexes **6** and **8**, multiple isosbestic points were observed, suggesting DMSO coordination with the Re centre by replacing the labile water molecule.

In the buffer solution, we observed a decrease in absorbance of the complex with time without any isosbestic point or new peak. This was due to the low solubility of the complexes causing precipitation. Any degradation of solvated complexes was not observed in buffer solution at pH 7.4, both in the presence or absence of chloride. The absence of a new isosbestic point of chloride ion suggests a stable Re-OH_2 bond.

In the presence of 10% FBS, a significant increase in the solubility of the complexes was observed. Surprisingly, no new isosbestic point was observed for any cases, suggesting no covalent interaction of the complex with FBS. Despite the increase in solubility, a little amount of precipitation was observed for complexes **1**, **2**, **3** and **6**. A very little amount of precipitation was observed in our stability kinetics study, but in the cell culture medium, we did not find any visible precipitation at the highest concentration up to 24 hours.

The ESI-MS spectra of **6** and **8** are presented in Figure S12, while the ^{1}H NMR spectra of **6** and **8**, illustrating the stability of the complexes in DMSO, are presented in Figure S13 and S14.

Figure S7. The stability of complexes **1** and **2** in DMSO, 10 mM Phosphate buffer pH 7.4 and 10 mM Phosphate buffer pH 7.4 in the presence of 4 mM saline, analysed using UV/Vis absorption spectra.

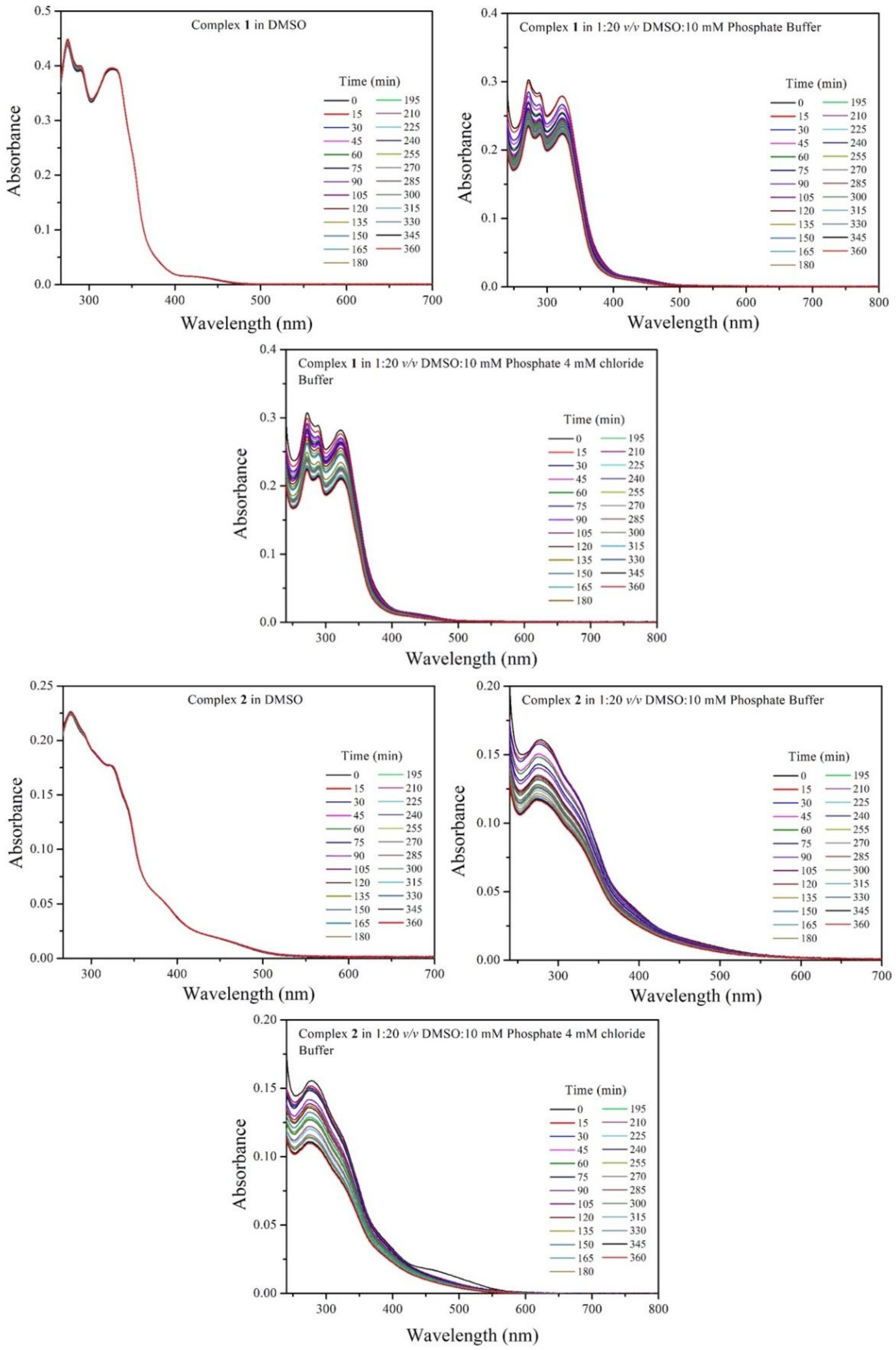

Figure S8. The stability of complexes **3** and **4** in DMSO, 10 mM Phosphate buffer pH 7.4 and 10 mM Phosphate buffer pH 7.4 in the presence of 4 mM saline, analysed using UV/Vis absorption spectra.

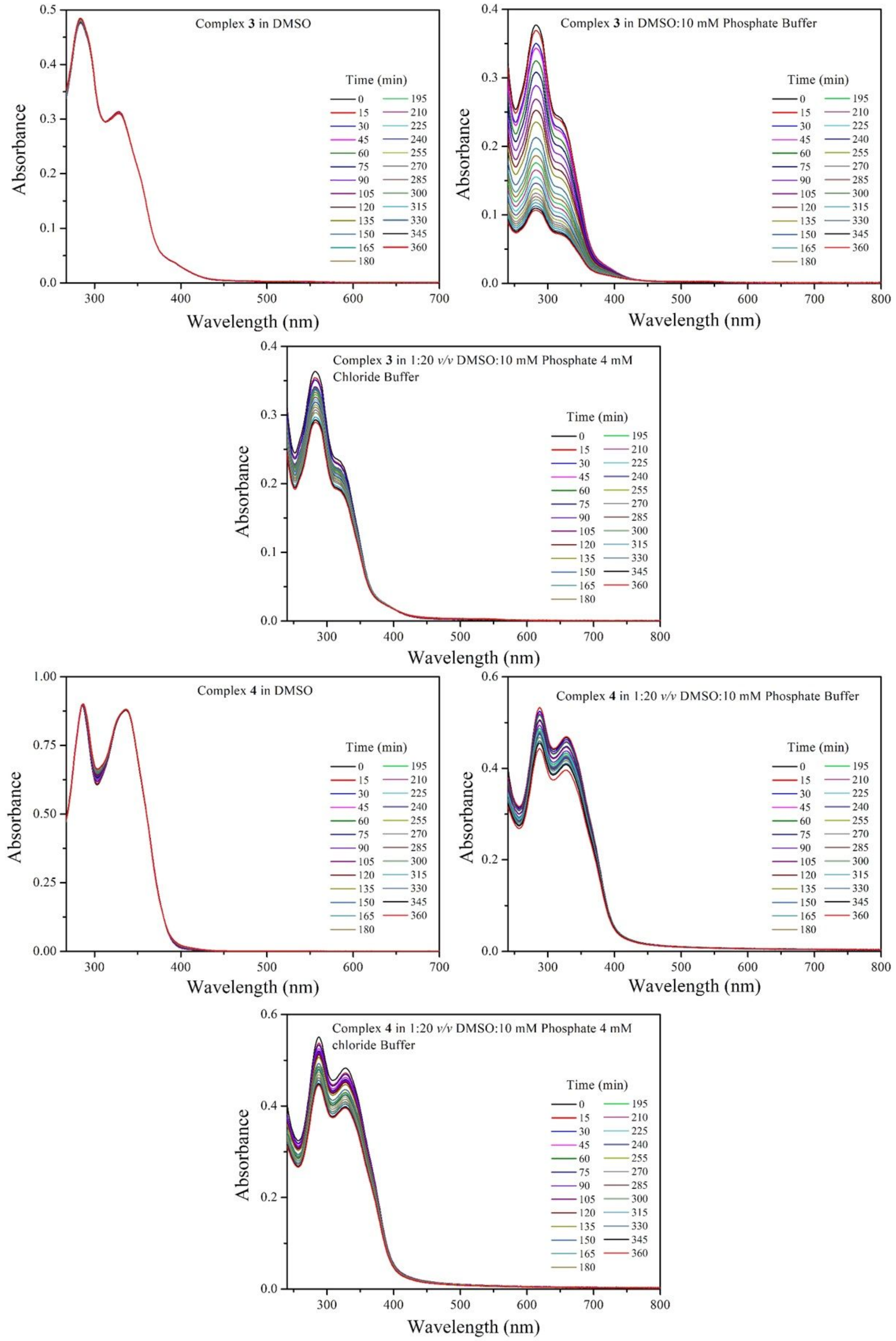

Figure S9. The stability of complexes **5** and **6** in DMSO, 10 mM Phosphate buffer pH 7.4 and 10 mM Phosphate buffer pH 7.4 in the presence of 4 mM saline, analysed using UV/Vis absorption spectra.

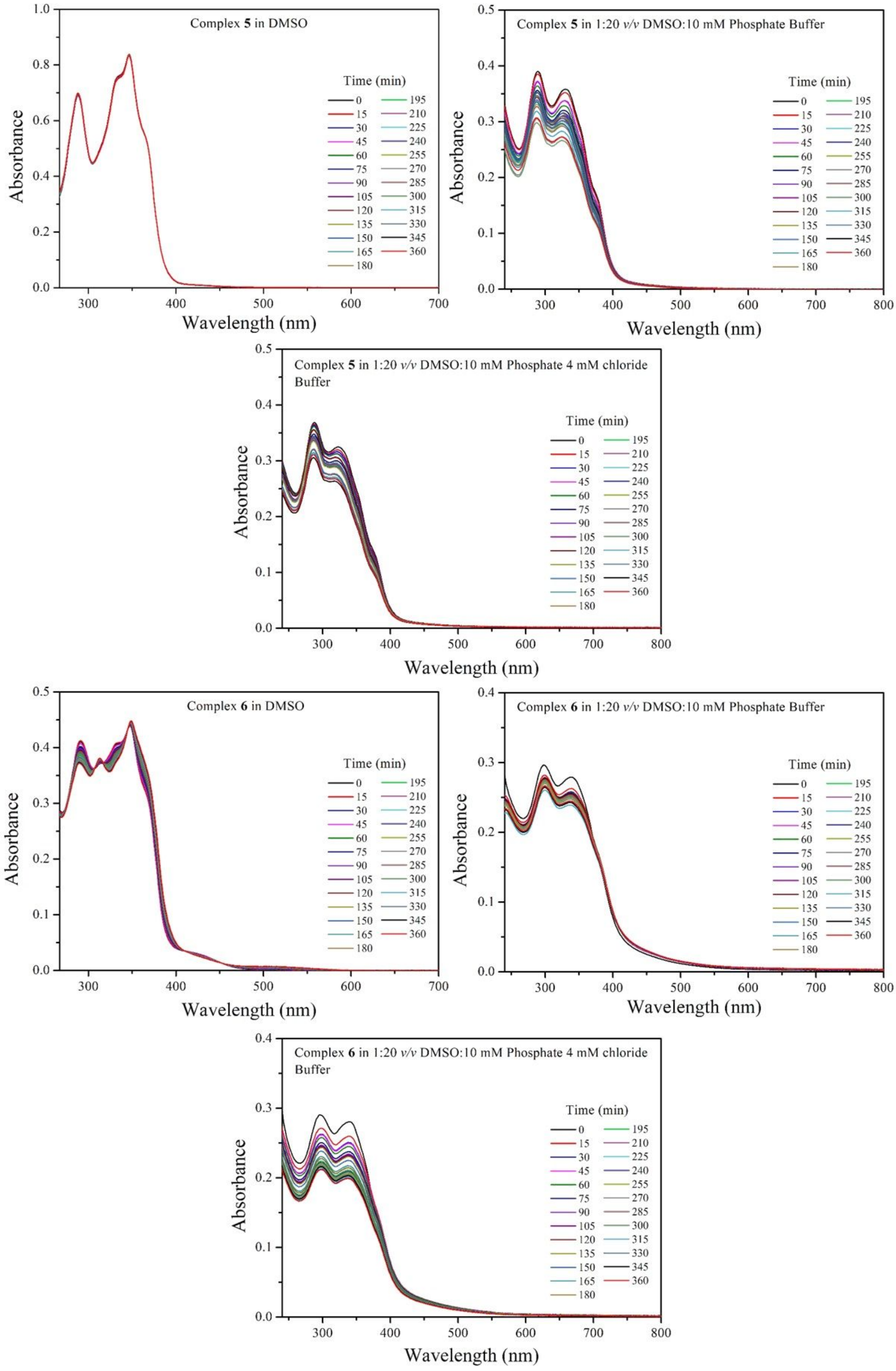

Figure S10. The stability of complexes **7** and **8** in DMSO, 10 mM Phosphate buffer pH 7.4 and 10 mM Phosphate buffer pH 7.4 in the presence of 4 mM saline, analysed using UV/Vis absorption spectra.

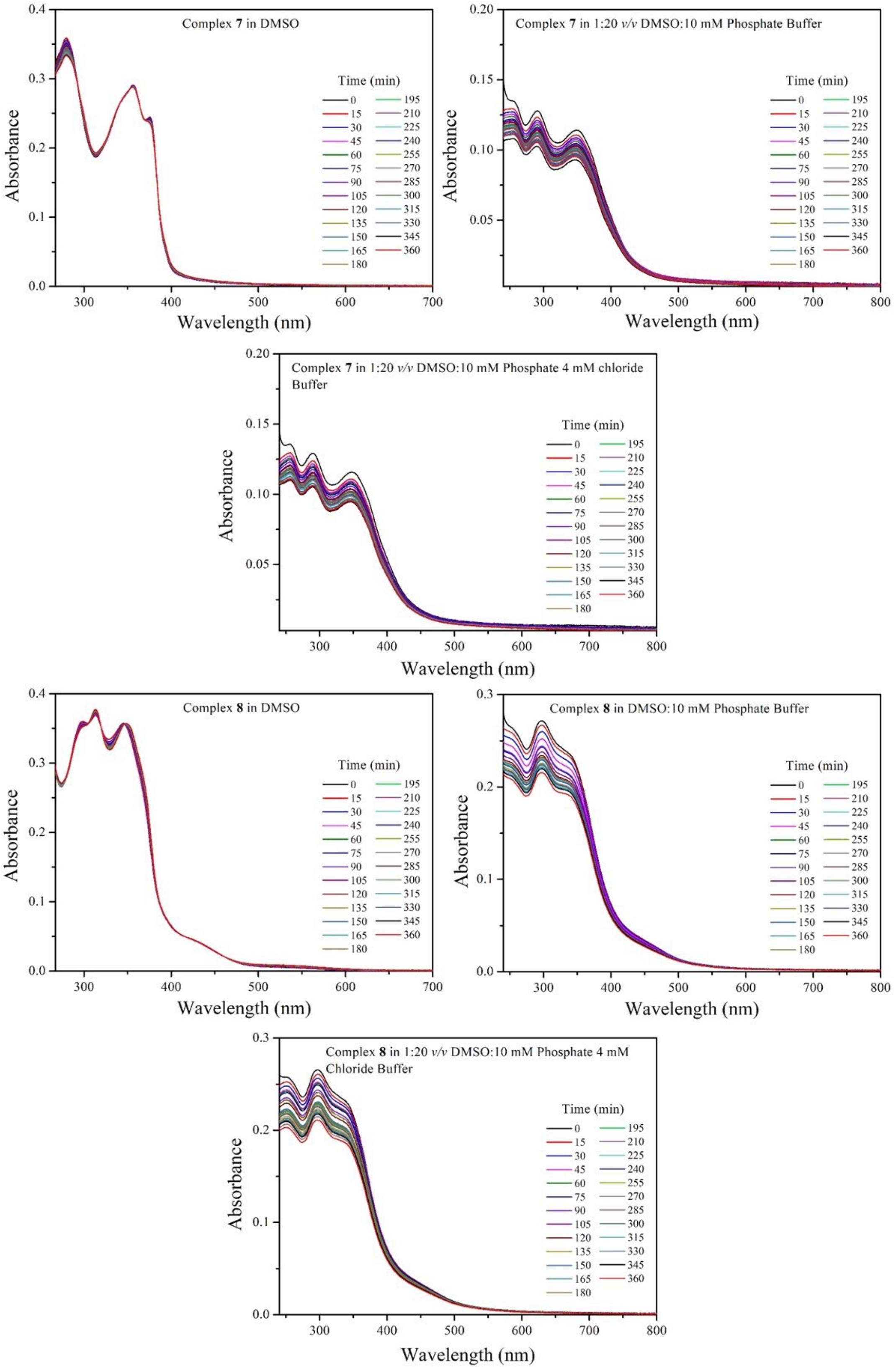

Figure S11. The stability of complexes **1 - 8** in the presence of FBS, analysed using UV/Vis absorption spectra.

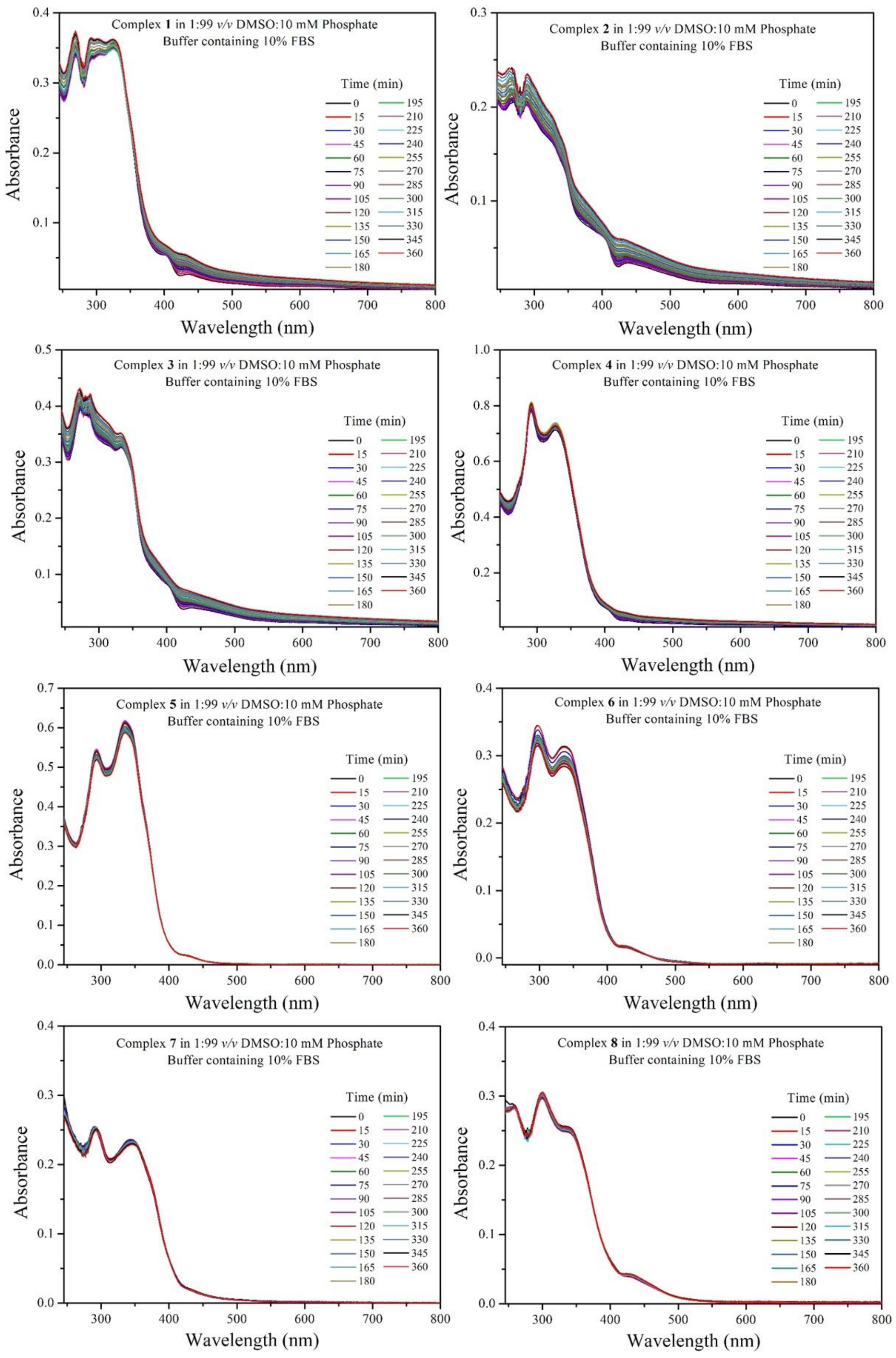

Figure S12. The ESI-MS spectra of complexes **6** and **8** in methanol after 24h incubation in DMSO.

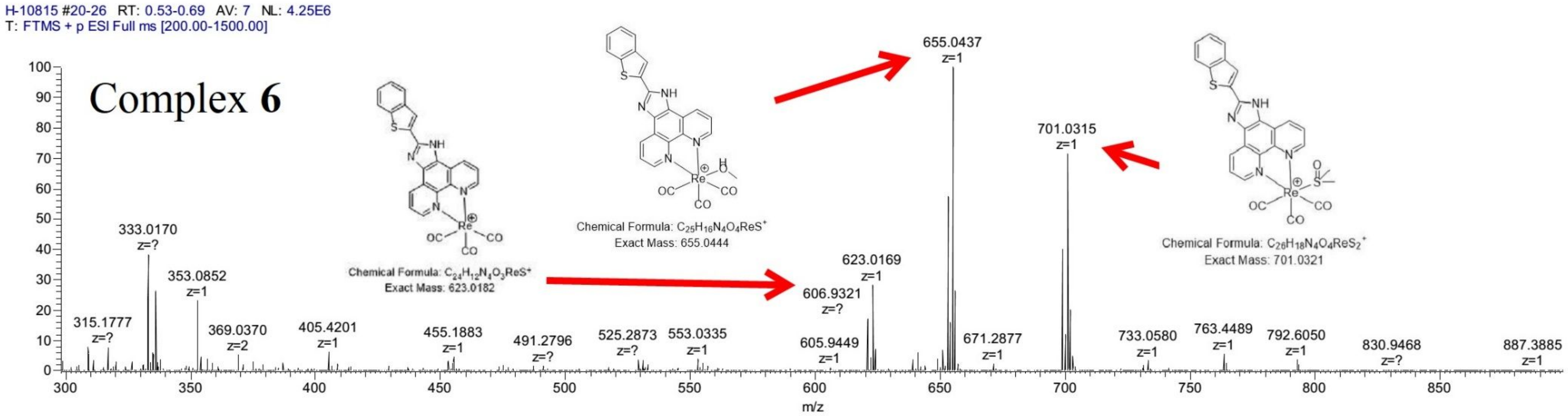

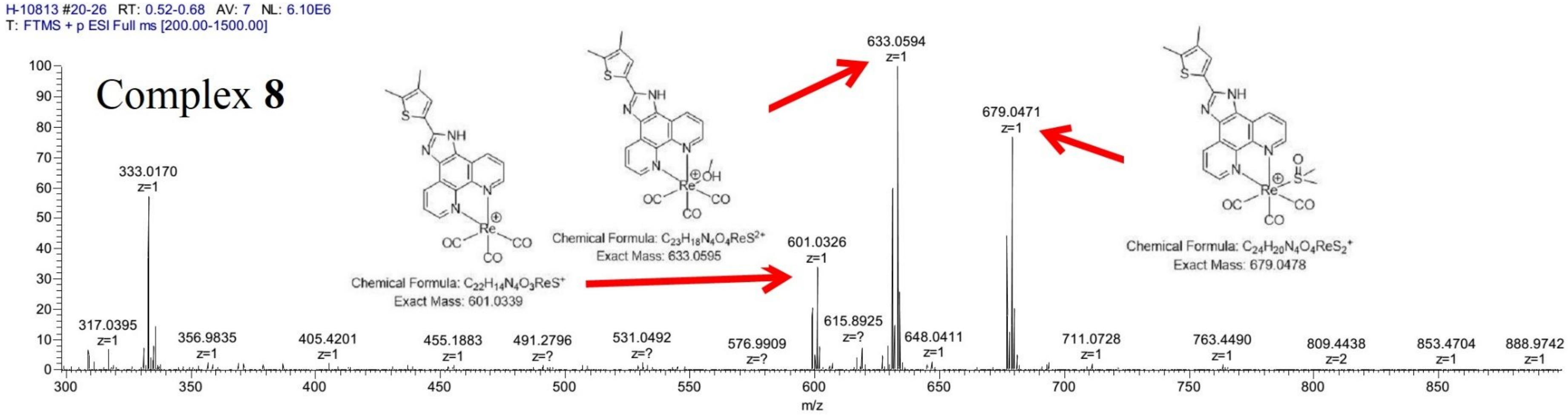

Figure S13. ^{1}H NMR spectra of *fac*-$[Re^{I}(CO)_3(L3)(H_2O)]^+$ before and after incubation in DMSO for 24 hours.

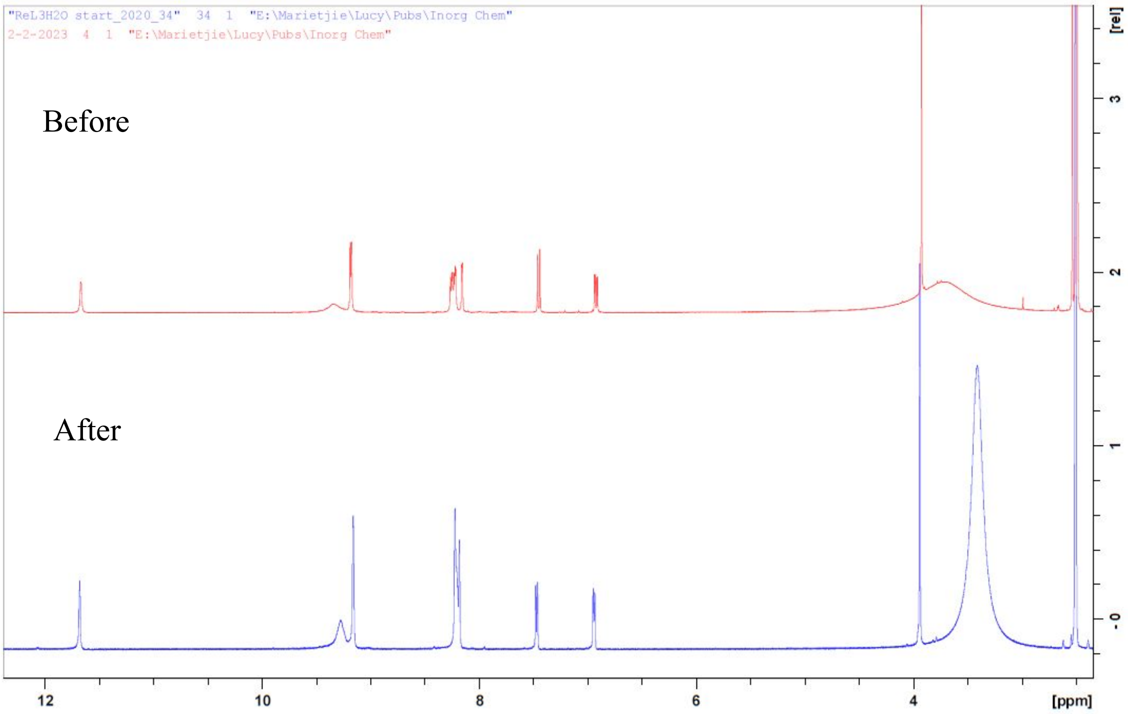

Figure S14. ^{1}H NMR spectra of (a) *fac*-$[Re(CO)_3(L6)(H_2O)]^+$ in DMSO-d6, (b) *fac*-$[Re(CO)_3(L6)(H_2O)]^+$ in DMSO-d6 after dissolution in DMSO for 6 hours, and (c) *fac*-$[Re(CO)_3(L6)(H_2O)]^+$ in DMSO-d6 after dissolution in DMSO for 24 hours. After 6 hours in DMSO, ~30% of 6 converted to the DMSO adduct, while after 24 hours, 50% DMSO adduct is visible (d). The % of adduct formation was calculated by using ^{1}H NMR integration.

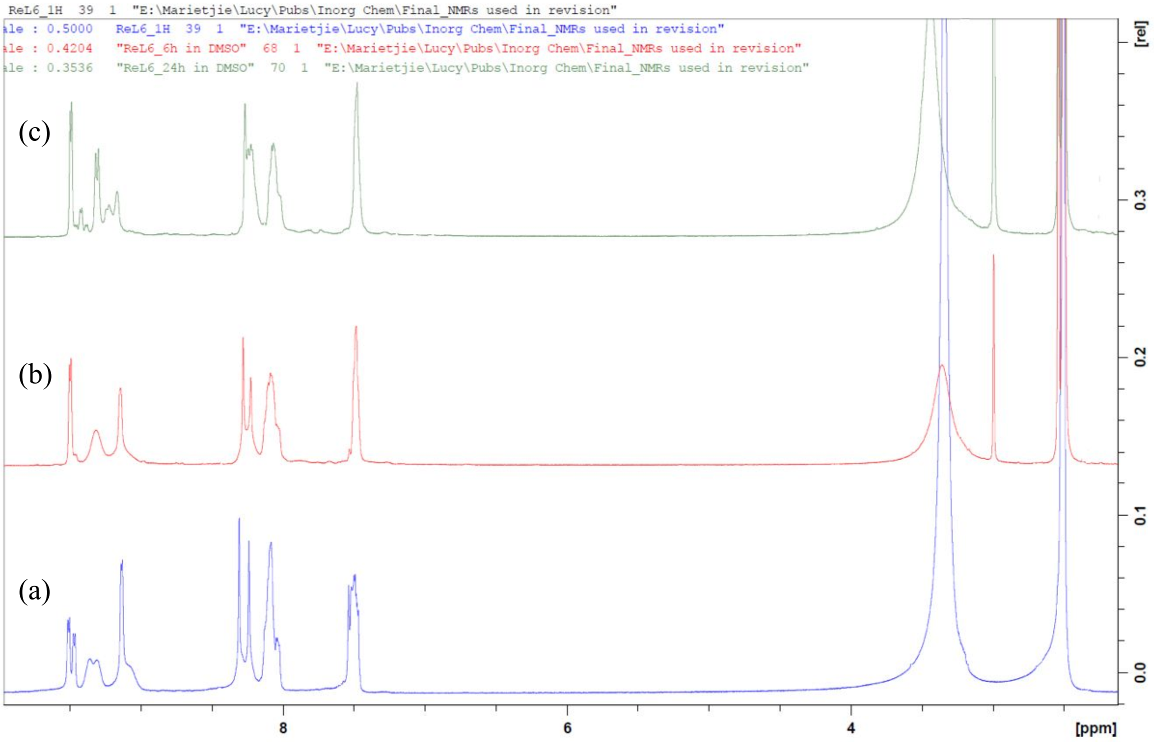

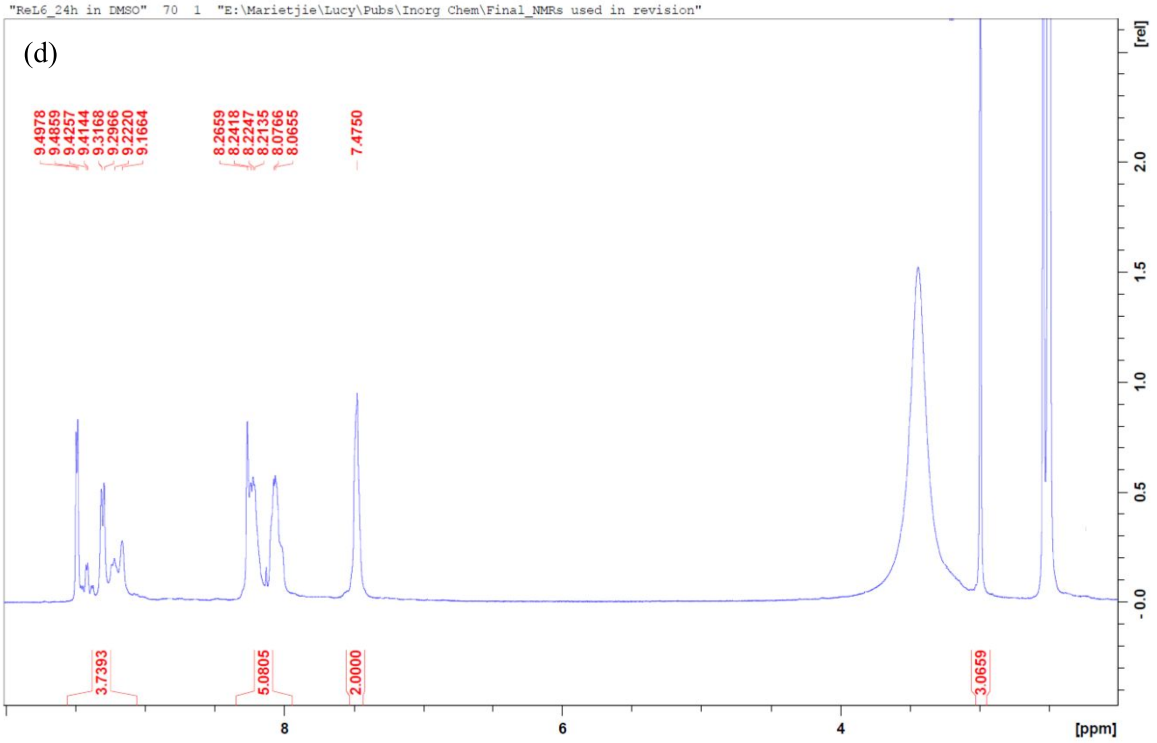

Influence of H^+ ions – Acid dissociation constant determination

The stability of *fac*-$[Re(CO)_3(L3)(H_2O)]^+$ (**3**) and *fac*-$[Re(CO)_3(L6)(H_2O)]^+$ (**6**) in DMF:H_2O (7:3, v/v %) at pH 5.5 was monitored for 24 hours (Figure S23 and S24). The UV/Vis spectra of **3** and **6** at different pH values (~pH 5.8 to ~pH 9.8) are given in Figures S25 and S26 as illustration of the change in absorbance with an increase in pH.

Figure S15. UV/Vis of *fac*-$[Re(CO)_3(L3)(H_2O)]^+$ in DMF:H_2O 7:3 (v/v %) at pH 5.5 for 24 hours.

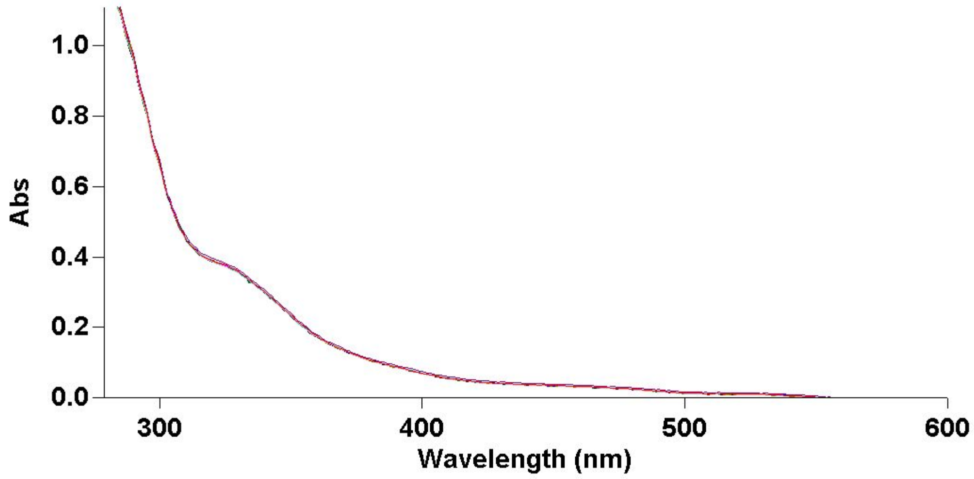

Figure S16. UV/Vis of *fac*-$[Re(CO)_3(L6)(H_2O)]^+$ in DMF:H_2O 7:3 (v/v %) at pH 5.5 for 24 hours.

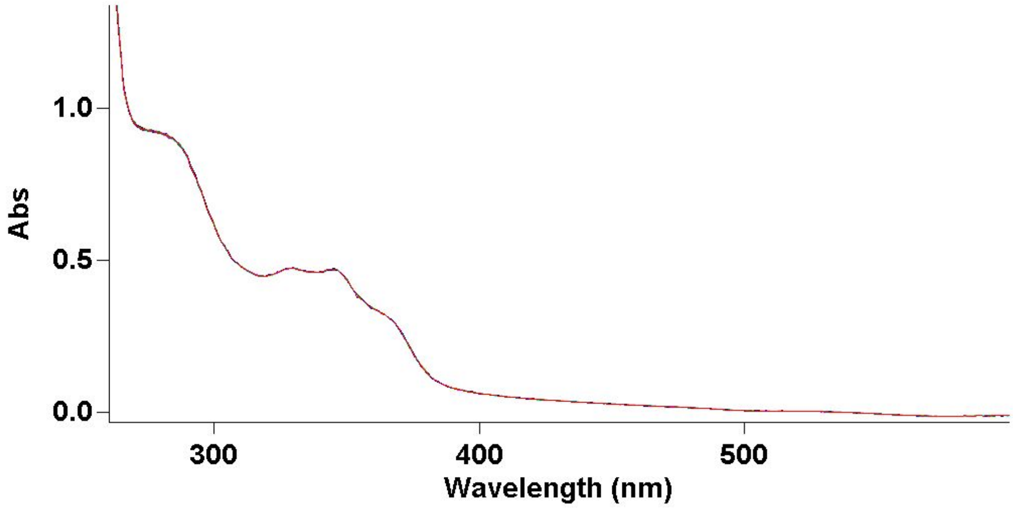

Figure S17. UV/Vis spectra of **3** at selected pH values illustrating the change in absorbance with an increase in pH.

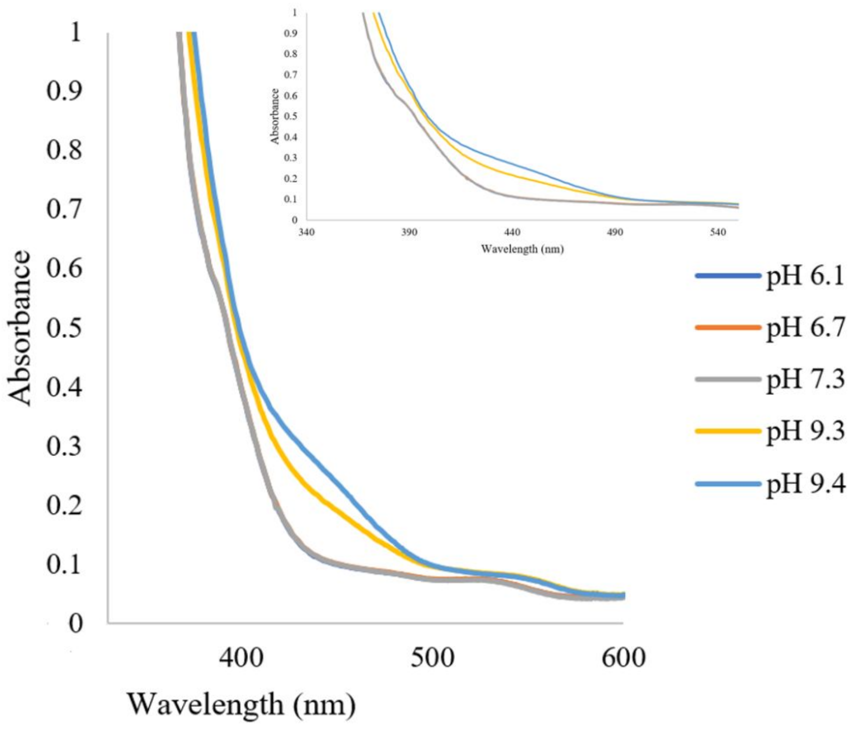

Figure S18. UV/Vis spectra of **6** at selected pH values illustrating the change in absorbance with an increase in pH.

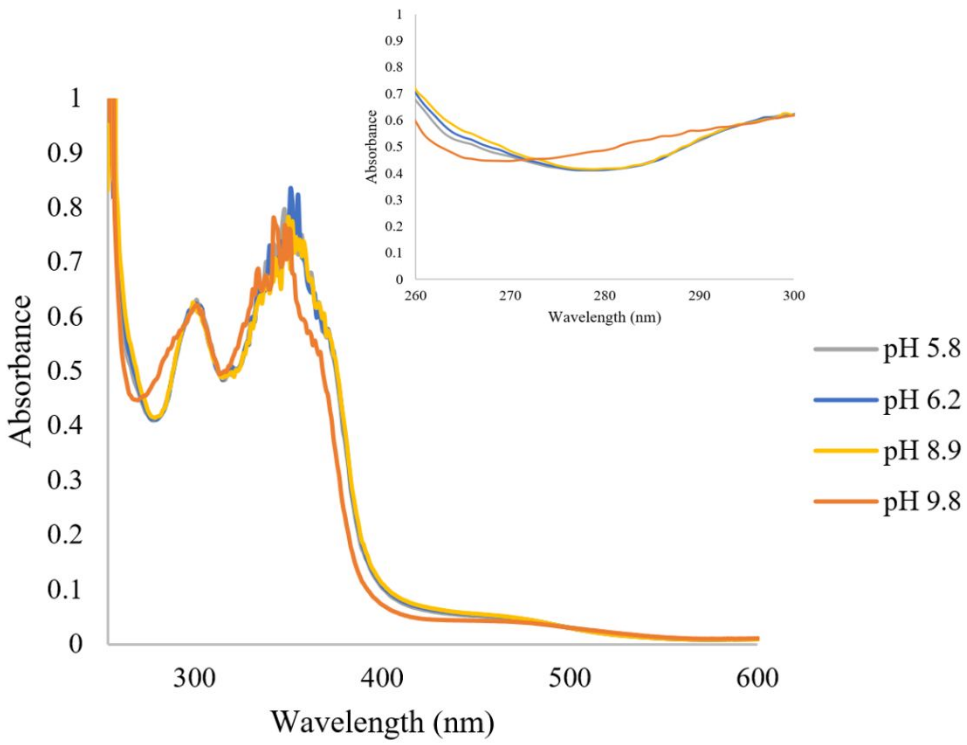

DNA binding study

The binding of **6** with guanosine was monitored by ^{1}H NMR. The different spectra of these experiments are presented in Figure S27 – Figure S30. In Figure S31, the stability of **6** in DMF is confirmed by mass spectrometry and the results of the mass spectrometry of the binding study of **6** with guanosine are provided. Figure S32 represents the binding study of **6** with ctDNA by displacing intercalated ethidium bromide (EB) to evaluate the ability of intercalation.

Figure S19. The binding study of complex **6** with Guanosine was measured by ^{1}H NMR (Aromatic region).

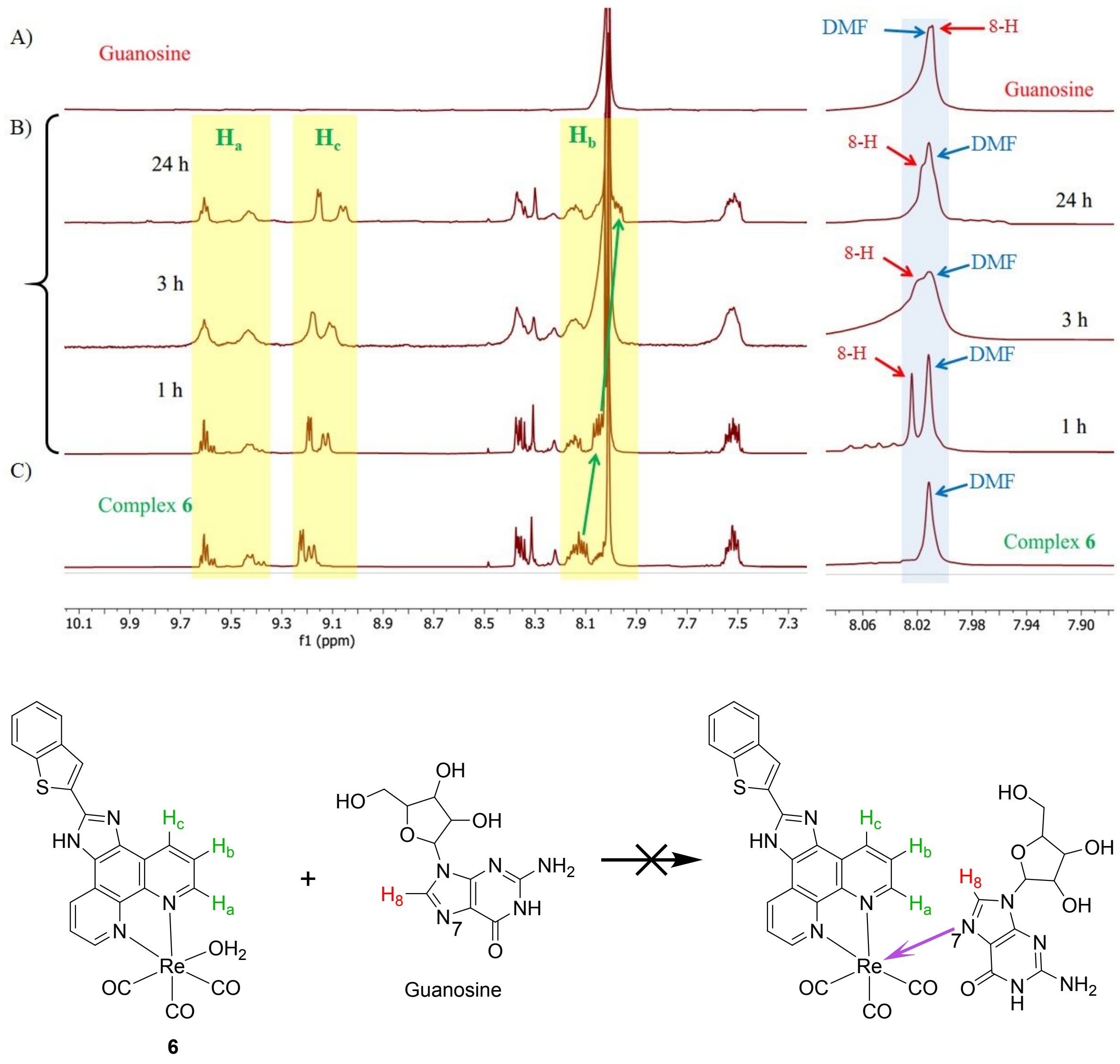

Figure S20. The binding study of complex **6** with Guanosine was measured by ^{1}HNMR.

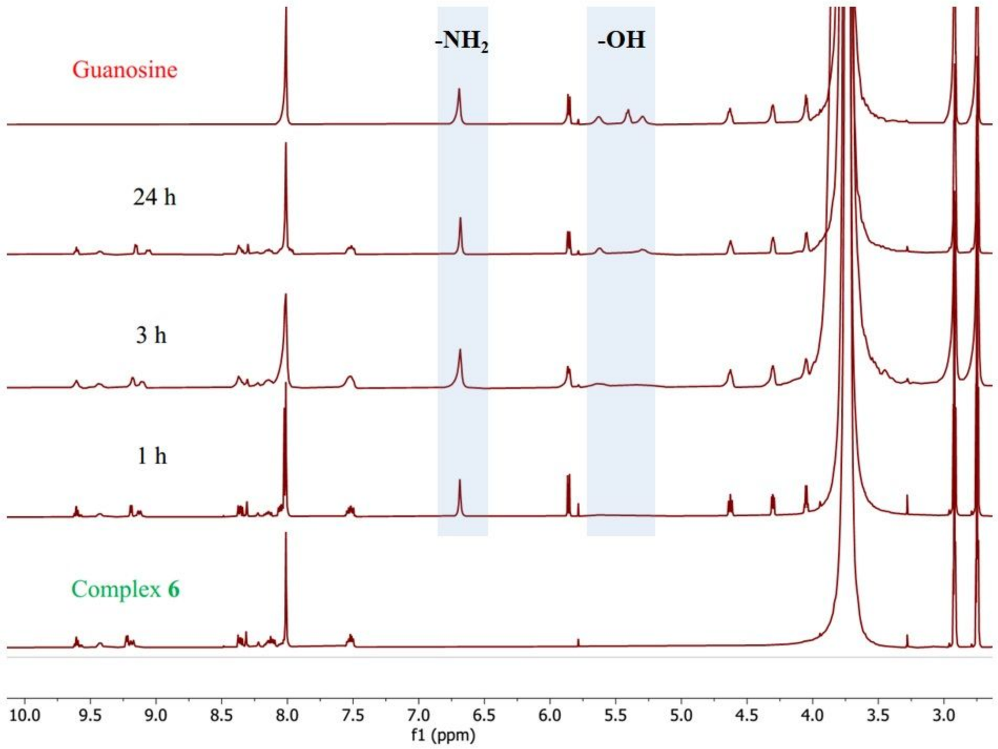

Figure S21. ^{1}H NMR spectrum of guanosine and complex **6** binding study. The study was performed in a 1:2 molar ratio of the complex: guanosine in DMF-d7 at 37°C.

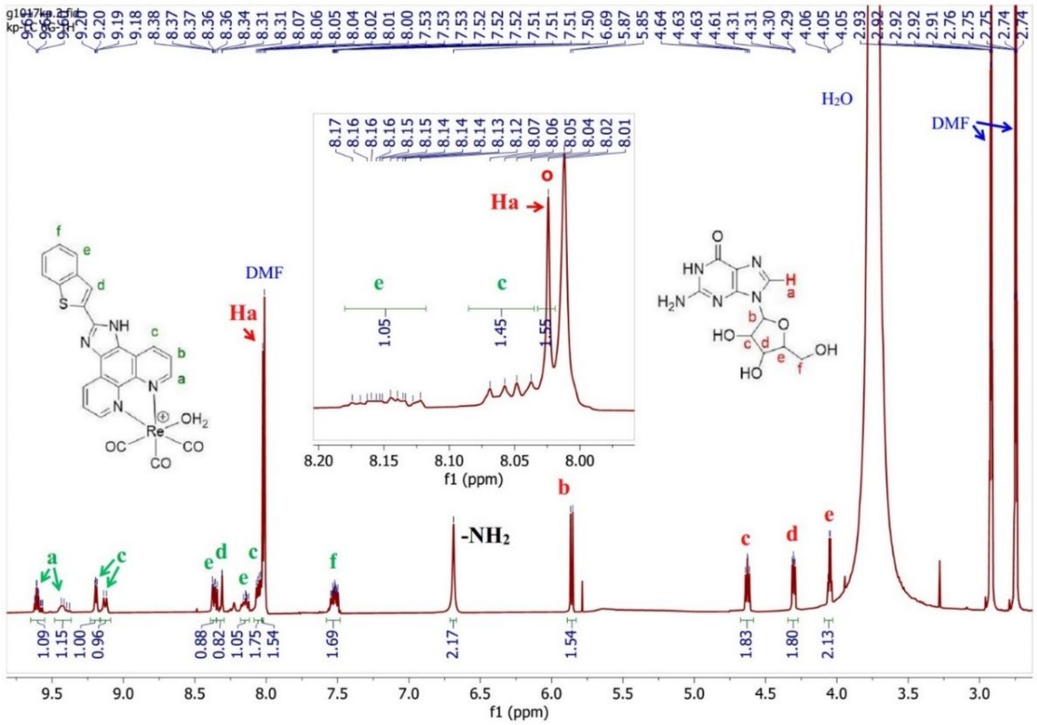

Figure S22. Representation of the stability of complex **6** (A) and model nucleobase, guanosine (B) during our experimental condition. The three peaks that appeared from 5.2 to 5.7 ppm are corresponding to three –OH protons.

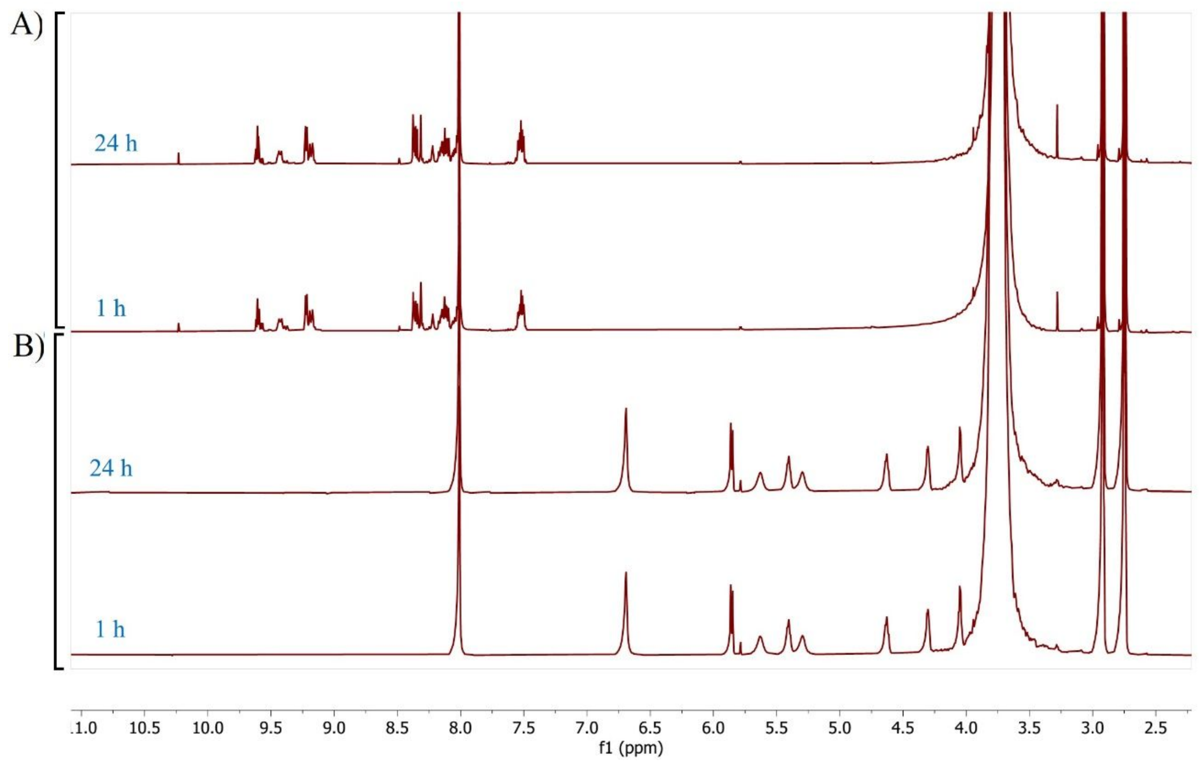

Figure S23. ESI-Mass spectra of A) complex **6** and guanosine binding study and B) stability of **6** in DMF. The solution from NMR kinetics after 24 hours was diluted in methanol and analysed by ESI-MS.

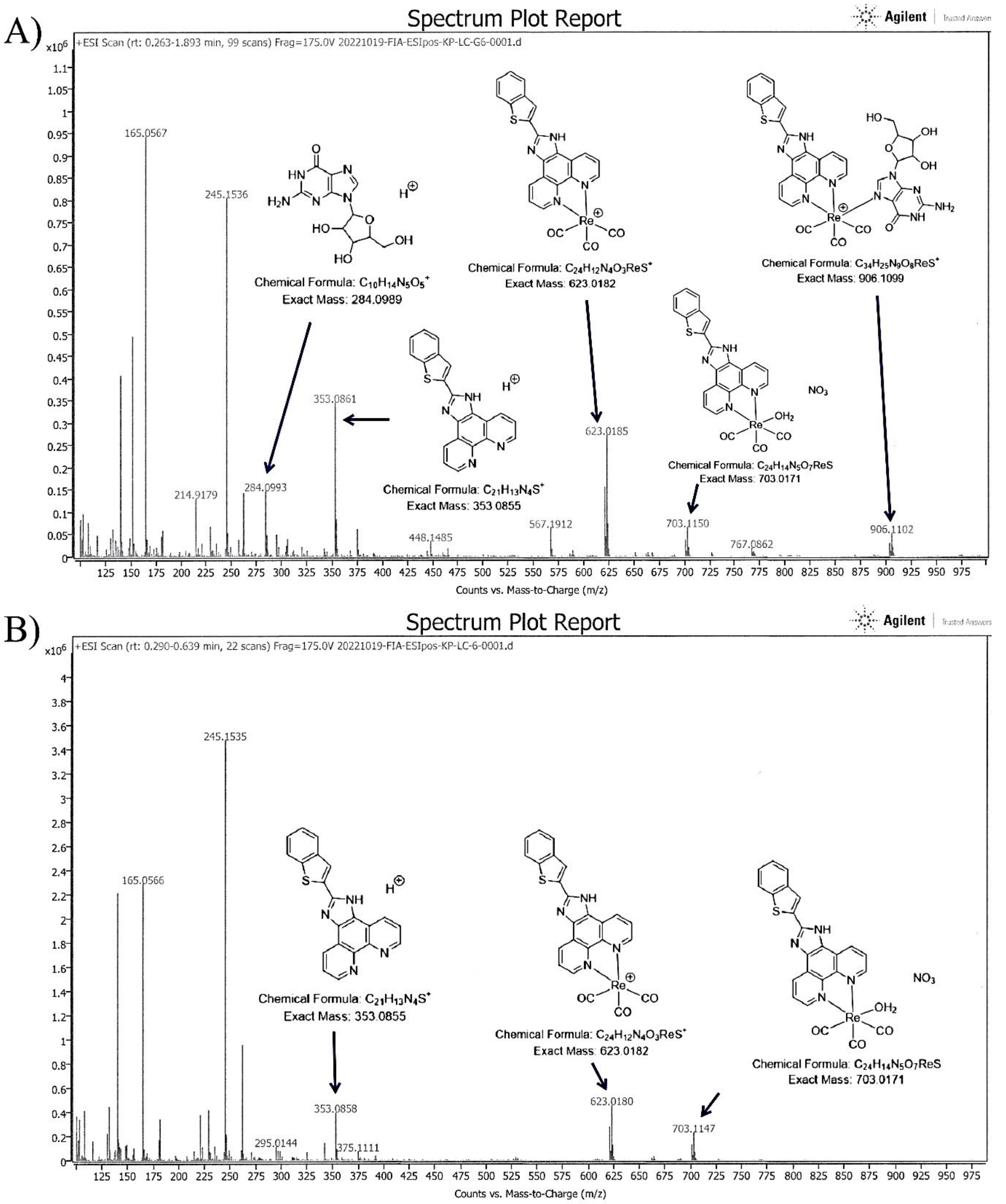

Figure S24. Complex **6** binding study with ctDNA by displacing intercalated ethidium bromide (EB) to evaluate the ability of intercalation. A) The quenching of fluorescence (Ex: 540 nm and Em: 550-800 nm) originated from EB intercalated ctDNA indicates the replacement of EB with the complex. B) The plot of normalized fluorescence emission maxima against complex

concentration (µM) is used to determine the C50. Where the C_{50} represents the 50% fluorescence quenching. The representative plots are of one experiment out of three.

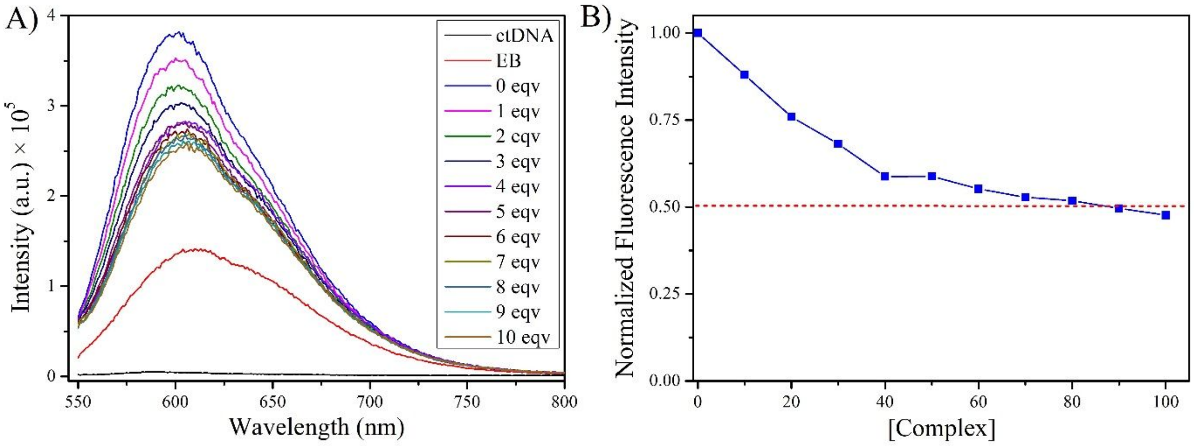

BSA binding

The Stern-Volmer and Scatchard plots for the BSA fluorescence quenching of **1** – **8** are presented in Figure S33 – Figure S36 below.

Figure S25. The Stern–Volmer and Scatchard plots for the BSA fluorescence quenching upon the addition of complexes **1** and **2**.

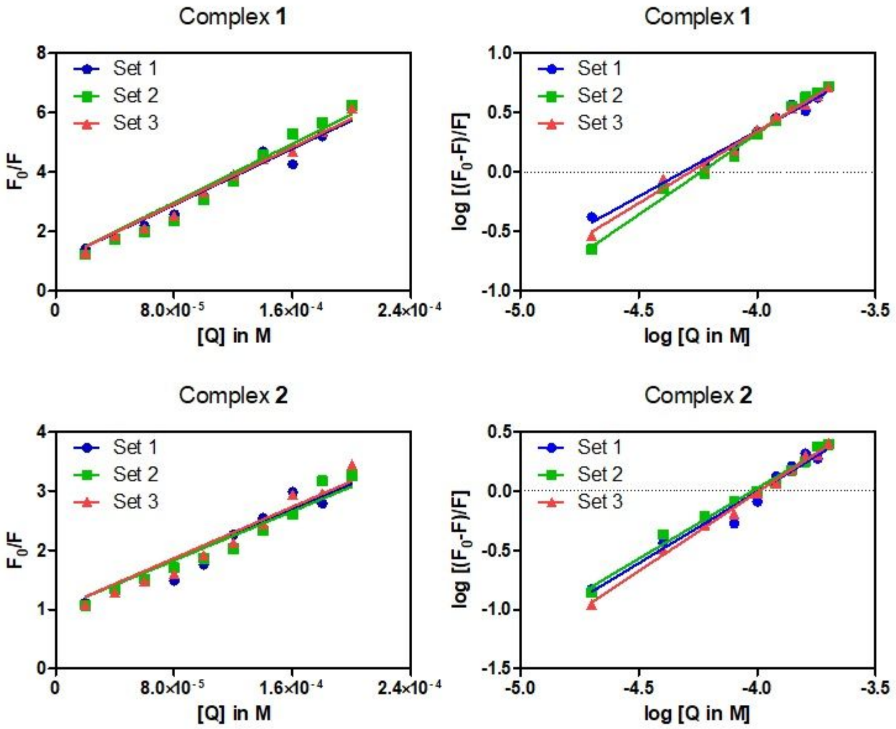

Figure S26. The Stern–Volmer and Scatchard plots for the BSA fluorescence quenching upon the addition of complexes **3** and **4**.

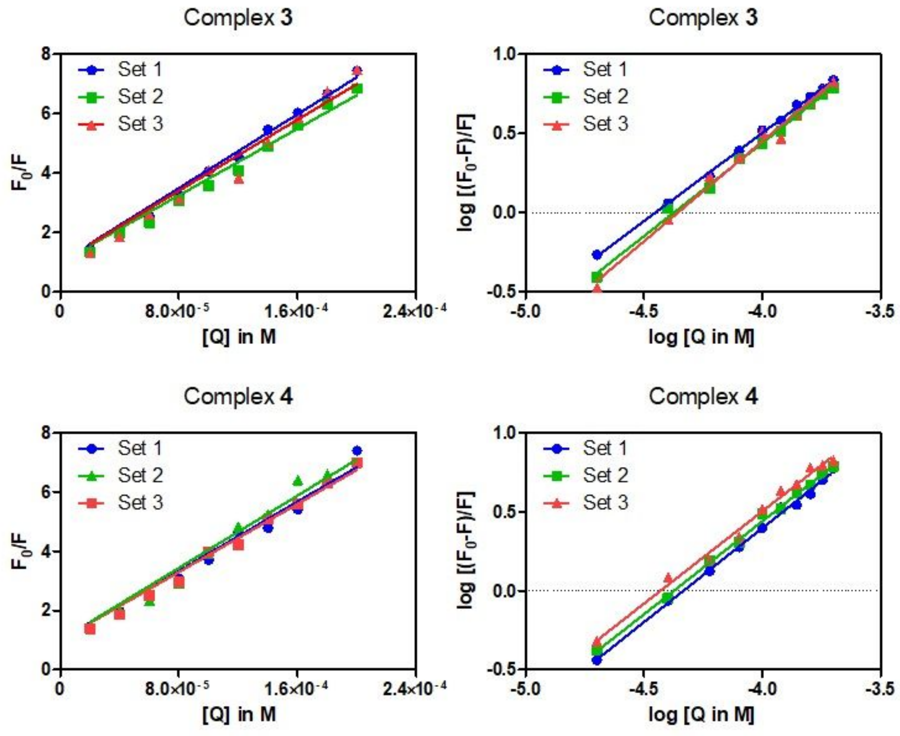

Figure S27. The Stern–Volmer and Scatchard plots for the BSA fluorescence quenching upon the addition of complexes **5** and **6**.

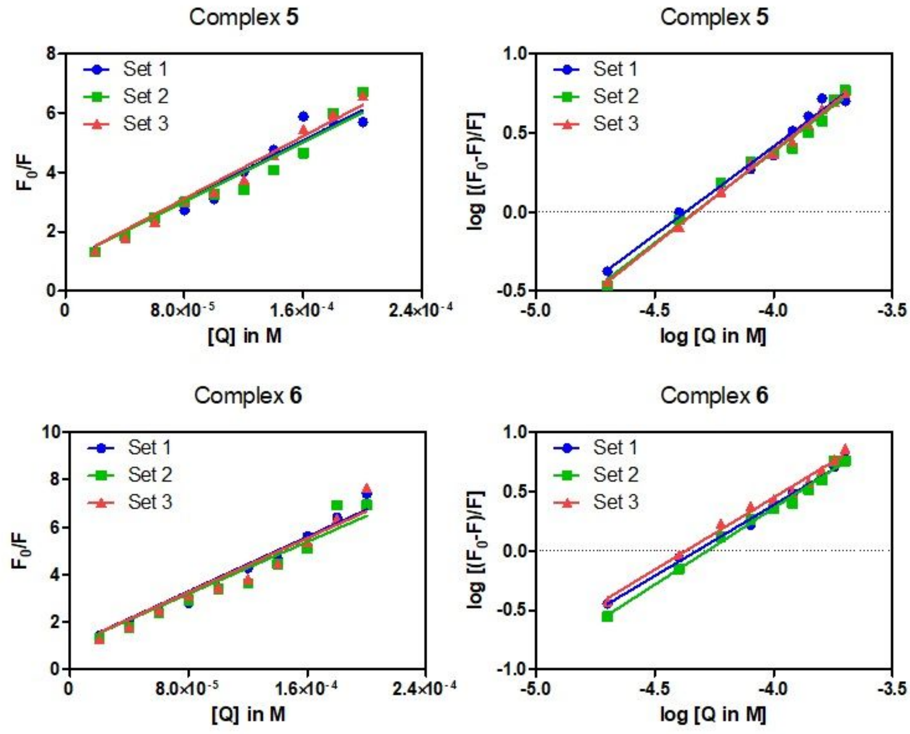

Figure S28. The Stern–Volmer and Scatchard plots for the BSA fluorescence quenching upon the addition of complexes **7** and **8**.

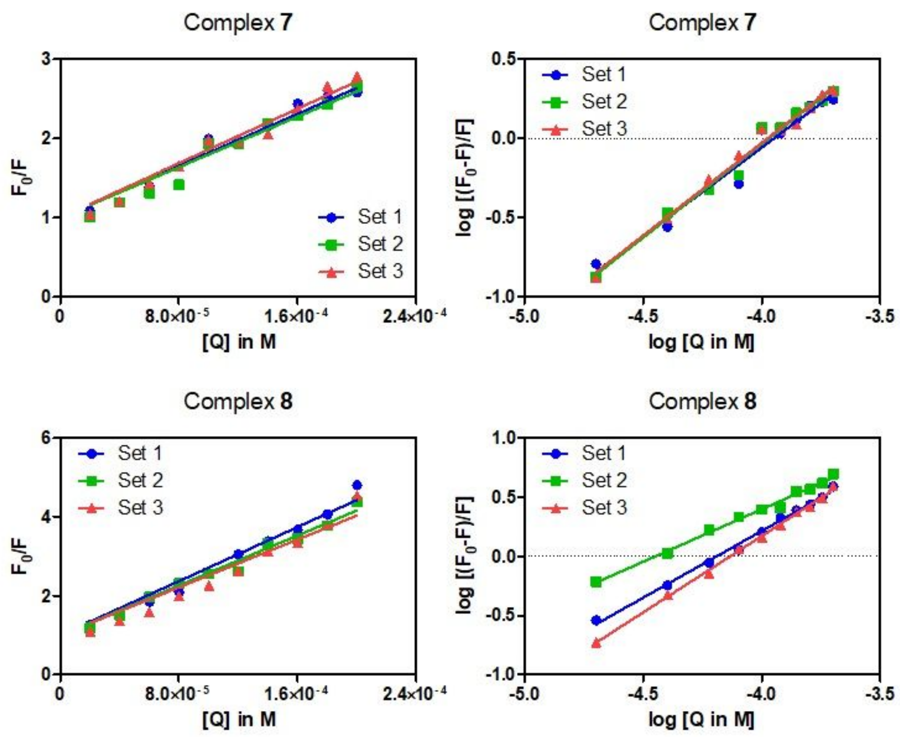

Cytotoxicity

The percentage cell viability vs. log of concentration (nM) graphs for **3**, **6**, and cisplatin against human prostate adenocarcinoma (PC-3) cells are presented in Figures S37, S39, and S40. The plots of tirapazamine against PC-3 cells in normoxic and hypoxic conditions are given in Figure S38 and the representative plots of cisplatin against PC-3 cells are provided in Figure S41.

Table S4. Cytotoxicity of complexes **3** and **6** against PC3 cells in hypoxic condition (2% O_2) in comparison to cisplatin.

Complex	IC_{50} (μM) ± S.D.[a]		Hypoxia resistance factor[b]
	Normoxia	Hypoxia	
**3**	0.32 ± 0.03	5.5 ± 1.3	17.2
**6**	0.05 ± 0.01	7.0 ± 2.2	140
Cisplatin	6.90 ± 0.50	>50	>7
Tirapazamine[d]	~100[c]	20.40 ± 3.00	0.2

[a]S.D. is the standard deviation. [b]Hypoxia resistance factors stand for the ratio of IC_{50} in hypoxia and normoxia. [c]At maximum tested concentration (100 μM) we observed around 50% of cell killing in comparison to the control. [d]Tirazapamine was used as a positive control.[1,2] The IC_{50} value was determined using the standard Resazurin assay after 48 h of treatment under hypoxic conditions (~ 2 % O_2 level).

Figure S29. Representative plots of % of cell viability vs. log of concentration (nM) against human prostate adenocarcinoma (PC-3) cells for **3**, **6** and cisplatin respectively. The cell viability was determined using a Resazurin assay after 48 h of treatment with respective complex in hypoxic condition (2% O_2). Each representative data point is the average of six data points with standard deviations.

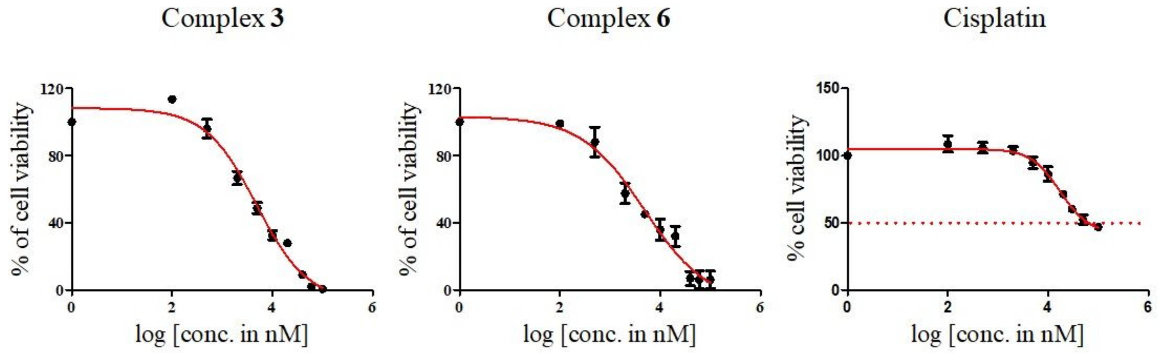

Figure S30. Representative plots of % of cell viability vs. log of concentration (nM) of tirapazamine against human prostate adenocarcinoma (PC-3) cells in normoxic and hypoxic conditions. The cell viability was determined using a Resazurin assay after 48 h of treatment with respective complexes in normoxic (21% O_2) and hypoxic conditions (2% O_2). Each representative data point is the average of six data points with standard deviations.

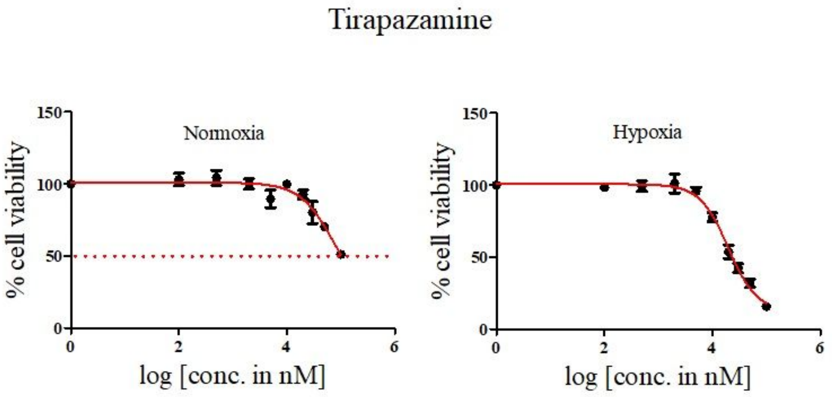

Figure S31. Representative plots of % of cell viability vs. log of concentration (nM) against human prostate adenocarcinoma (PC-3) cells for **3**. The cytotoxicity was determined after 36 h of complex incubation in normoxic conditions. The last four plots are corresponding to 1 hour of inhibitor pre-treated PC3 cells before complex treatment (details in the experimental section).

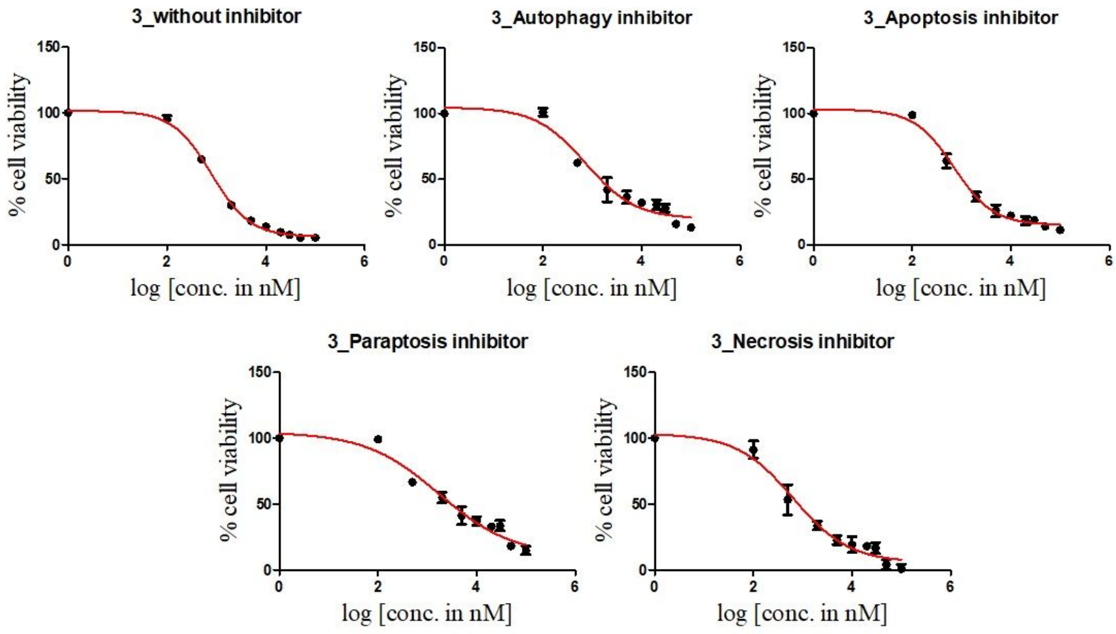

Figure S32. Representative plots of % of cell viability vs. log of concentration (nM) against human prostate adenocarcinoma (PC-3) cells for **6**. The cytotoxicity was determined after 36 h of complex incubation in normoxic conditions. The last four plots are corresponding to 1 hour of inhibitor pre-treated PC3 cells before complex treatment (details in the experimental section).

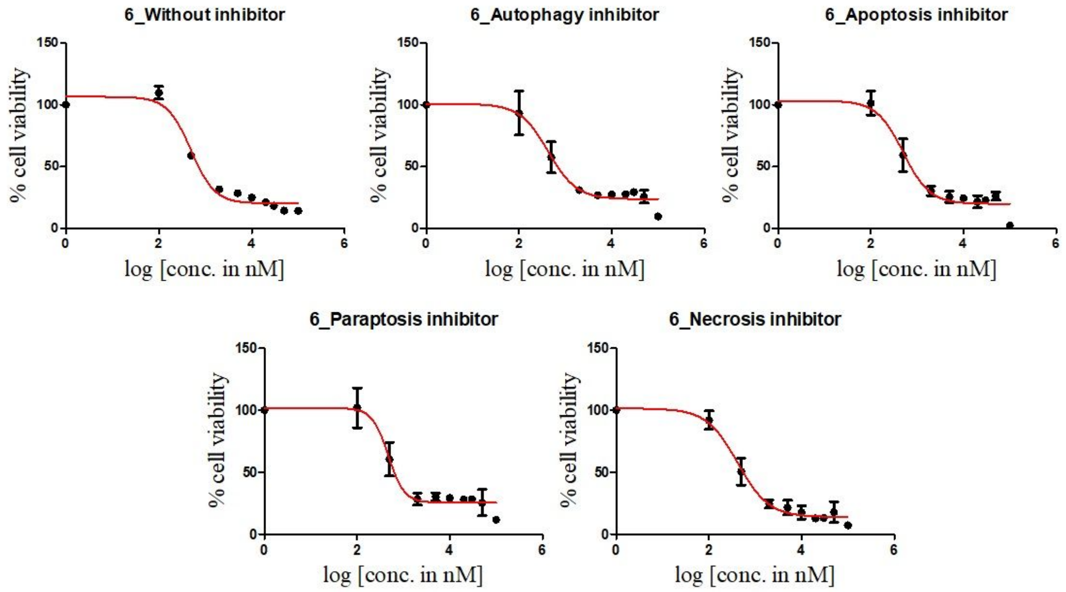

Figure S33. Representative plots of % of cell viability vs. log of concentration (nM) against human prostate adenocarcinoma (PC-3) cells for cisplatin. The cytotoxicity was determined after 36 h of complex incubation in normoxic conditions. The last four plots are corresponding to 1 hour of inhibitor pre-treated PC3 cells before complex treatment (details in the experimental section).

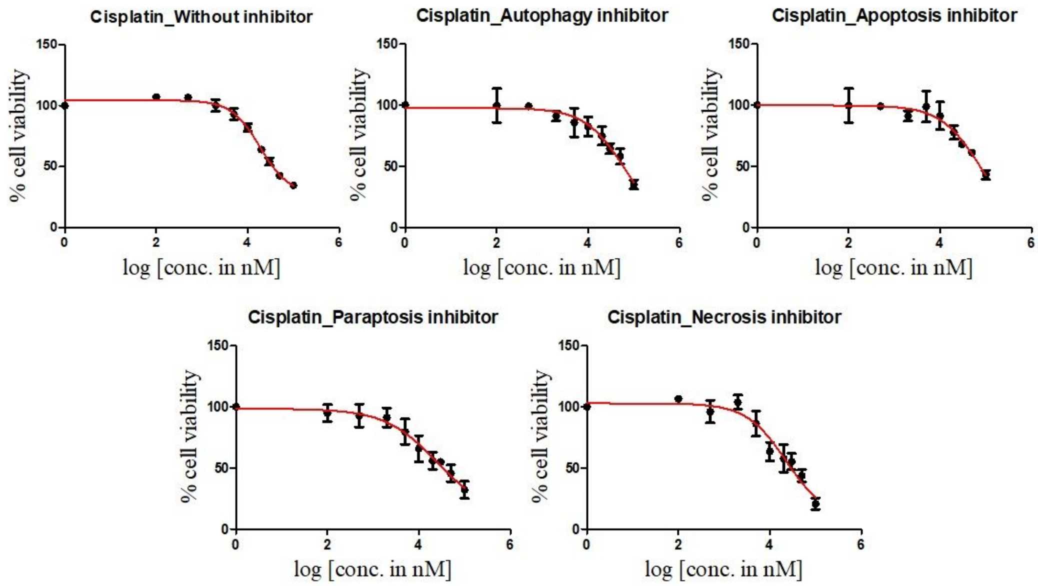

Mitochondrial respiration test

The formation of vacuoles inside the PC3 cells that are treated with **3** indicates paraptosis and are illustrated in Figure S42. A representative image of DAPI stained cells are provided in Figure S43, normalized oxygen consumption rates and different respiration parameters of PC3 cell treated with **3** and **6** are provided in Figure S44, and a representation of the DCF fluorescence intensity is given in Figure S45.

Figure S34. The formation of vacuoles inside the complex **3** treated PC3 cells indicates paraptosis. The cells were treated with IC_{50} concentration of **3** for 24h in normoxic condition before imaging (left; 10X objective bright field images, and right; 40X objective bright field images).

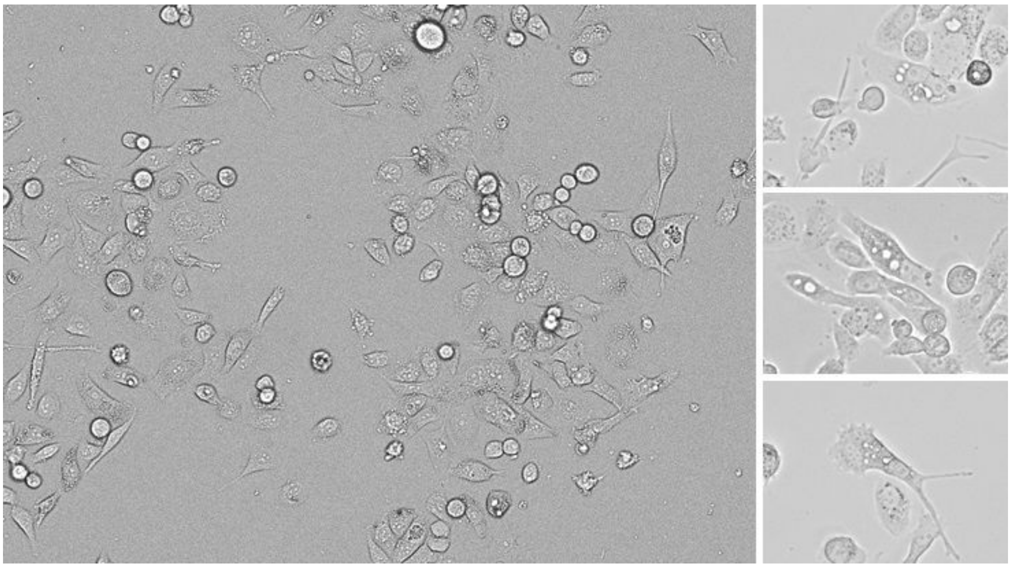

Figure S35. One representative image of DAPI stained cells, which were imaged (20X) and counted in Gen 5 software programme using Cytation 5, imagining facility, BioTek. The number of cells was used to normalise the obtained data as per experimental requirements.

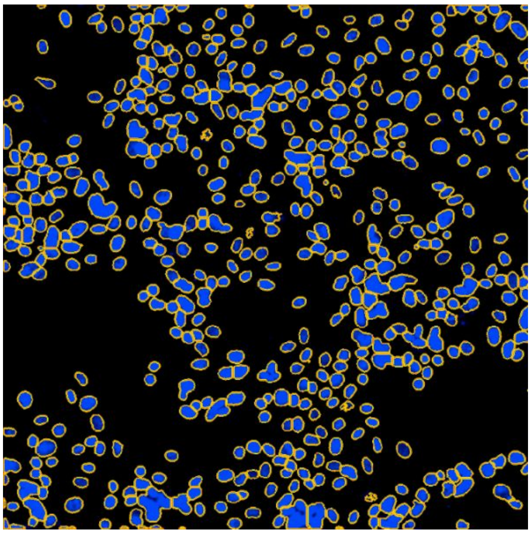

Figure S36. Representation of normalized oxygen consumption rates (OCR, pmol/min) and different respiration parameters of complex treated PC3 cells. The cells were treated with two different concentrations of complexes **3**, **6** and cisplatin in normoxic conditions for 24 hours at 37 °C.

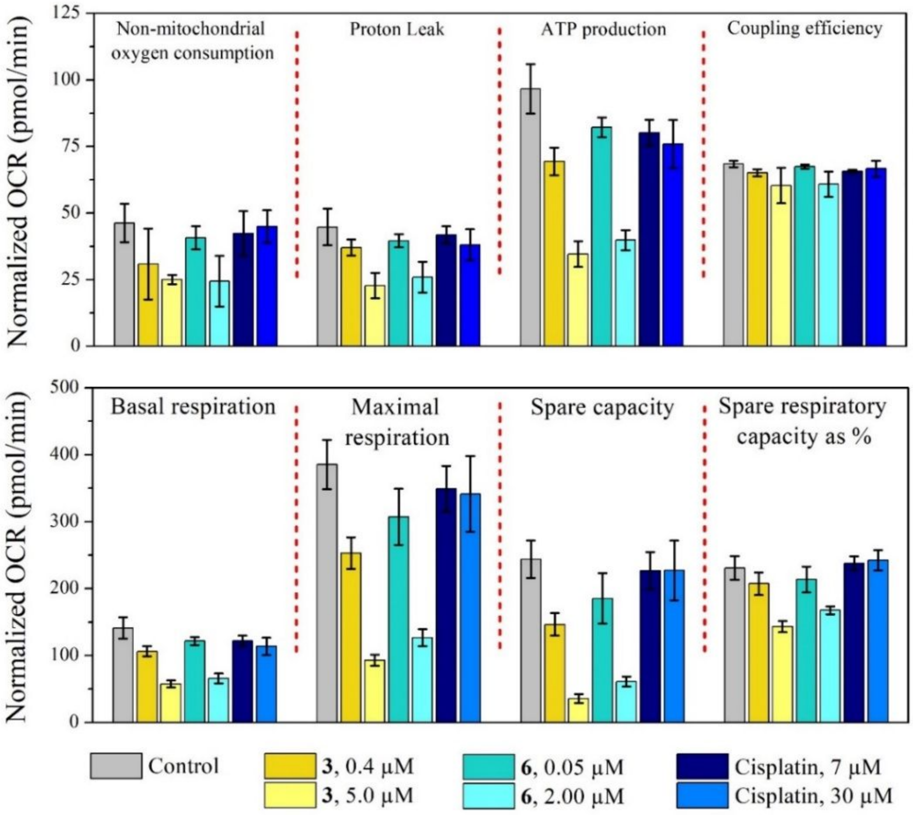

Figure S37. The figure represents DCF fluorescence intensity. The complex or H_2O_2 (positive control) treated cells were incubated with 40 μM DCFH-DA to react with produced ROS inside the cell.

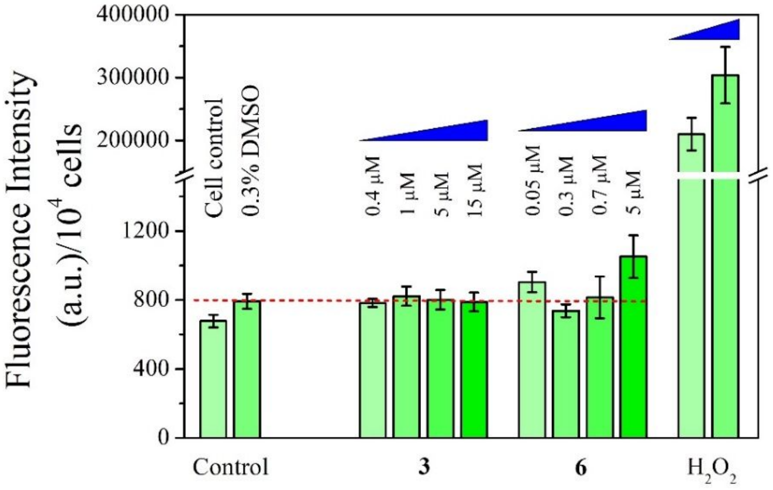

^{1}H NMR spectra of complexes

Figure S38. ^{1}H NMR spectra of **1**.

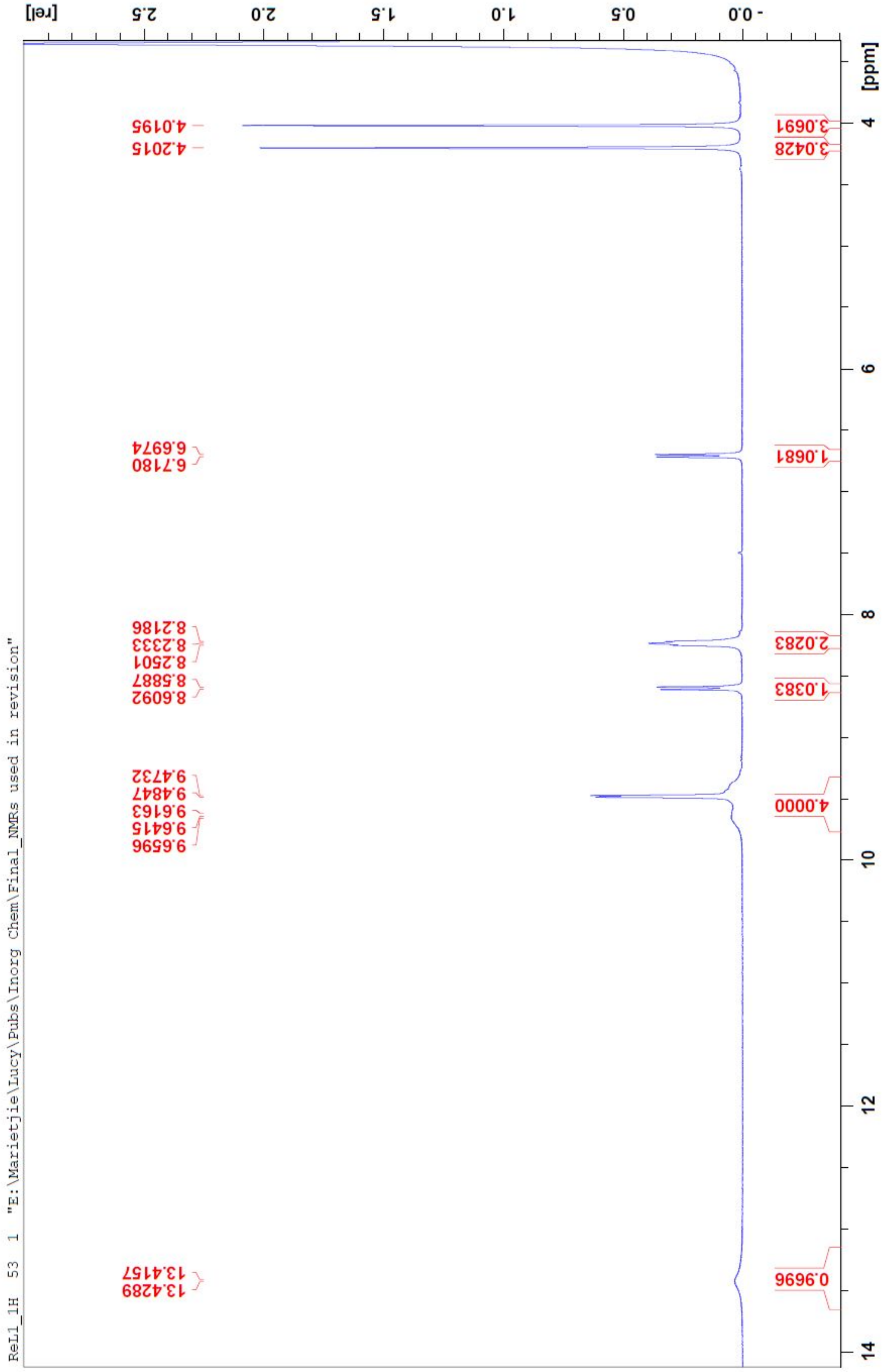

Figure S39. ^{1}H NMR spectra of **2**.

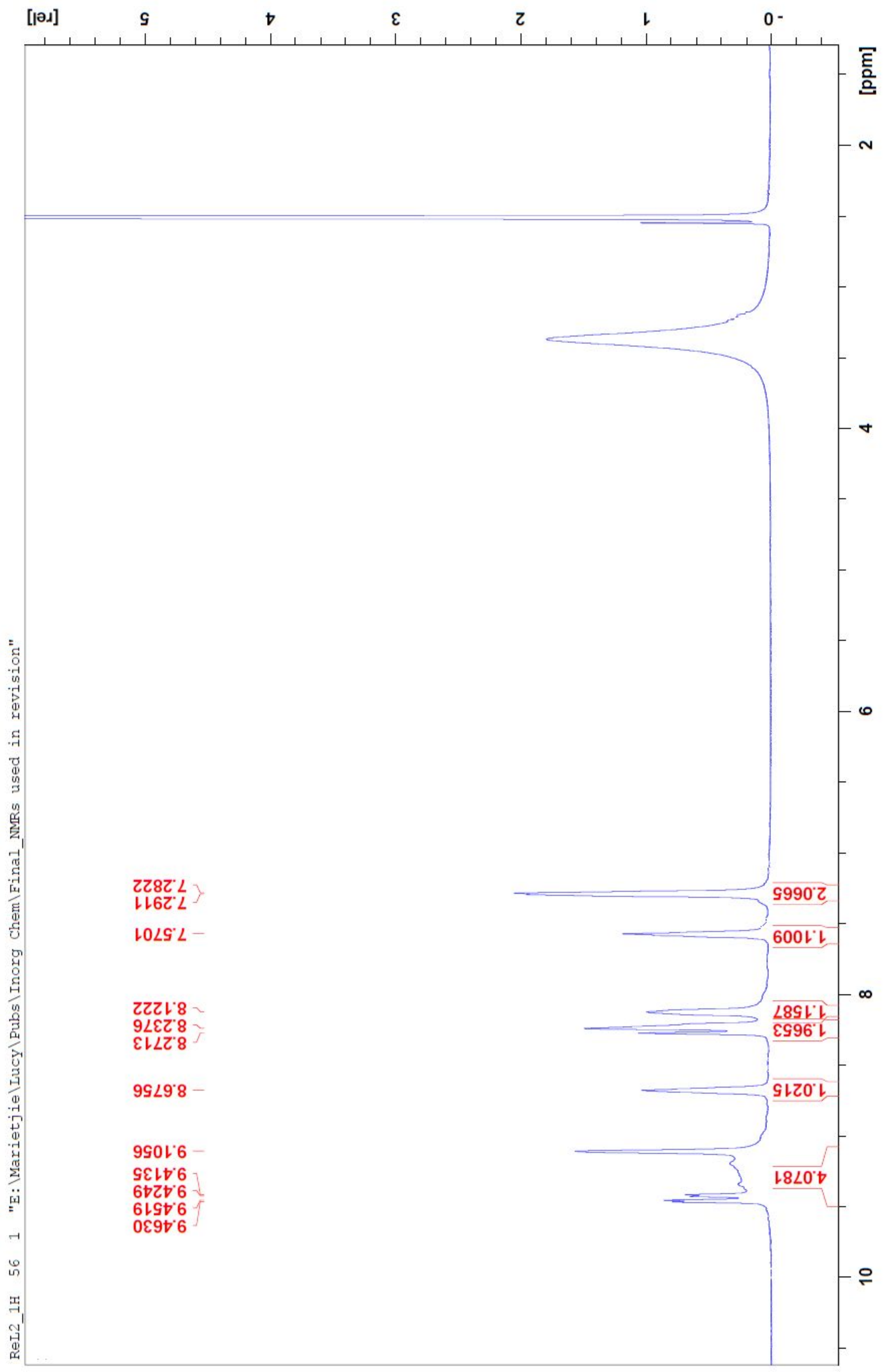

Figure S40. ^{1}H NMR spectra of **3**.

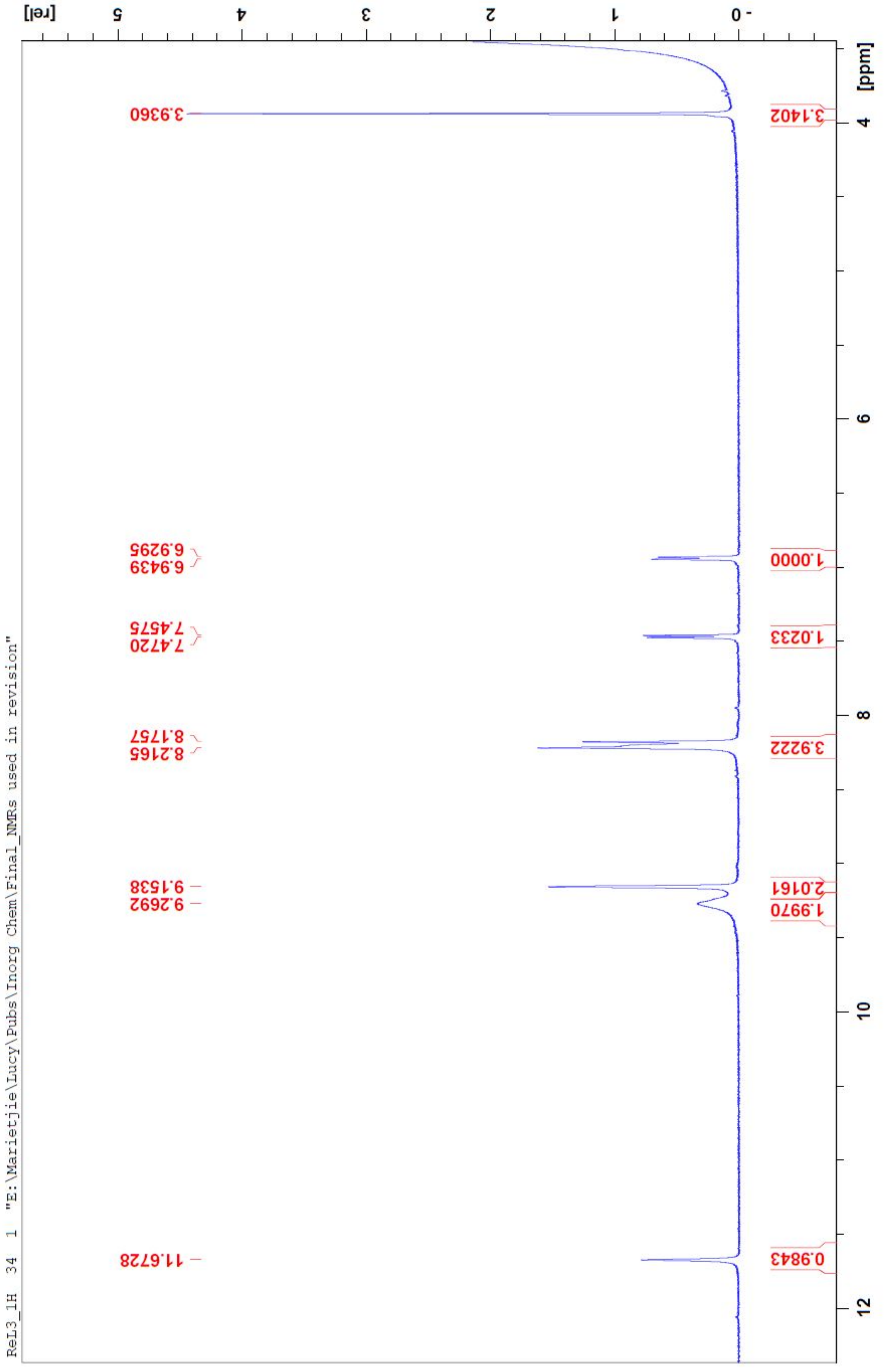

Figure S41. ^{1}H NMR spectra of **4**.

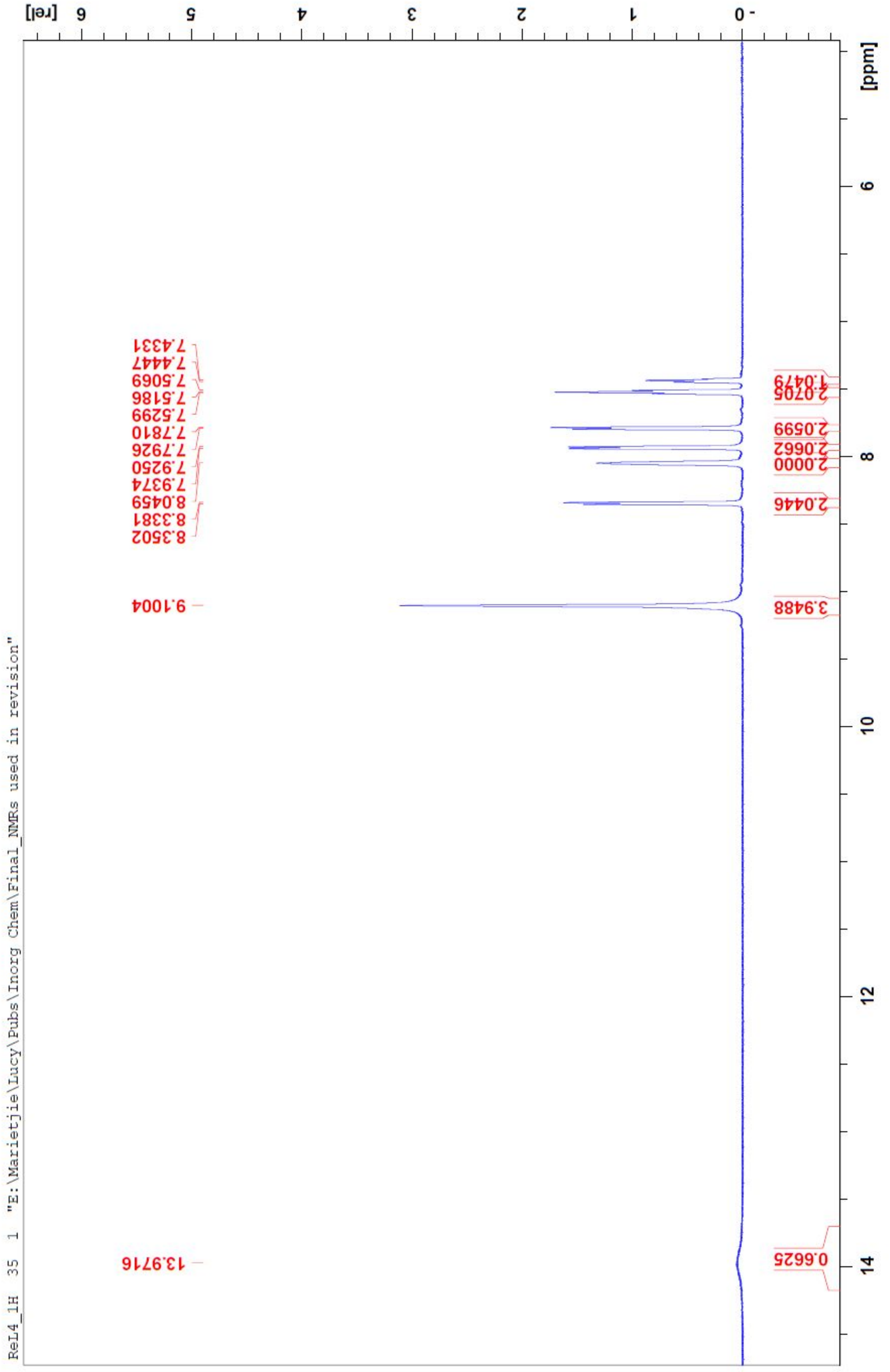

Figure S42. ^{1}H NMR spectra of **5**.

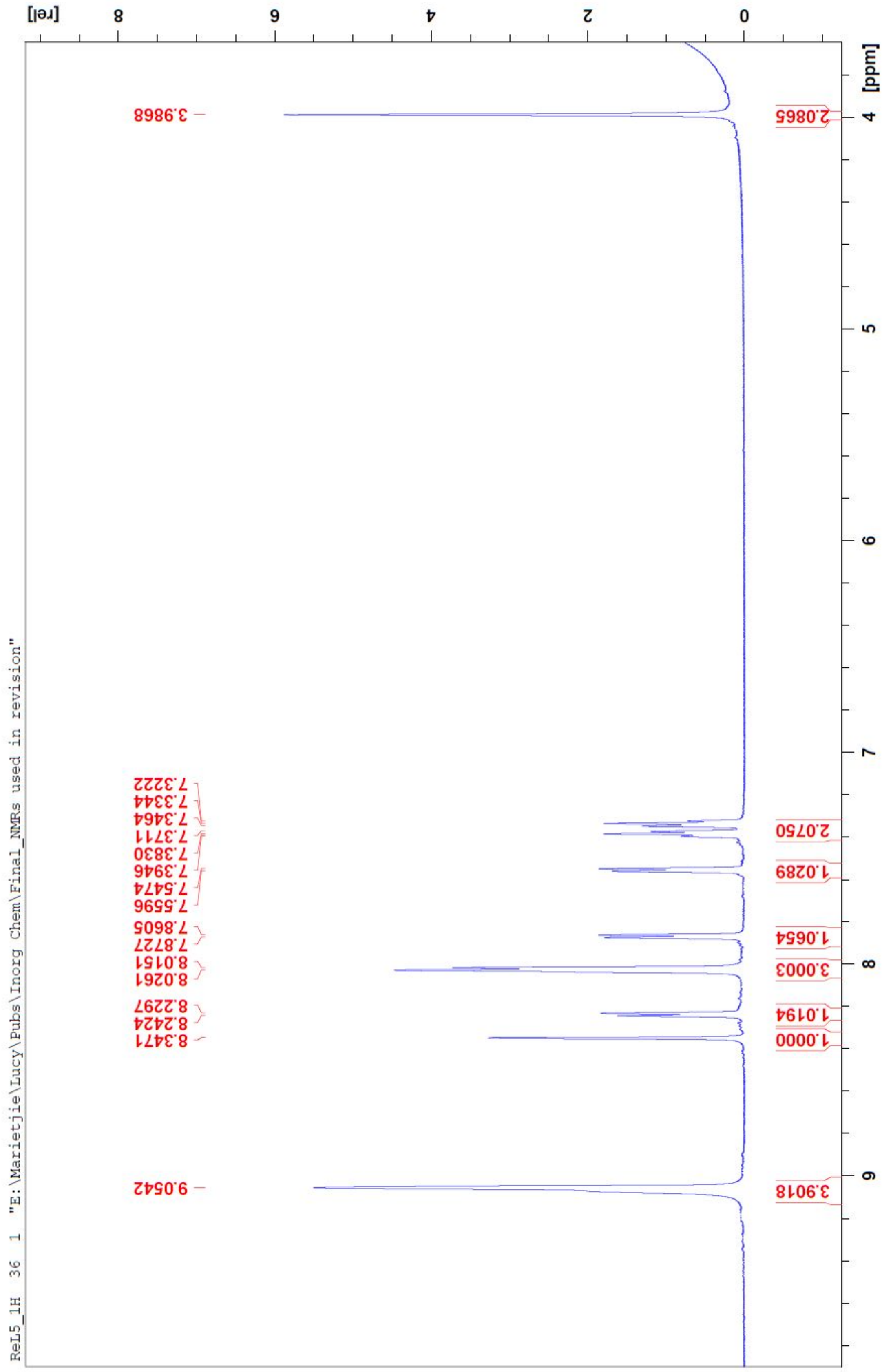

Figure S43. ^{1}H NMR spectra of **6**.

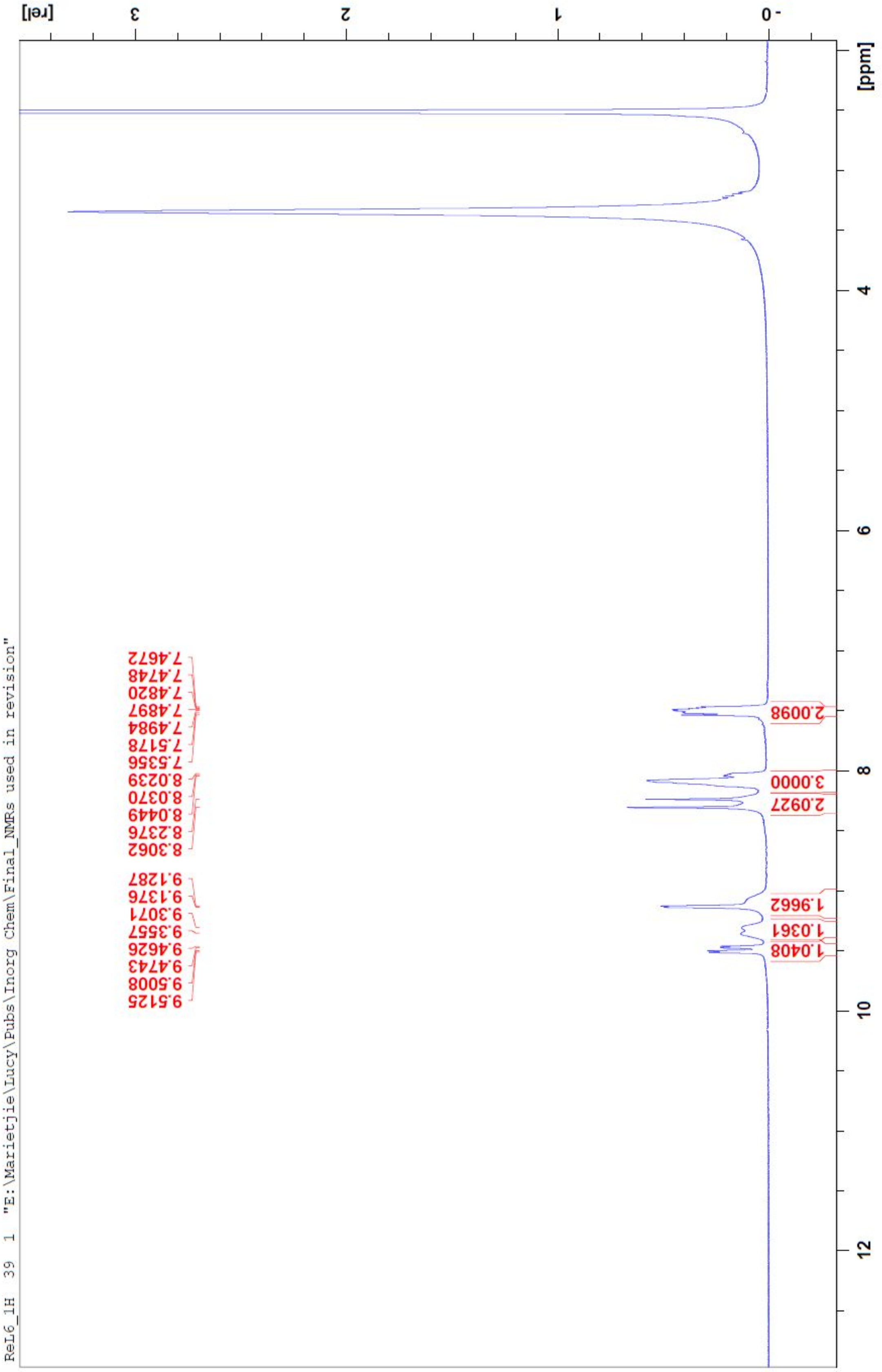

Figure S44. ^{1}H NMR spectra of **7**.

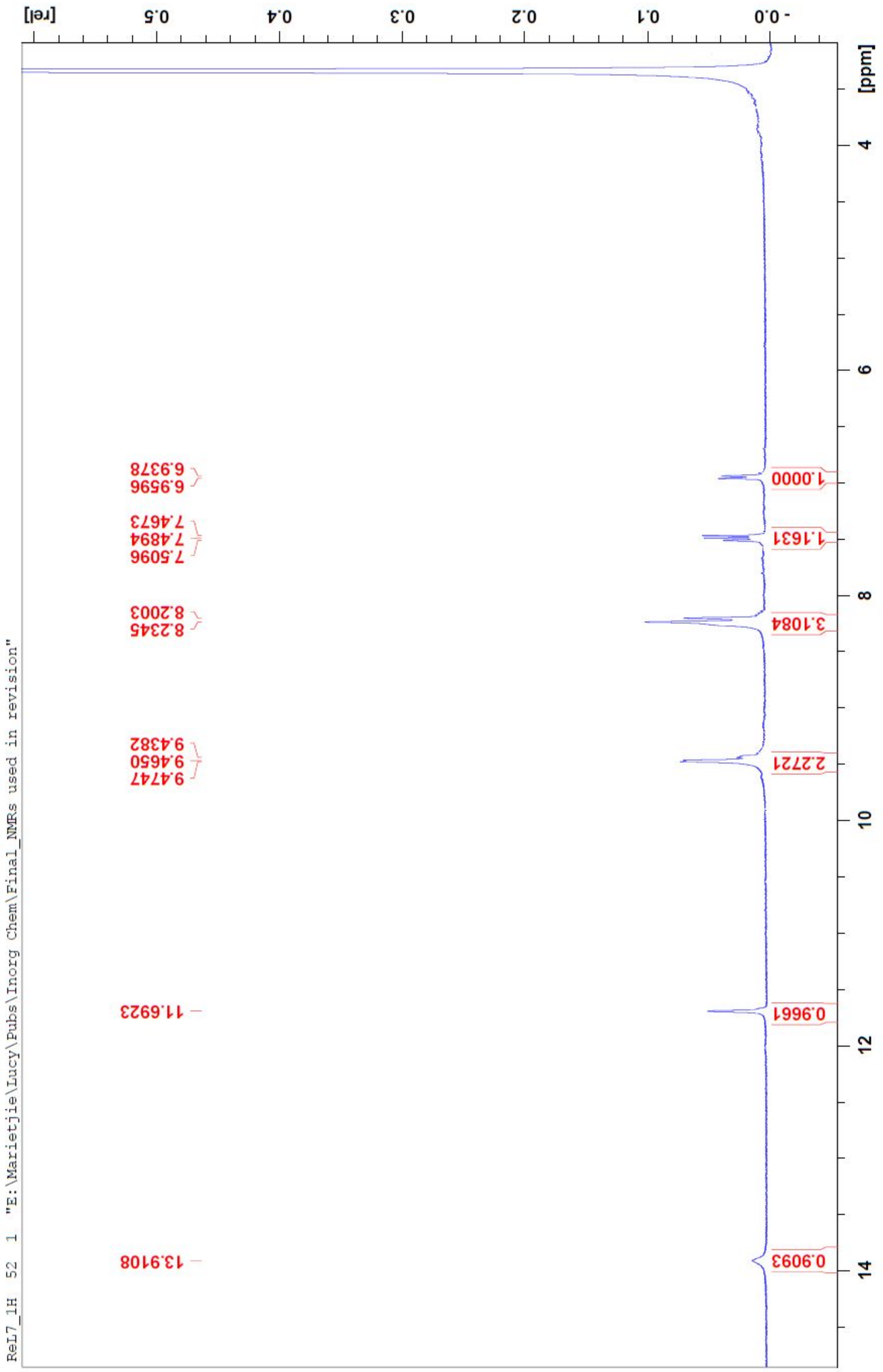

Figure S45. ^{1}H NMR spectra of **8**.

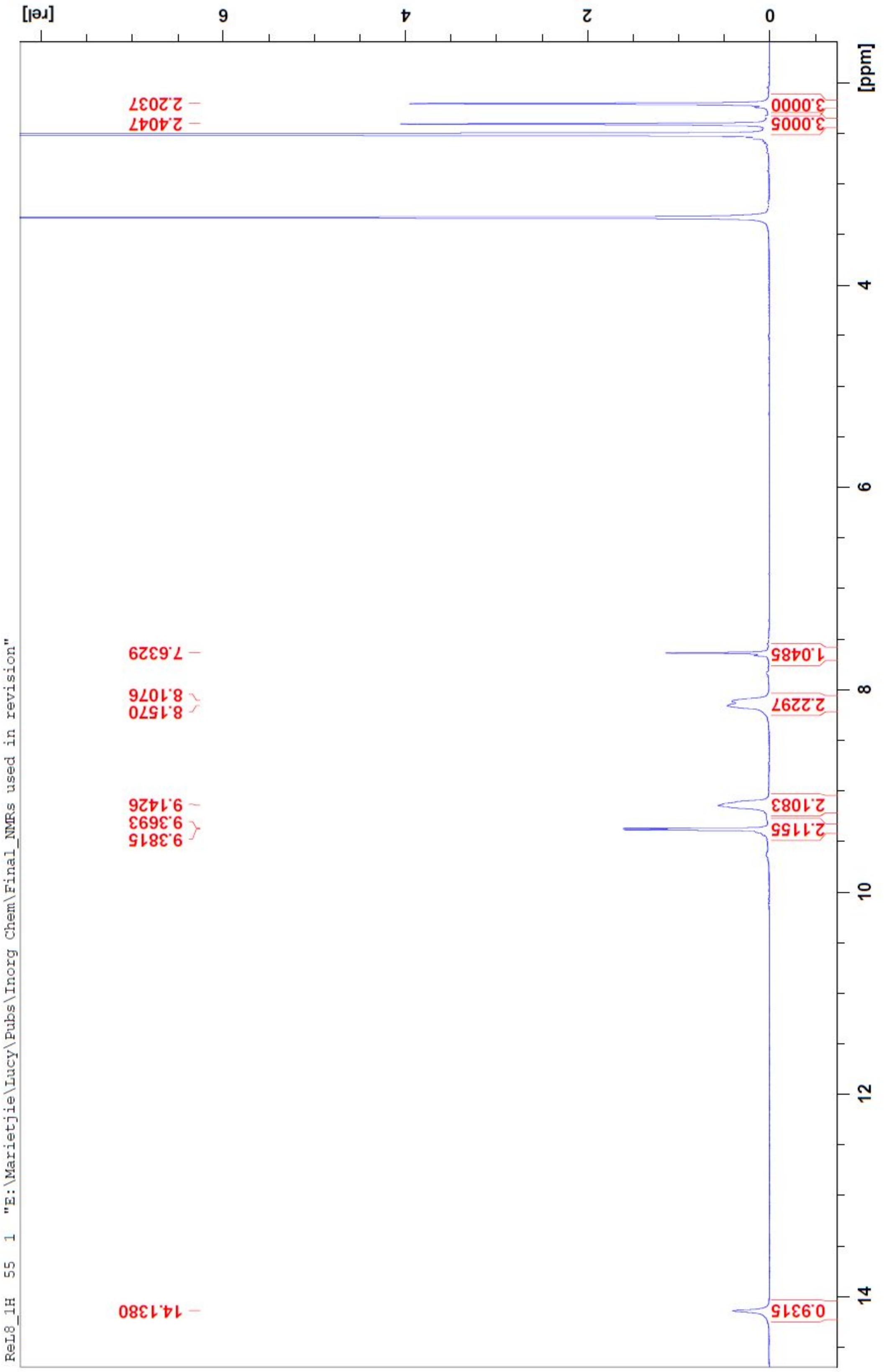

^{13}C NMR spectra of complexes

Figure S46. ^{13}C NMR spectra of **1**.

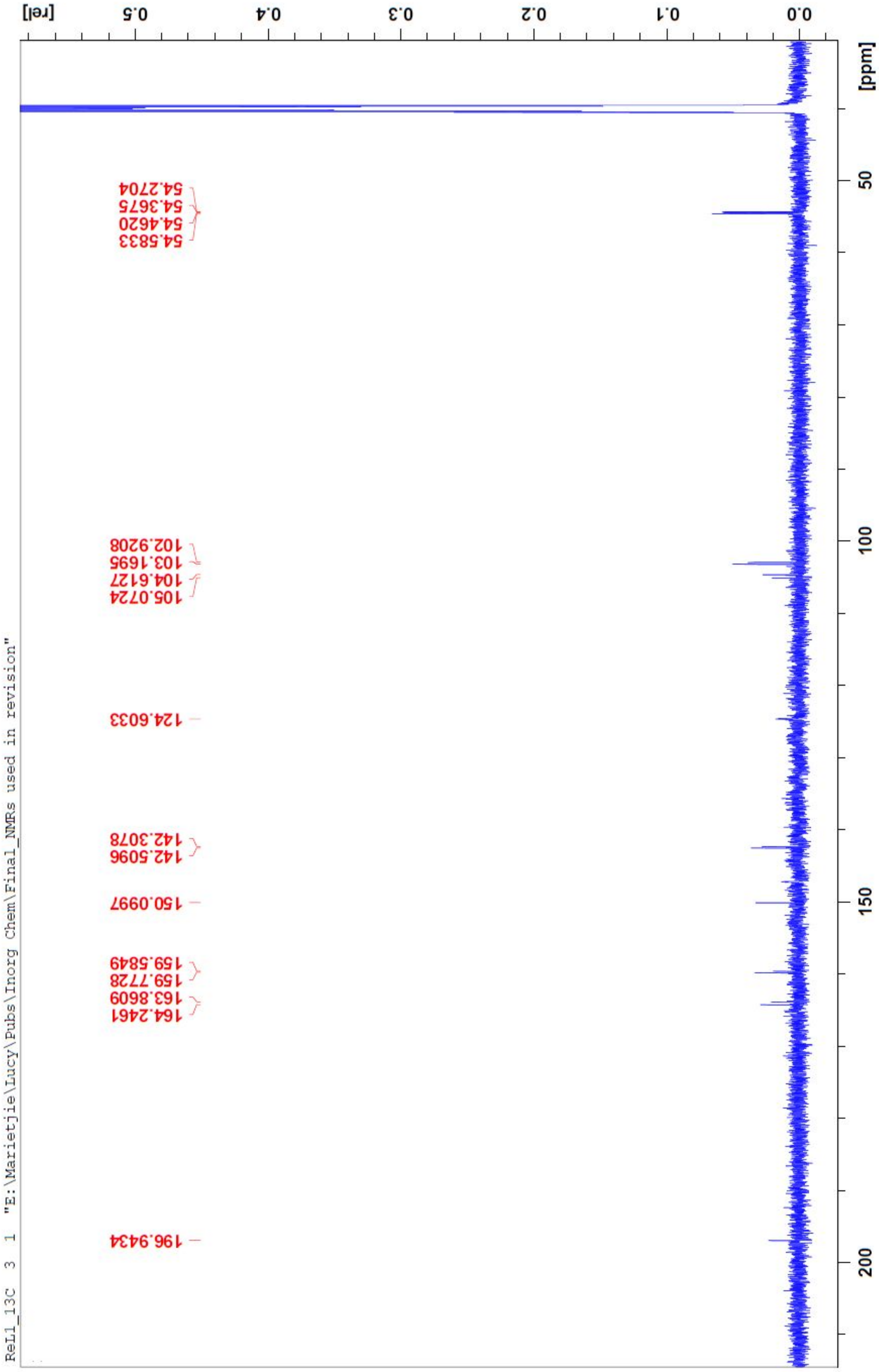

Figure S47. ^{13}C NMR spectra of **2**.

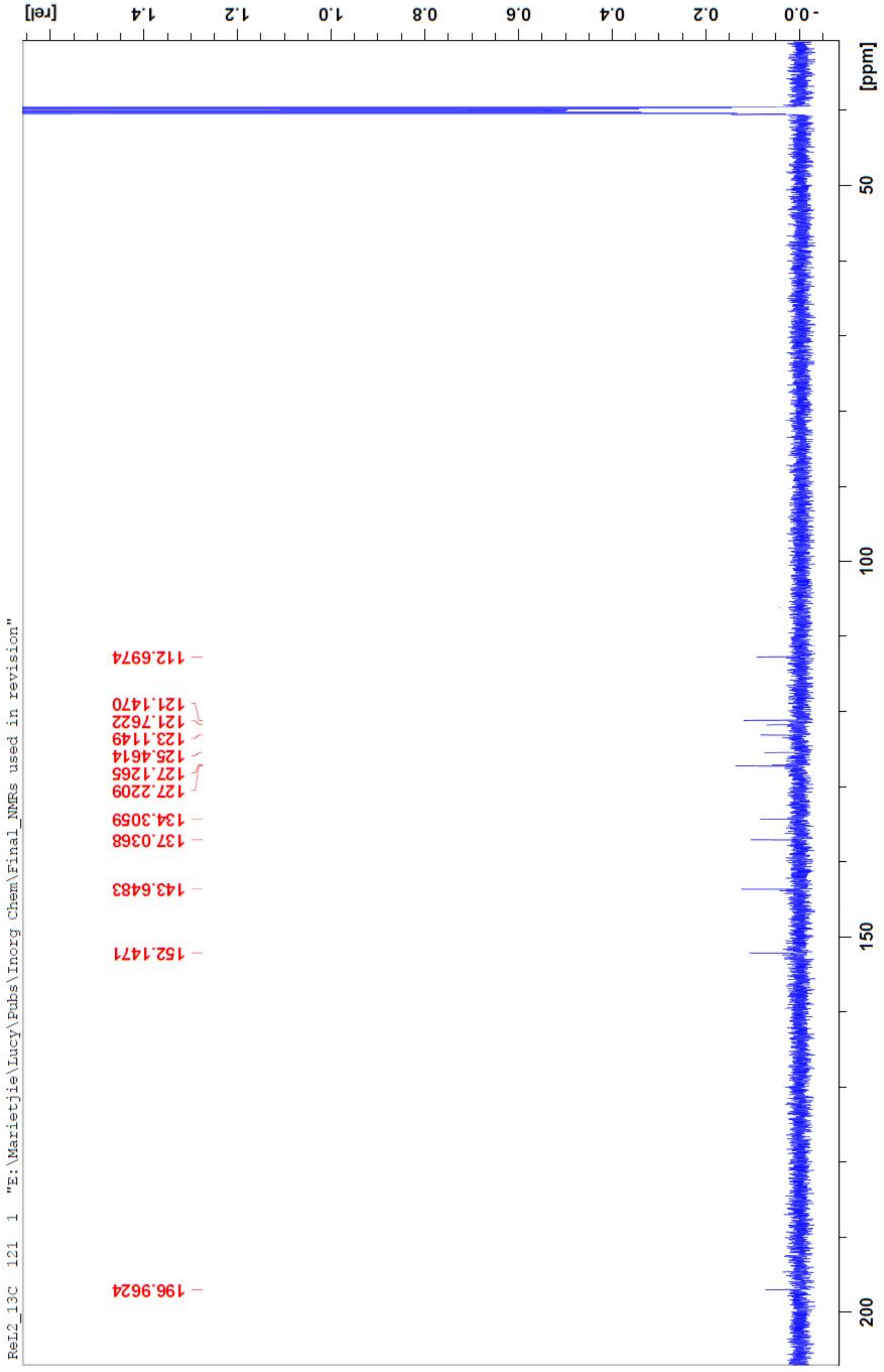

Figure S48. ^{13}C NMR spectra of **3**.

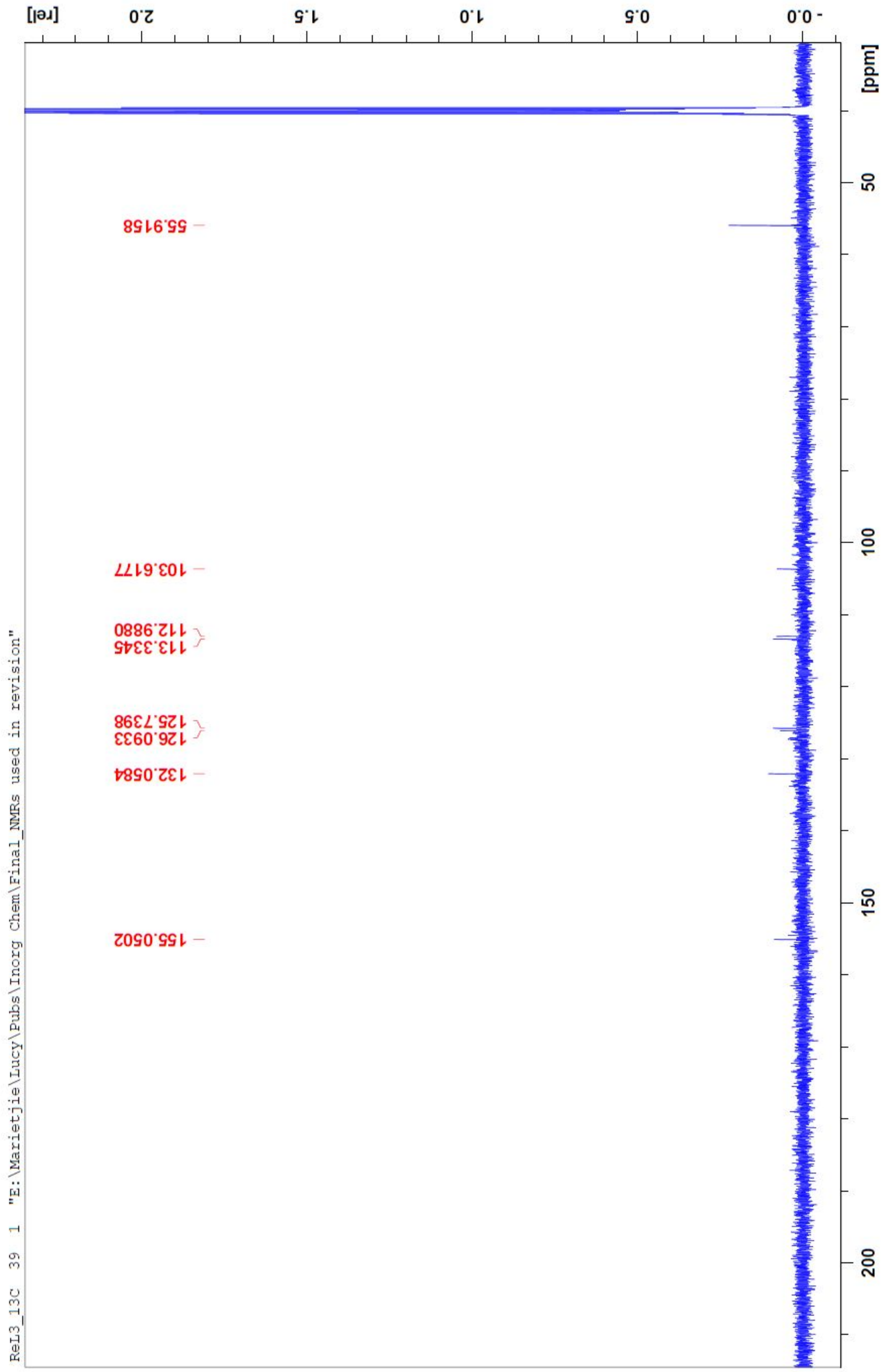

Figure S49. ^{13}C NMR spectra of **4**.

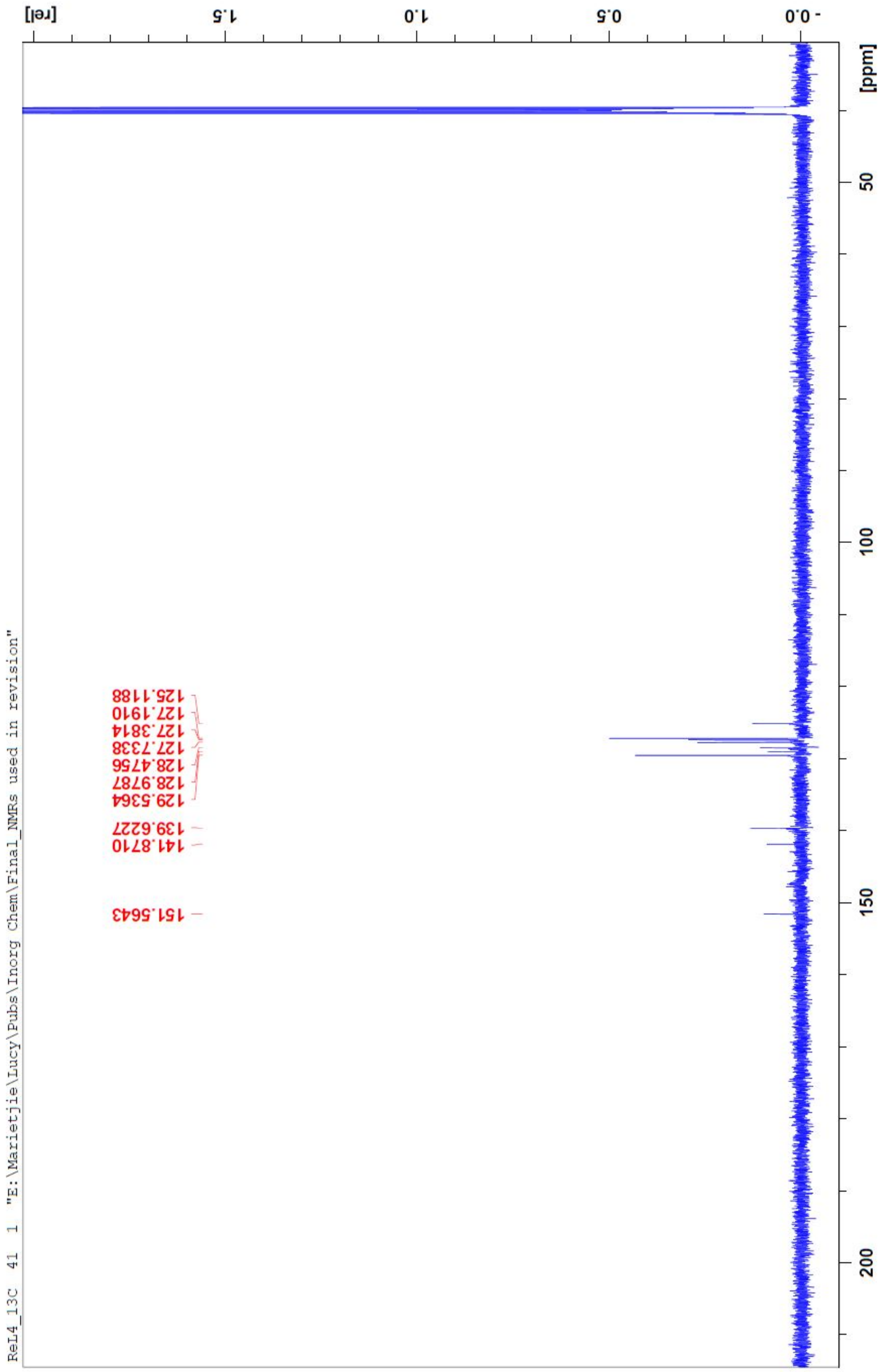

Figure S50. ^{13}C NMR spectra of **5**.

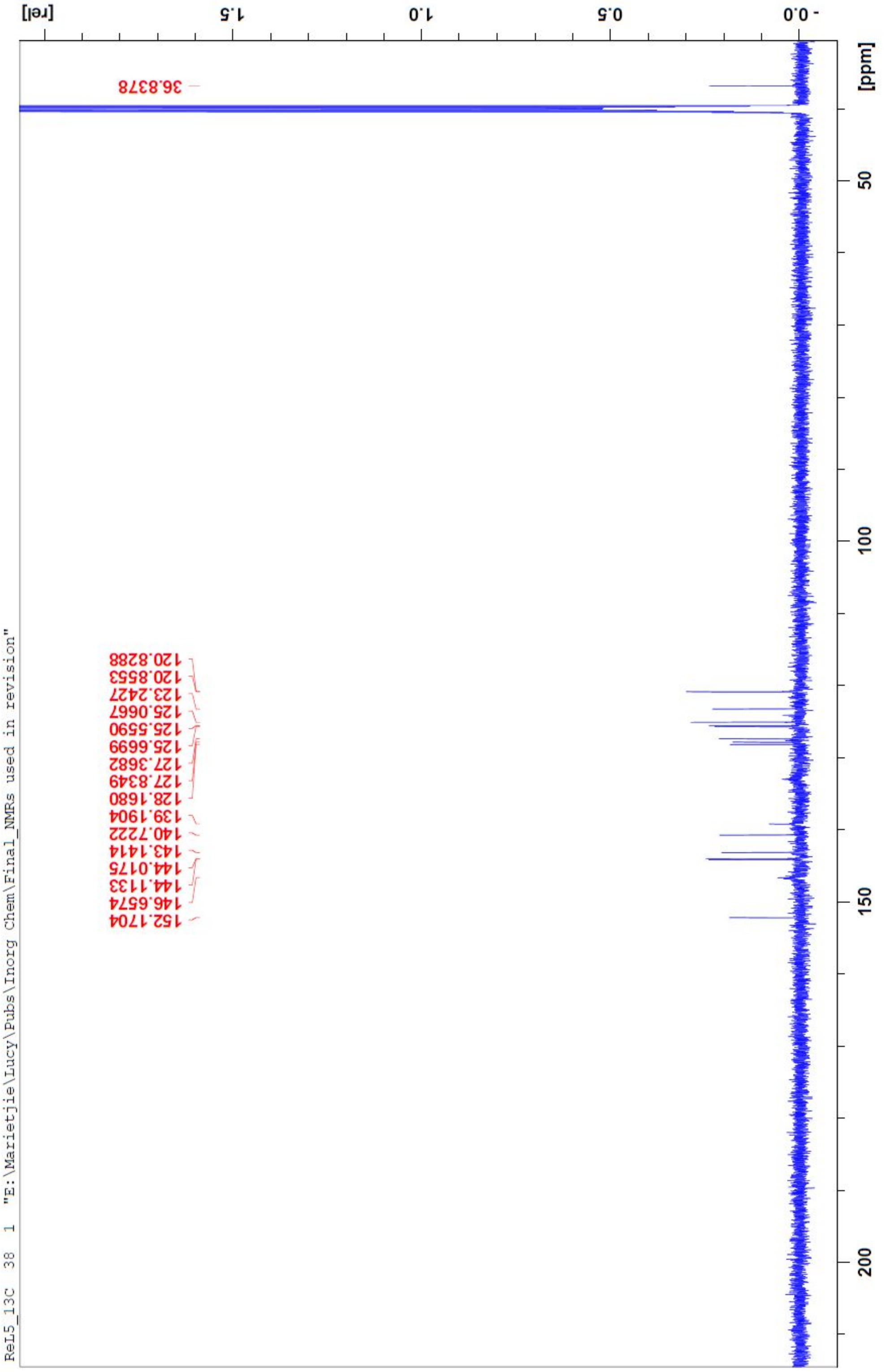

Figure S51. ^{13}C NMR spectra of **6**.

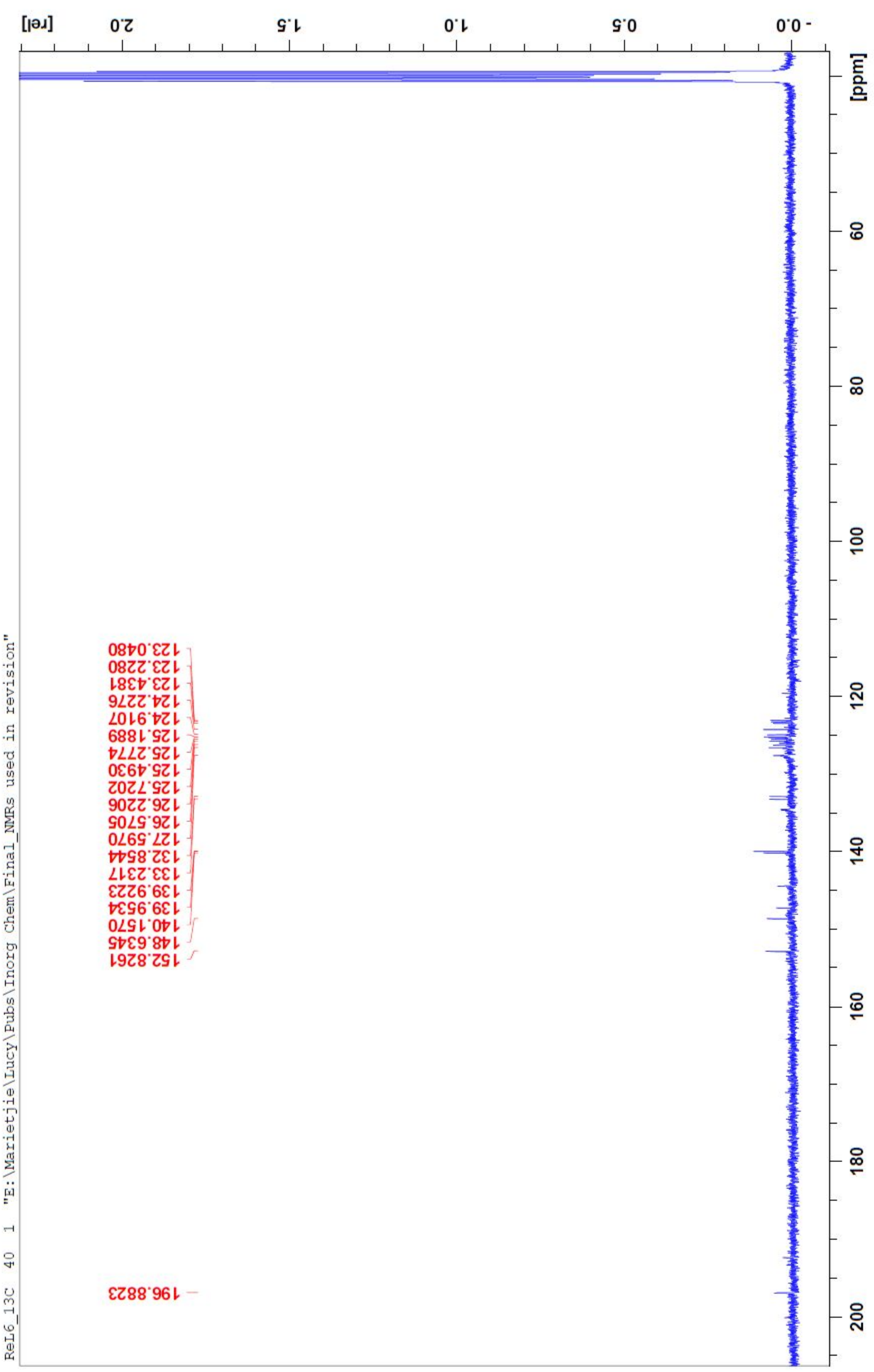

Figure S52. ^{13}C NMR spectra of **7**.

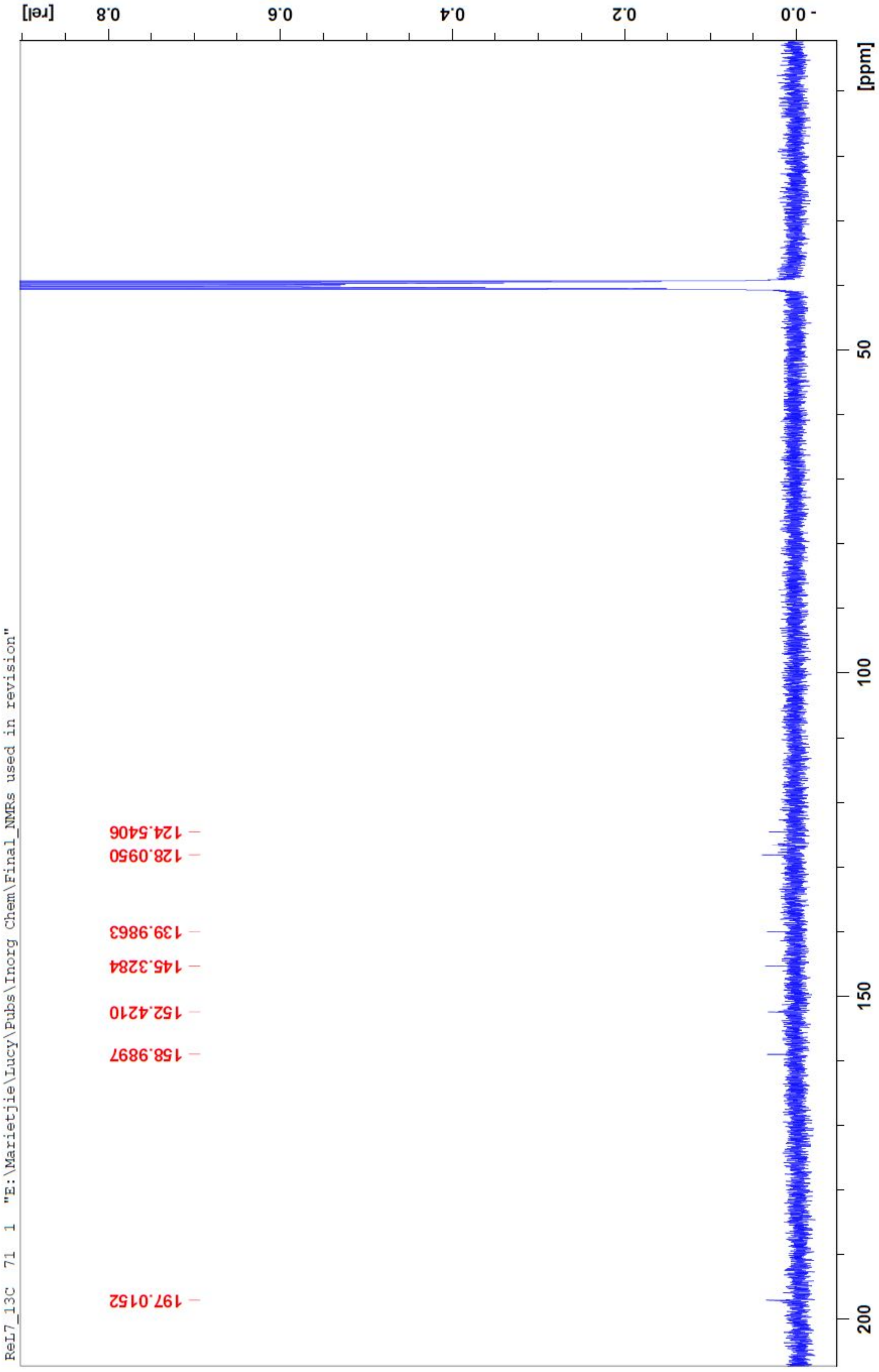

Figure S53. ^{13}C NMR spectra of **8**.

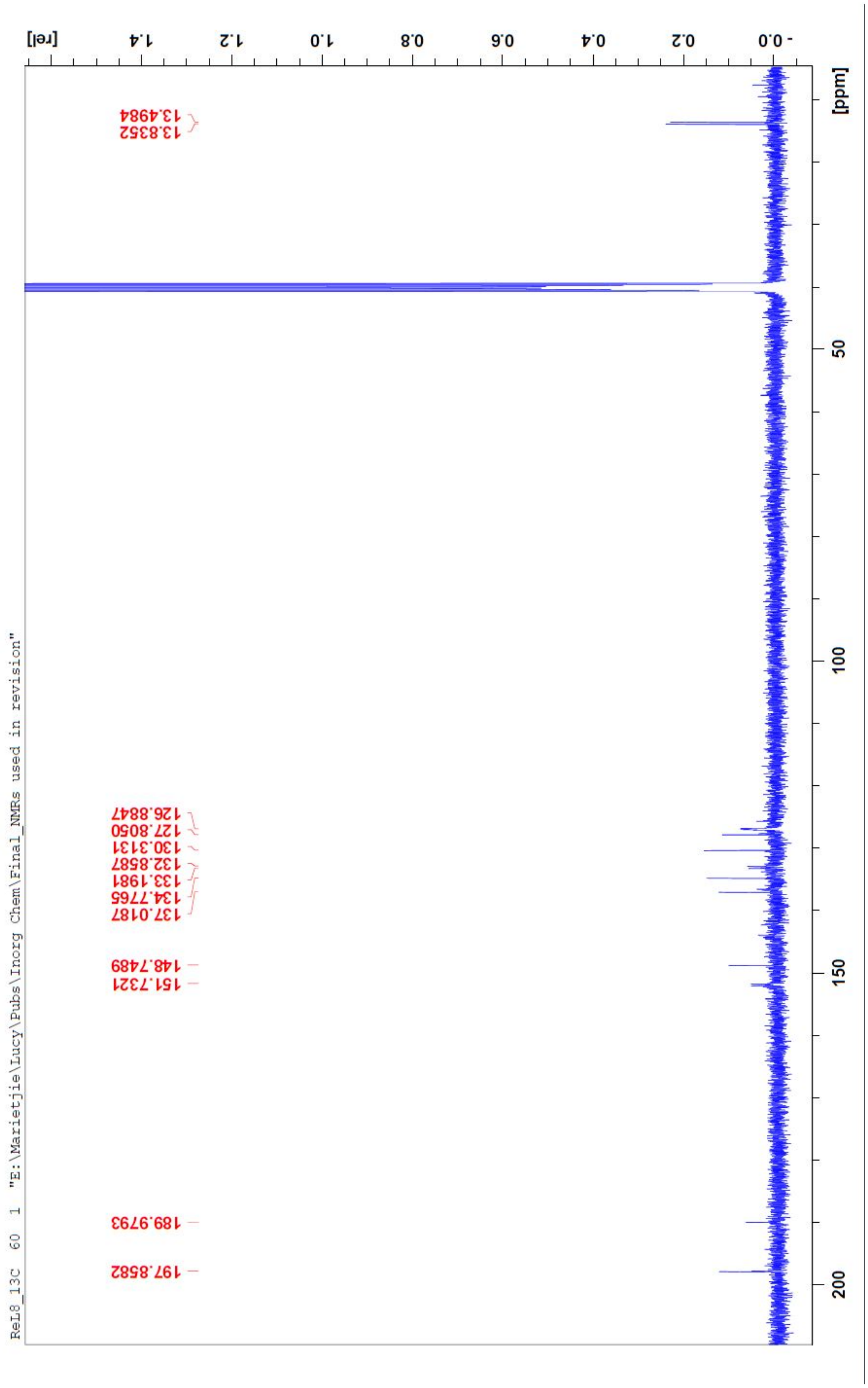

FT-IR spectra of complexes

Figure S54. IR spectra of **1**.

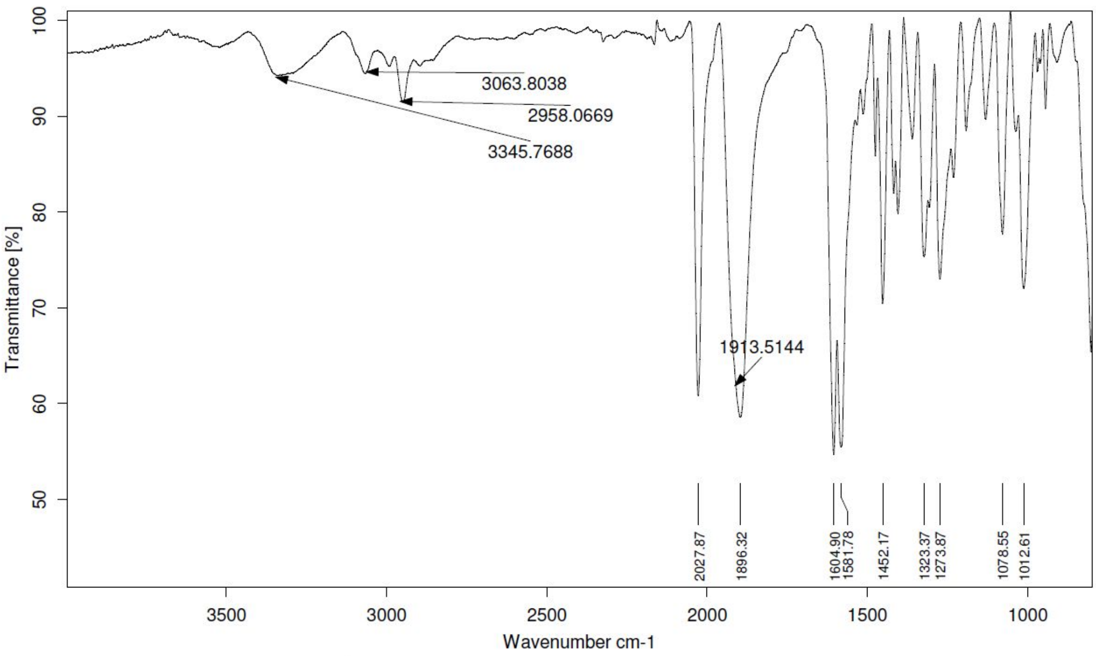

Path of File C:\IR DATA\LUCY	Filename Re(CO)3(LK_L2)H2O.0
Sample Name Re(CO)3(LK_L2)H2O	Sample Form Yellow solid
Date of Measurement 18/08/2020	Compound Name

Figure S55. IR spectra of **2**.

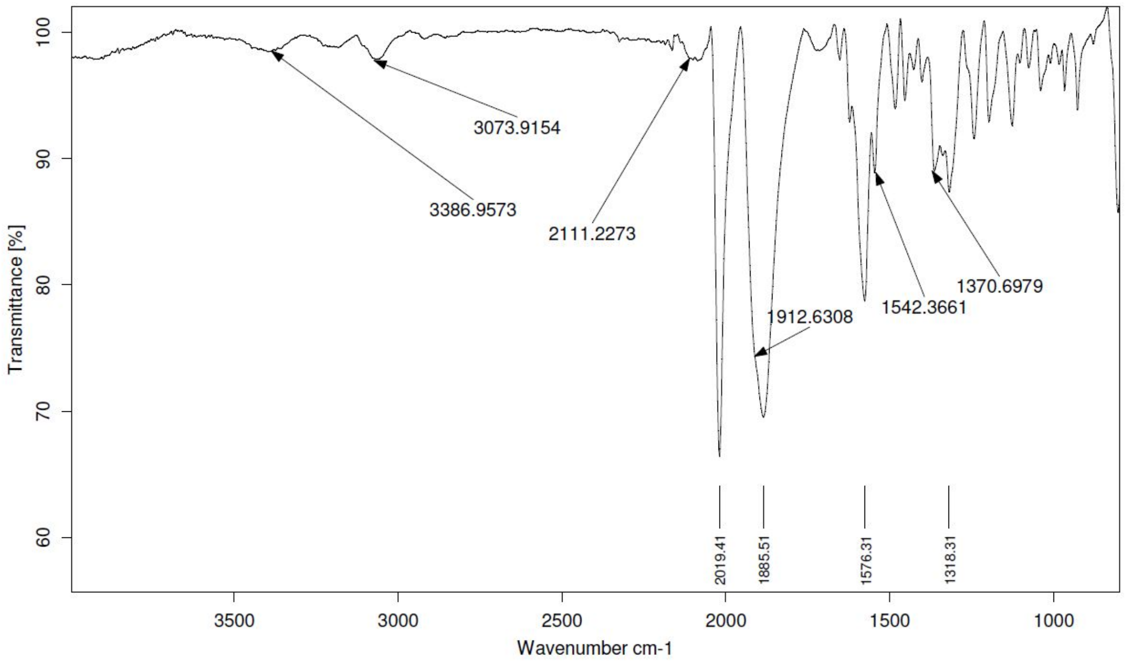

Path of File C:\IR DATA\LUCY	Filename Re(CO)3(LK_L3)H2O.0
Sample Name Re(CO)3(LK_L3)H2O	Sample Form Yellow/Brown solid
Date of Measurement 18/08/2020	Compound Name

Figure S56. IR spectra of **3**.

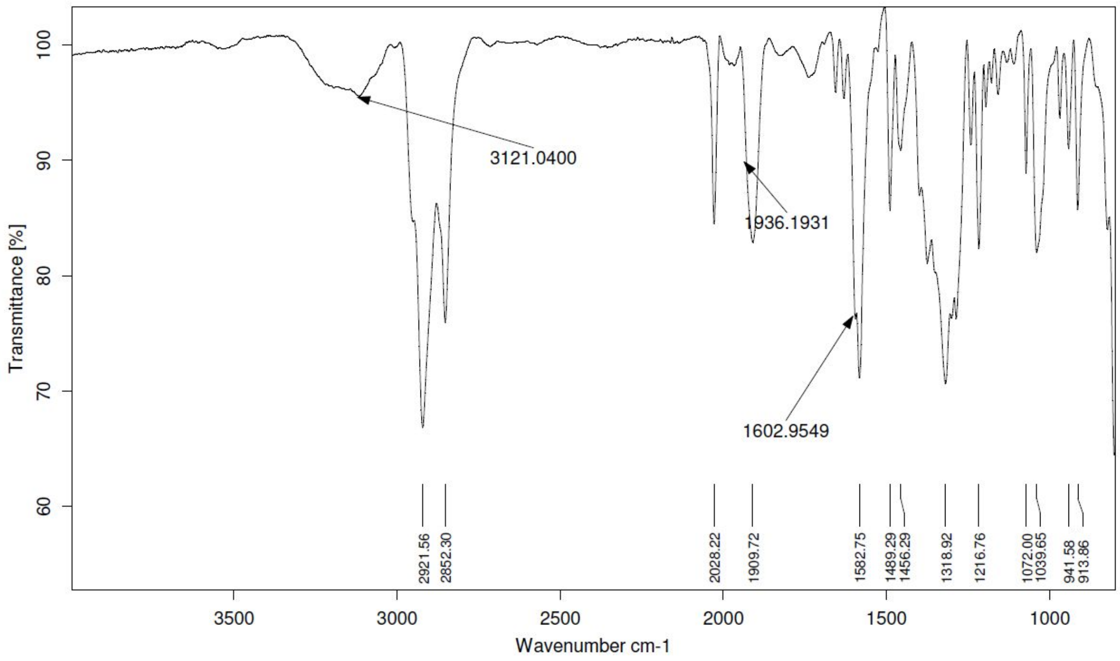

Path of File C:\IR DATA\LUCY	Filename Re(CO)3(LK_L4)H2O.0
Sample Name Re(CO)3(LK_L4)H2O	Sample Form Yellow/Brown solid
Date of Measurement 18/08/2020	Compound Name

Figure S57. IR spectra of **4**.

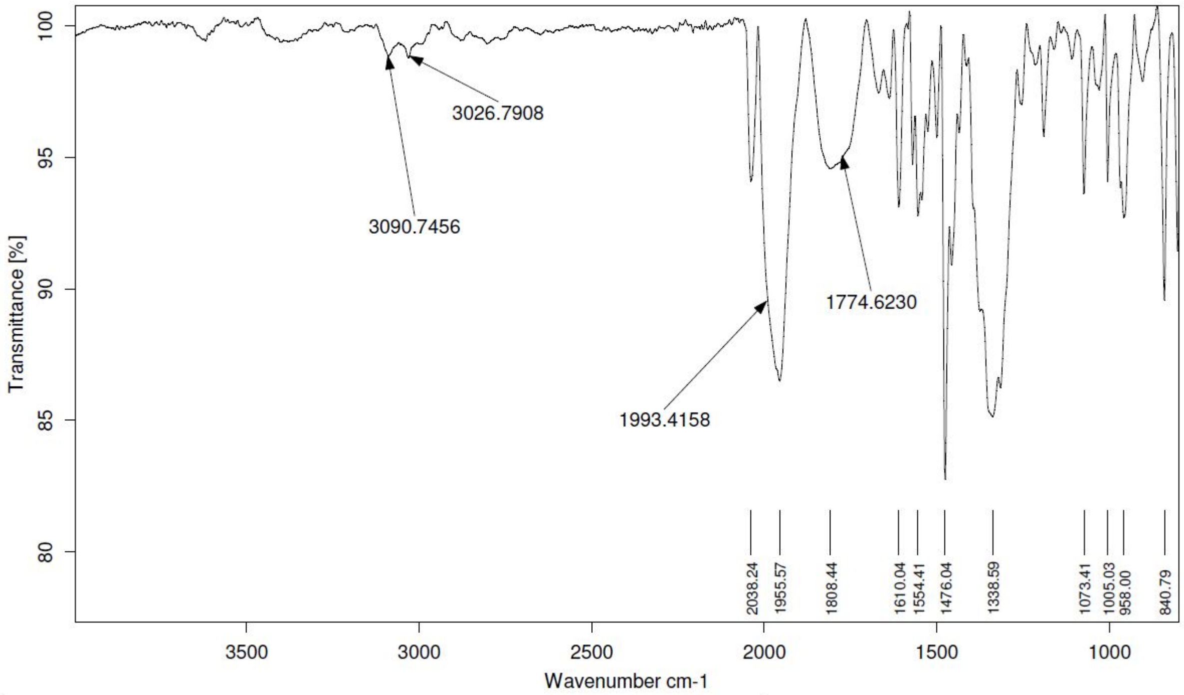

Path of File C:\IR DATA\LUCY	Filename Re(CO)3(LK_L5)H2O.0
Sample Name Re(CO)3(LK_L5)H2O	Sample Form Yellow/Brown solid
Date of Measurement 18/08/2020	Compound Name

Figure S58. IR spectra of **5**.

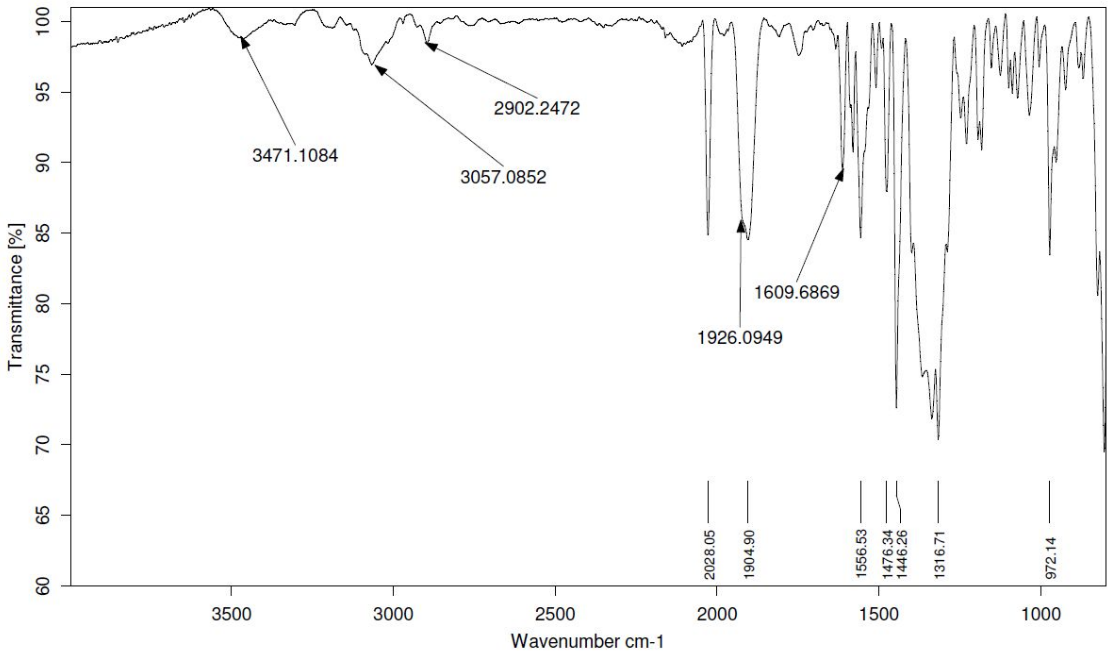

Path of File C:\IR DATA\LUCY	Filename Re(CO)3(LK_L6)H2O.0
Sample Name Re(CO)3(LK_L6)H2O	Sample Form orange solid
Date of Measurement 18/08/2020	Compound Name

Figure S59. IR spectra of **6**.

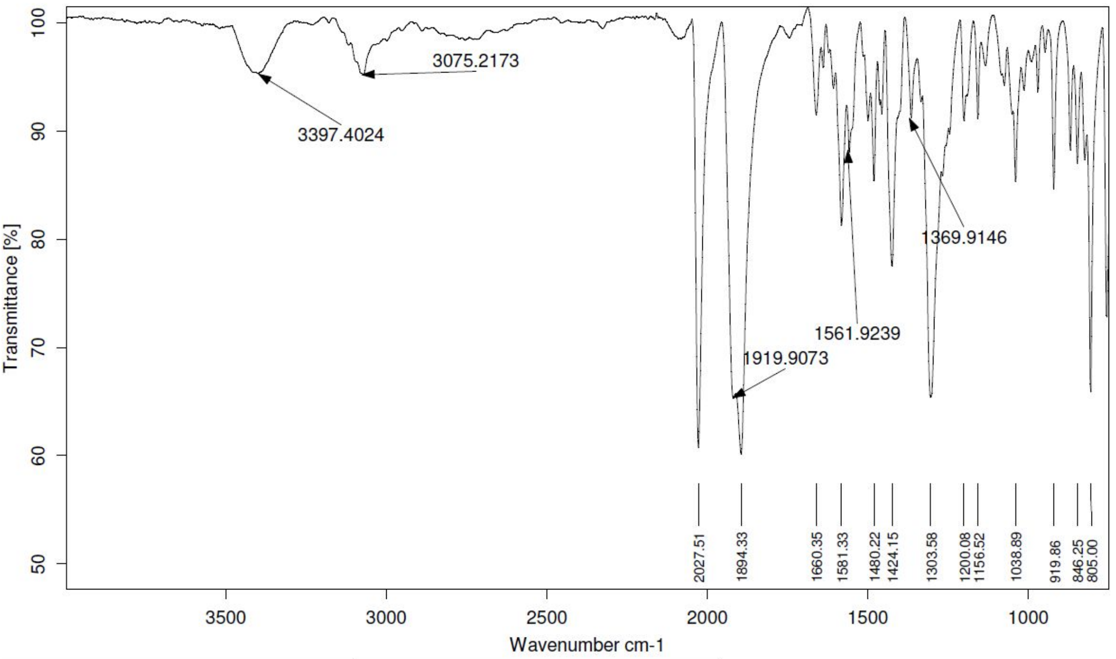

Path of File C:\IR DATA\LUCY	Filename Re(CO)3(LK_L7)H2O.0
Sample Name Re(CO)3(LK_L7)H2O	Sample Form yellow solid
Date of Measurement 26/08/2020	Compound Name

Figure S60. IR spectra of **7**.

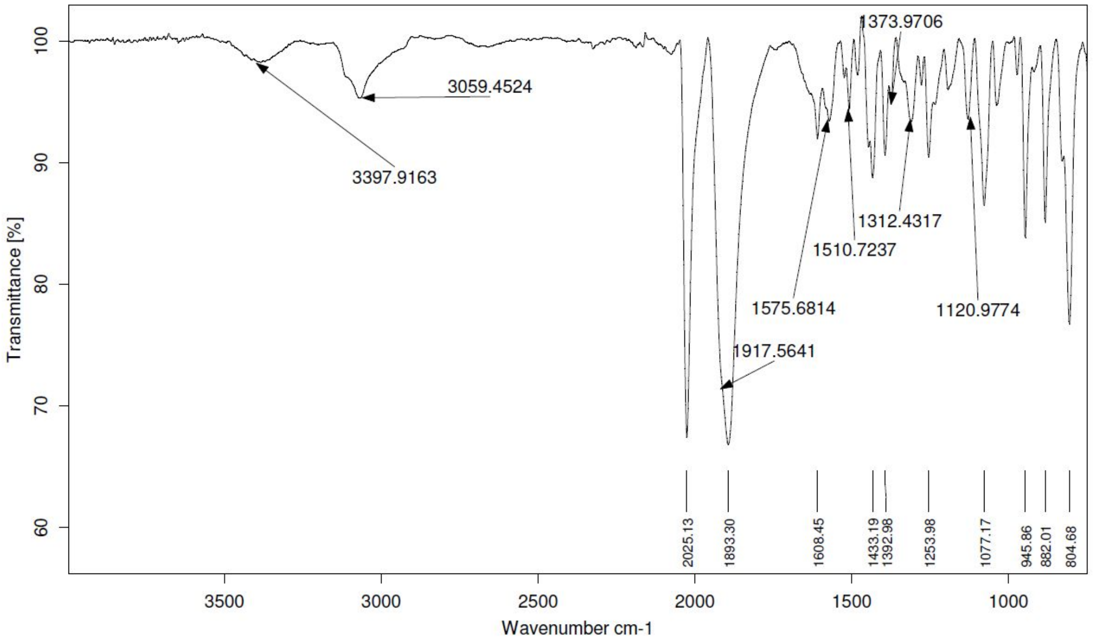

Path of File C:\IR DATA\LUCY	Filename Re(CO)3(LK_L8)H2O.0
Sample Name Re(CO)3(LK_L8)H2O	Sample Form Brown solid
Date of Measurement 26/08/2020	Compound Name

Figure S61. IR spectra of **8**.

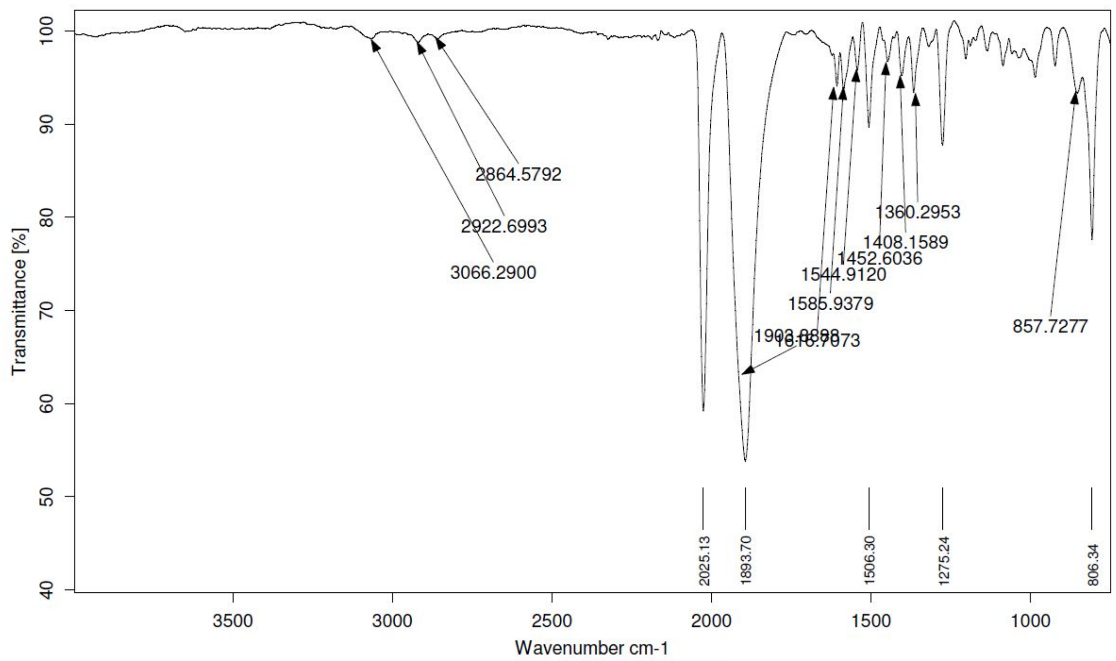

Path of File C:\IR DATA\LUCY	Filename Re(CO)3(LK_L9)H2O.0
Sample Name Re(CO)3(LK_L9)H2O	Sample Form Orange solid
Date of Measurement 26/08/2020	Compound Name

ESI-MS spectra of complexes

Figure S62. ESI-MS spectra of **1.**

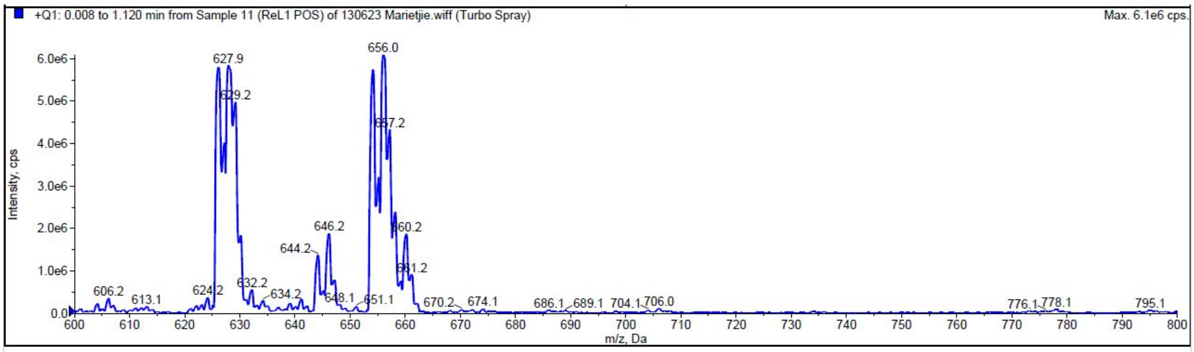

Figure S63. ESI-MS spectra of **2.**

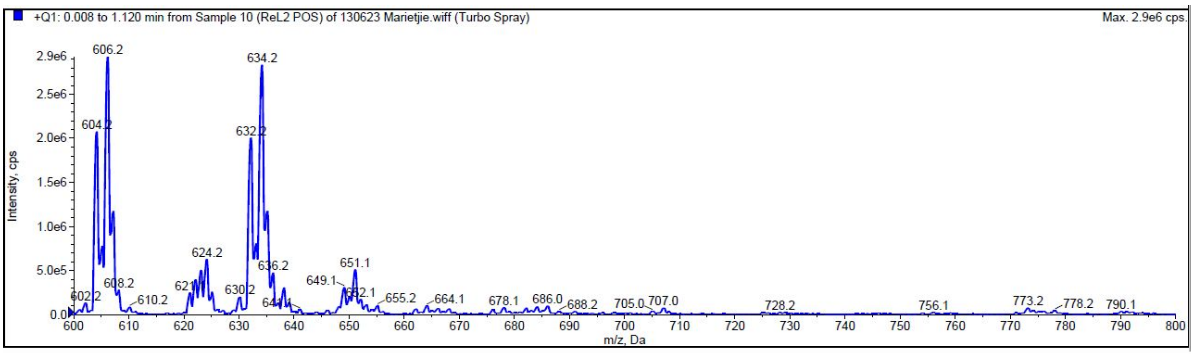

Figure S64. ESI-MS spectra of **3**.

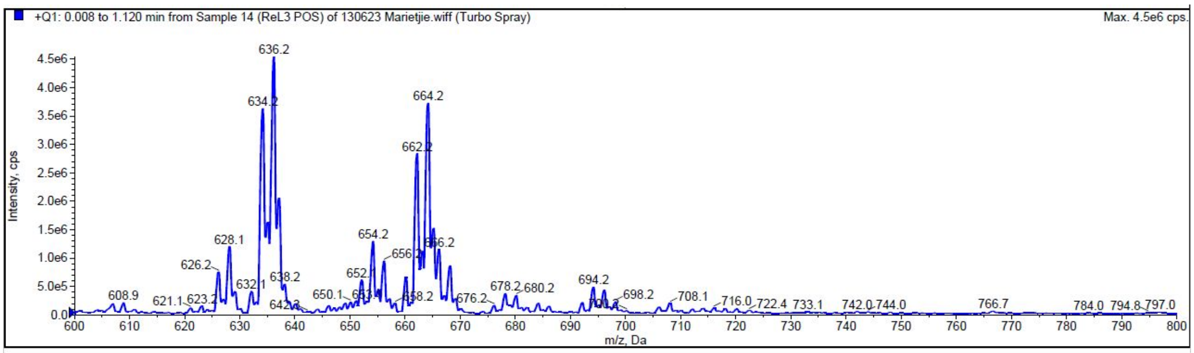

Figure S65. ESI-MS spectra of **4**.

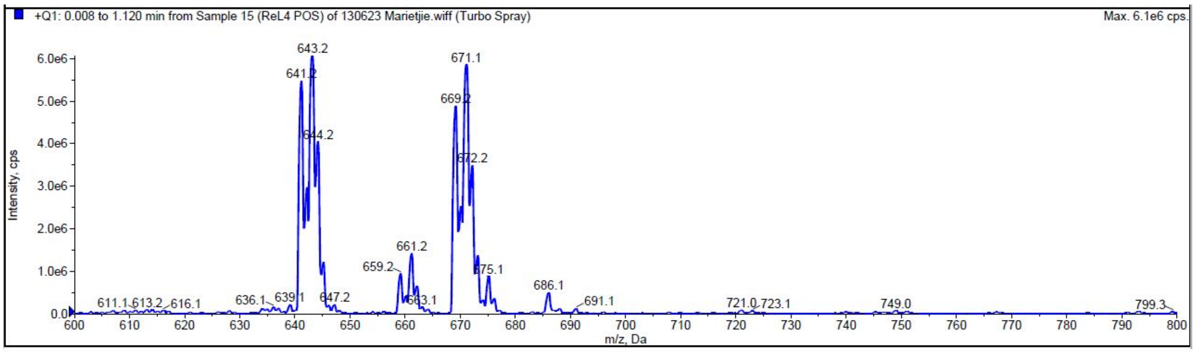

Figure S66. ESI-MS spectra of **5**.

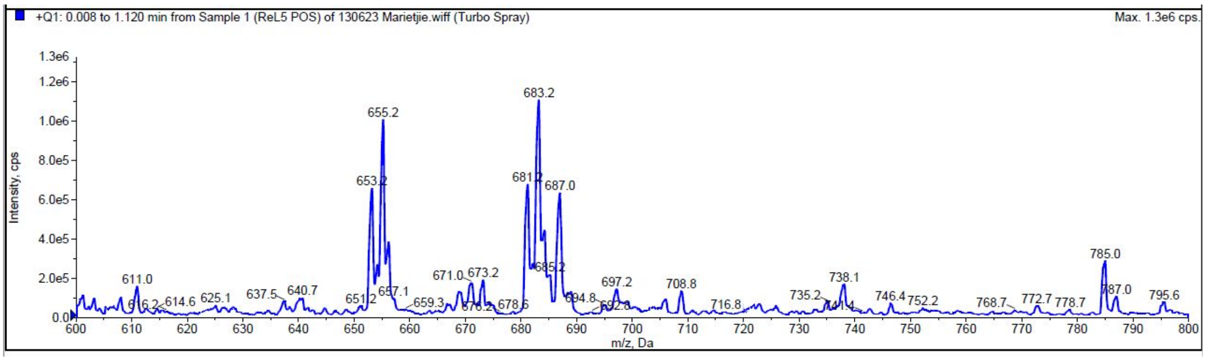

Figure S67. ESI-MS spectra of **6.**

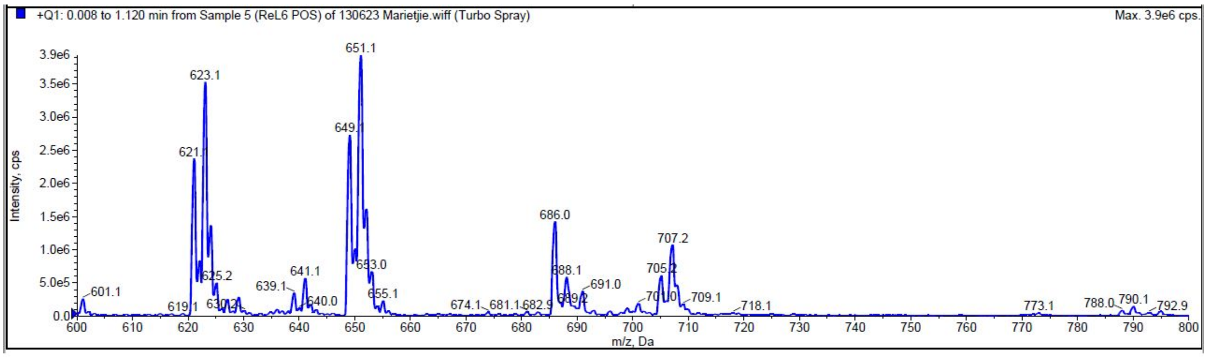

Figure S68. ESI-MS spectra of **7.**

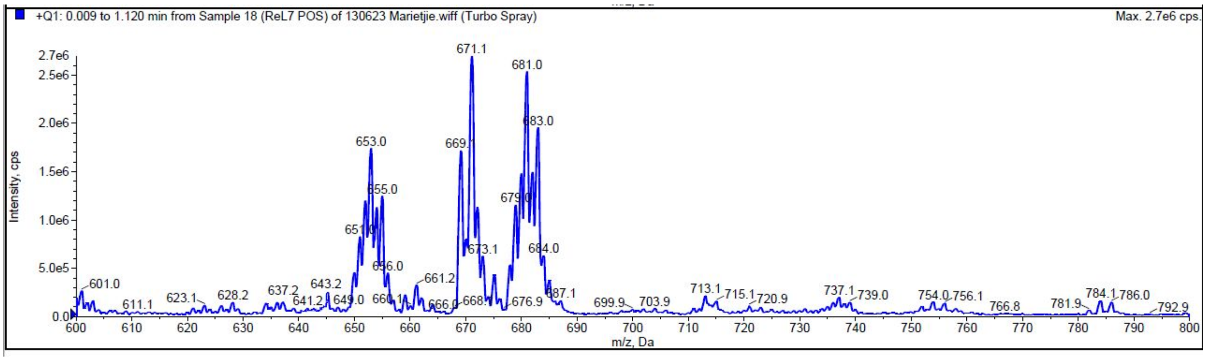

Figure S69. ESI-MS spectra of **8.**

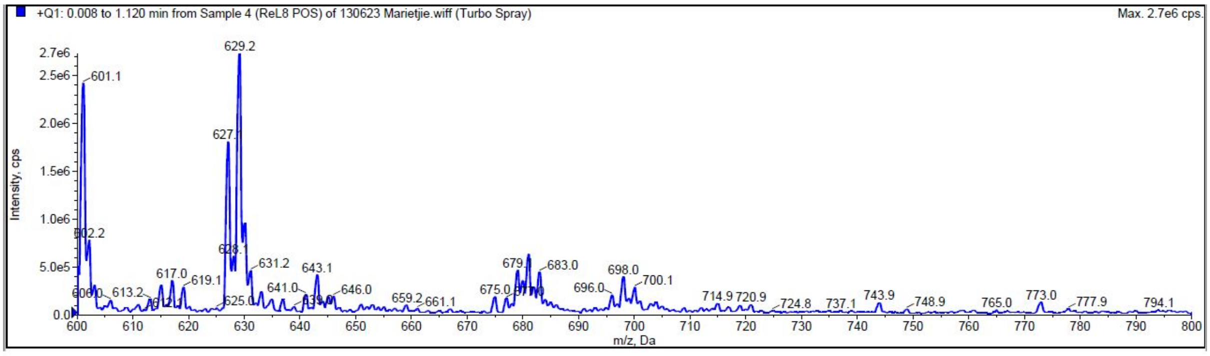

---

[1] Hunter, F.W.; Wouters, B.G.; Wilson, W.R. Hypoxia-activated prodrugs: paths forward in the era of personalised medicine. *British J. Cancer* **2016**, *114*, 1071-1077. doi: 10.1038/bjc.2016.79.

[2] Li, Y.; Zhao, L.; Li, X.-F. Targeting hypoxida: hypoxia-activated prodrugs in cancer therapy. *Front. Oncol.* **2021**, *11*, 700407. doi: 10.3389/fonc.2021.700407.
